# Sentiment Analysis of Acceptance TVET Online Courses on the Skill Academy App from Google Play: Leveraging Text Mining with Comparison Machine Learning Model

**DOI:** 10.12688/f1000research.181728.1

**Published:** 2026-06-09

**Authors:** Darmono Darmono, Yanuar Agung Fadlullah, Khakam Ma’ruf, Muhamad Riyan Maulana, Apry Aditya Saputra, Ramzy Bin Sulaiman, Bagus Banjar Bagaskara, Rizal Justian Setiawan, Sahril Sahril

**Affiliations:** 1 Civil Engineering Education, Faculty of Engineering, Universitas Negeri Yogyakarta, Yogyakarta, Special Region of Yogyakarta, 55281, Indonesia; 2 Technology and Vocational Education, Universitas Negeri Yogyakarta, Yogyakarta, Special Region of Yogyakarta, 55281, Indonesia; 3 Industrial Engineering, Faculty of Engineering, Universitas Gadjah Mada, Yogyakarta, Special Region of Yogyakarta, 55281, Indonesia; 4 Mechanical Engineering Education, Faculty of Engineering, Universitas Negeri Yogyakarta, Yogyakarta, Special Region of Yogyakarta, 55281, Indonesia; 5 Information Technology, Faculty of Engineering, Universitas Gadjah Mada, Yogyakarta, Special Region of Yogyakarta, 55281, Indonesia; 6 Asia and China Studies, College of Law and Politics, National Chung Hsing University, Taichung, Taichung City, 402204, Taiwan; 7 Industrial Engineering and Management, College of Engineering, Yuan Ze University, Zhongli District, Taoyuan City, 32003, Taiwan

**Keywords:** Machine Learning, Sentiment Analysis, Skill Academy App, Text Mining, TVET Acceptance, VADER Sentiment Analyzer.

## Abstract

**Background:**

The rapid growth of online learning platforms has transformed Technical and Vocational Education and Training, enabling broader access to skill based education through mobile applications. Understanding user acceptance is essential to ensure the sustainability and effectiveness of digital TVET platforms. User reviews available on application marketplaces provide valuable insights into learners’ perceptions, satisfaction, and challenges encountered during use.

**Methods:**

This study analyzed 3,000 user reviews of the Skill Academy application collected from the Google Play Store. Text preprocessing included data cleaning, case folding, tokenization, filtering, stemming, and translation into English to ensure compatibility with the Valence Aware Dictionary and Sentiment Reasoner. Sentiment labels were generated using VADER and categorized into positive, neutral, and negative classes. Text features were extracted using Term Frequency Inverse Document Frequency. Six machine learning classifiers were evaluated, namely Naive Bayes, Support Vector Machine, Logistic Regression, Random Forest, Decision Tree, and K Nearest Neighbors, using a 70:30 train test split.

**Results:**

The sentiment analysis results showed that 69.4 percent of the reviews expressed positive sentiment, 20.5 percent were neutral, and 10.1 percent were negative. Positive reviews predominantly emphasized course usefulness, ease of use, and skill development benefits. Negative reviews were mainly associated with technical issues such as application errors and performance limitations. Among the evaluated models, the Support Vector Machine achieved the best performance, with an accuracy of 85.77 percent and an area under the curve value of 0.97.

**Conclusions:**

The findings indicate a high level of user acceptance of online TVET courses offered through the Skill Academy application, primarily driven by content quality and usability. Nevertheless, addressing technical performance issues remains essential to improve user satisfaction and support sustainable long term adoption. The results also demonstrate that the Support Vector Machine model is highly effective for sentiment classification in the context of online TVET platforms.

## 1. Introduction

Digital transformation has changed the global education landscape, particularly in Technical and Vocational Education and Training (TVET). ^1, 2^ Digital transformation creates unprecedented access opportunities through online learning platforms. ^3^ Mobile applications have become the primary medium for delivering educational content, allowing users to access quality skills training anytime, anywhere. ^4, 5^ In Southeast Asia, particularly Indonesia, the adoption of online learning platforms for TVET has accelerated significantly over the past five years. ^6, 7^ Skill Academy, as one of Indonesia’s leading TVET online course platforms integrated with the Google Play Store ecosystem, has attracted thousands of users seeking quality vocational skills training. ^8^ With exponential user growth, the volume of user reviews on Google Play Store has also increased significantly, creating a rich source of data for understanding user perceptions, satisfaction levels, and acceptance of TVET content quality and application features. ^9^^,^^10^

Understanding users’ perceptions and sentiments toward online learning platforms is a crucial step for the continuous improvement of digital vocational education services. ^11, 12^ User reviews stored on the Google Play Store contain authentic feedback about the learning experience, course content quality, teaching effectiveness, and overall user satisfaction. ^13, 14^ However, application developers and education stakeholders face substantial challenges in extracting meaningful insights from large volumes of unstructured review data ^15^. ^16, 17^ Therefore, sentiment analysis and machine learning applications to classify user review sentiment into positive, negative, and neutral categories are practical solutions for transforming raw review data into actionable insights for application developers and TVET stakeholders. ^18, 19^

Sentiment analysis, also known as opinion mining, is a subdiscipline of Natural Language Processing (NLP) that focuses on the automatic identification, extraction, and classification of opinions and emotions contained in text. ^20^ This technique has been proven effective in various practical application domains, including customer feedback analysis, product evaluation, and understanding public perceptions of digital services and education. ^21, 22^ Research by Marry A et al. 2025 shows that sentiment analysis can provide actionable insights into student satisfaction, the effectiveness of online learning, and the quality of educational content. ^23^ Sharin et al. 2025 analyzed the sentiment of TVET discussions on social media and found that machine learning-based algorithms can identify sentiment patterns with good performance, opening up opportunities for sentiment analysis in TVET education application reviews. ^24^ For feature extraction from text, previous studies have widely used VADER (Valence Aware Dictionary and Sentiment Reasoner), a lexicon-based sentiment analysis tool specifically optimized for social media text, with the ability to capture sentiment intensity through the analysis of capitalization, punctuation, and intensifier words. ^25, 26^ VADER has been proven to deliver consistent and reliable results for sentiment analysis, particularly on social media texts and user reviews. ^27, 28, 29^

In developing a sentiment analysis model for user reviews, the selection of machine learning algorithms is an important factor that affects the accuracy of sentiment classification and the quality of insights produced. ^30^ Various machine learning algorithms are widely used for text classification tasks, including sentiment analysis, each with its own unique characteristics, advantages, and limitations. Naive Bayes is a simple yet powerful algorithm, providing a solid baseline that remains relevant due to its computational efficiency, especially for high-dimensional datasets. ^31^^,^^32^ Support Vector Machine (SVM) is a simple yet powerful algorithm, providing a solid baseline that remains relevant due to its computational efficiency, especially for high-dimensional datasets. ^33, 34^ Logistic Regression offers an attractive combination of competitive accuracy and high interpretability, making it easier to understand the factors that influence sentiment classification results. ^35^ Decision tree-based algorithms such as Random Forest and Decision Tree provide flexibility in handling non-linear relationships and interactions between features; Random Forest generally shows solid performance and is often a strong baseline in sentiment classification tasks. ^36, 37^ Meanwhile, K-Nearest Neighbors (KNN) as a non-parametric algorithm is capable of capturing local patterns in data, but has high computational complexity in large datasets, because distance calculations must be performed for each prediction. ^36, 38^ Although these algorithms have been extensively developed and studied in various domains, their systematic comparison specifically for classifying user sentiment toward online TVET learning platforms such as Skill Academy is still very limited in the literature.

User reviews on Google Play Store for educational apps contain important information about how users perceive the quality of online learning, course content, app features, and overall user experience. ^39, 40^ Positive sentiment in reviews reflects ease of access, ease of use, flexibility of time, and comprehensiveness of materials, all of which contribute to a good learning experience. ^41^ Negative sentiment reveals technical issues, usability, content, and mismatches between user expectations and reality. ^42, 43^ Neutral sentiment usually reflects a more balanced view and is neither completely satisfied nor disappointed. ^44^ By analyzing this distribution and pattern of sentiment, application developers can identify areas of strength that need to be maintained and areas of weakness that require priority improvement. ^45, 46^ In addition, insights into user sentiment can help TVET developer stakeholders understand how actual users perceive the quality and effectiveness of online vocational learning in Indonesia. ^47^

There is a significant research gap in the analysis of user sentiment towards online TVET platforms in Indonesia, especially the Skill Academy application, which has become one of the leading vocational learning platforms. ^48^ Previous research on sentiment analysis in the education sector has generally focused more on analyzing feedback on general social media, without paying particular attention to online TVET platforms. ^49^ In addition, many studies have examined international education platforms without considering the specific context of Indonesia and TVET. Several studies also used a single algorithm or only conducted limited comparisons, without systematically comparing the various algorithms available. In addition, there is still very limited understanding of how different machine learning algorithms classify user sentiment toward the quality of online TVET learning, and which algorithms are most effective in this context. This study fills that gap by conducting a systematic comparative study using six standard machine learning algorithms (Naive Bayes, SVM, Logistic Regression, Random Forest, Decision Tree, and KNN) to analyze the sentiment of user reviews of the Skill Academy application, with the aim of identifying how users perceive the quality of online TVET and which algorithm is most suitable for accurately extracting these insights.

This research has high theoretical and practical significance for multiple stakeholders in the digital TVET education ecosystem. For developers of the Skill Academy application and similar TVET platforms, the results of this study provide an empirical data-based understanding of how actual users perceive the quality of online learning, which can be used to optimize user experience, improve the quality of TVET course content, identify specific areas that need improvement, and develop product development strategies that are more responsive to the actual needs of users. For machine learning practitioners, data scientists, and researchers, this study provides valuable empirical benchmarks on the performance of six standard algorithms in the domain of sentiment analysis for TVET learning applications. These findings can isnform algorithm selection in similar sentiment analysis projects in the education domain, improve understanding of the trade-off between model complexity and interpretability, and contribute to the body of knowledge on sentiment classification for e-learning and mobile education platforms. For policymakers, educators, and TVET stakeholders, these findings provide evidence-based understanding of how Indonesian users perceive the quality of online TVET learning platforms, which can support the development of more effective digital transformation strategies for the TVET sector, help policymakers understand user needs and expectations for technology-based vocational learning, and contribute to improving the overall quality of vocational education services in the digital age.

## 2. Method

### A. Data collection and acquisition

The data acquisition process in this study was conducted automatically using the google-play-scraper library version 10.0.1, which enables the retrieval of user reviews directly from the Google Play Store without manual intervention. The target application for data collection was Skill Academy, identified by the application ID com.skillacademy.mobile. Data collection was specifically configured by setting the language and country parameters to Indonesia (lang = “id”, country = “id”), ensuring that all collected reviews originated from Indonesian users. Reviews were sorted using Sort.MOST_RELEVANT to ensure the highest relevance of the retrieved data. A total of 3,000 reviews were successfully collected and stored in CSV format with UTF-8 encoding to preserve Indonesian language characters during subsequent processing stages.

**Figure 1.**
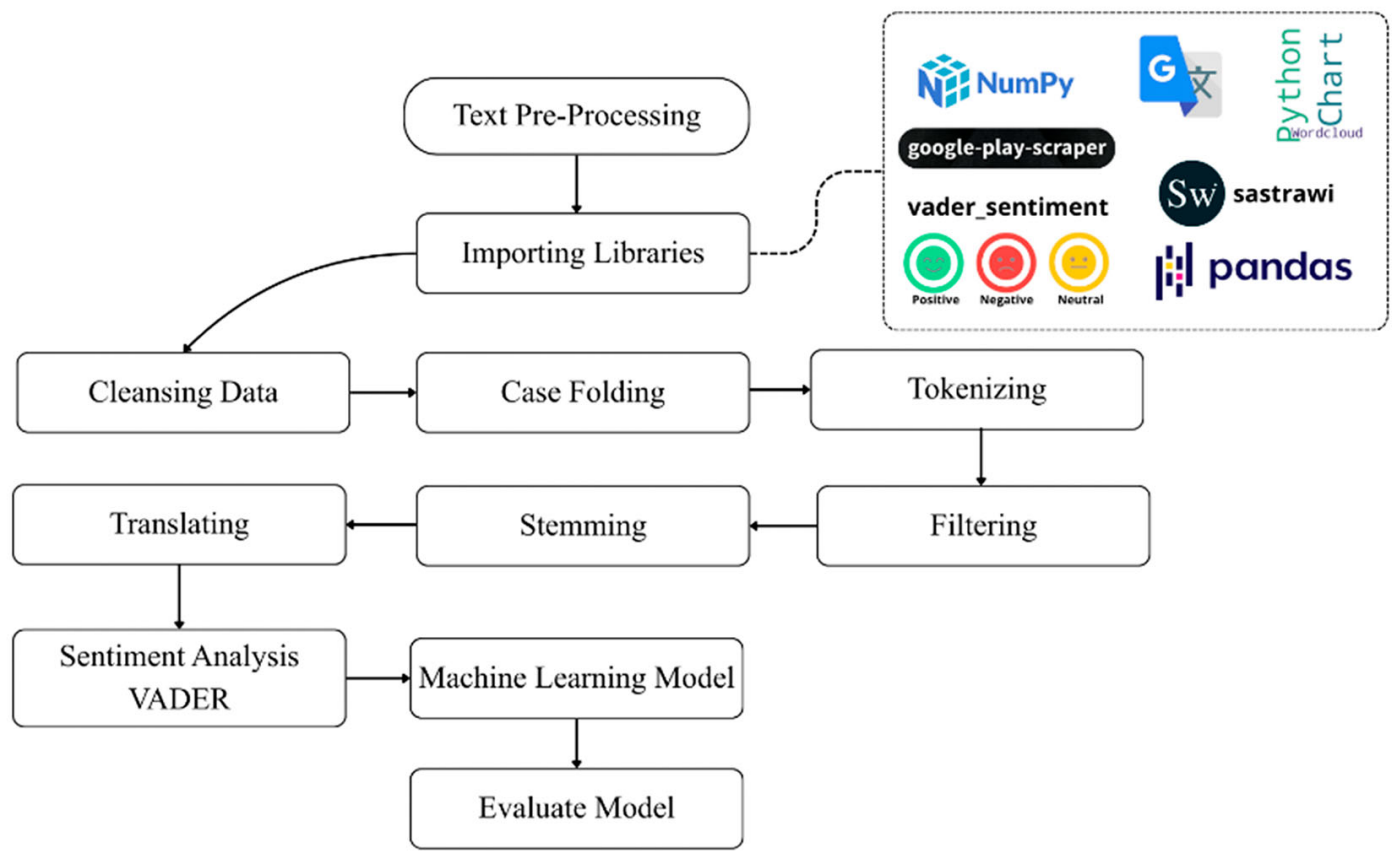
Flowchart of text preprocessing.

Each collected review contains four primary attributes relevant to sentiment analysis, namely the user name (userName), review text content (content), star rating score (score, ranging from 1 to 5), and review submission time (timestamp). These attributes provide comprehensive analytical dimensions, including user identification, textual opinions that serve as the primary input for sentiment analysis, and quantitative indicators in the form of star ratings that can be correlated with sentiment polarity. The collected raw data were subsequently stored in CSV format with UTF-8 encoding to facilitate further text processing while ensuring the integrity of Indonesian language characters. The overall data processing workflow is systematically illustrated in Figure 1.

Figure 1 illustrates the complete system workflow from data acquisition through model evaluation. The process begins with a text preprocessing stage supported by several core libraries, including NumPy for numerical computation, google-play-scraper for data extraction, vader_sentiment for sentiment labeling, Sastrawi for Indonesian language stemming, and Pandas for data management and manipulation. After the required libraries are imported, the raw data undergo a series of preprocessing steps in sequence, including data cleansing, case folding, tokenization, filtering, stemming, and translation. The fully preprocessed text is then analyzed using VADER sentiment analysis to generate positive, negative, and neutral sentiment labels. These sentiment labels subsequently serve as inputs for the machine learning models, which are trained and evaluated during the model evaluation stage. This entire pipeline was designed to ensure consistency and reproducibility of the analytical process, from raw data acquisition to the final classification results.

### B. Preprocessing data

**Figure 2.**
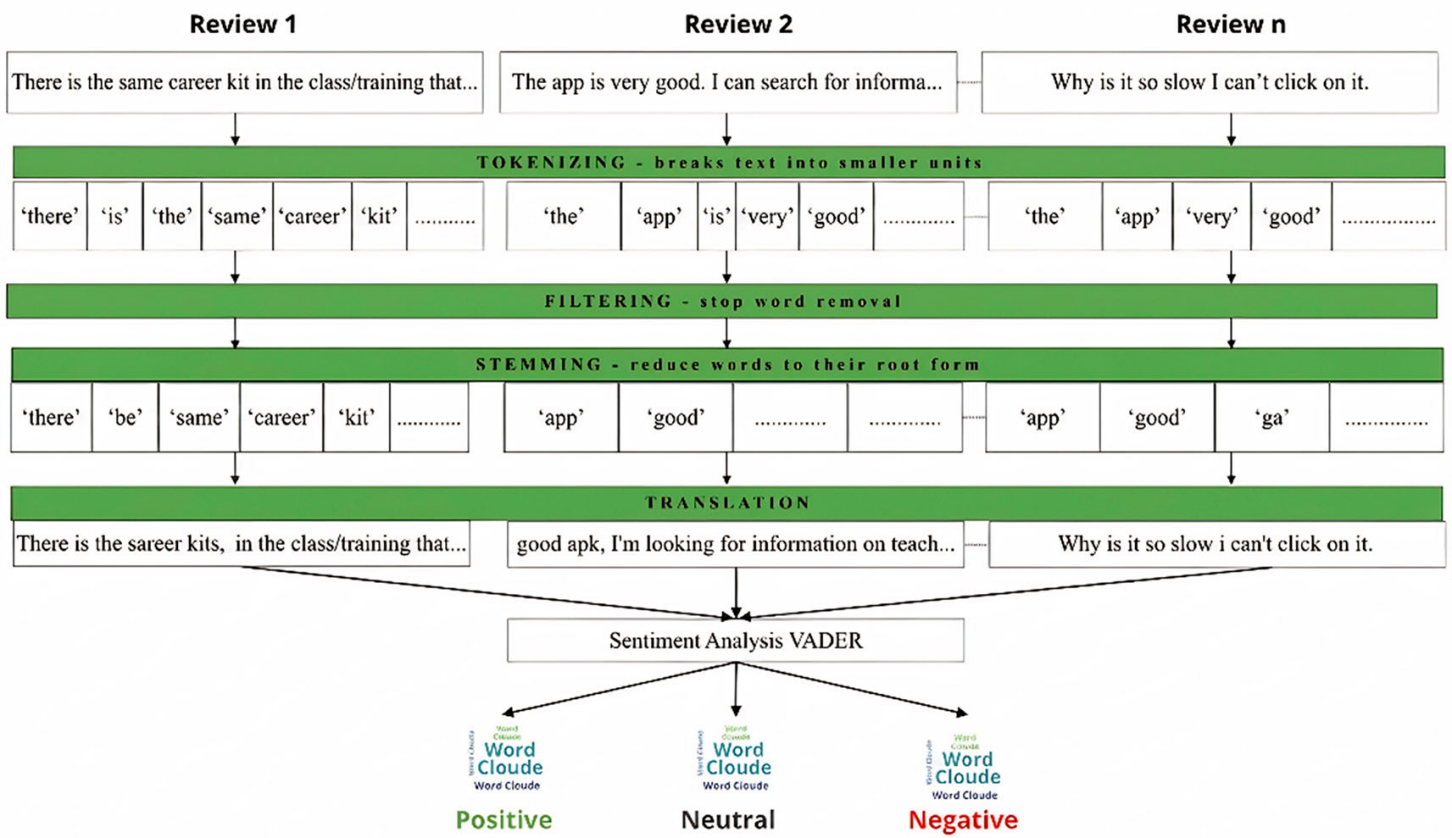
Text preprocessing steps.

Text preprocessing is a stage to ensure data quality and model accuracy. This study implements five preprocessing stages sequentially, as performed by Dias et al ^50^ which is visualized in Figure 2.

1) *Cleansing Data*

The first stage of preprocessing involves removing irrelevant characters from the text data. This includes removing links (URL), user mentions, hashtags, special characters (?, $,.,!, ^, _, “,), (, −, +,,), and emojis converted to ASCII characters. This process is performed by matching regular expression patterns (re.sub) to ensure consistency and efficiency in cleaning 3000 reviews.

2) *Case Folding*

All text is converted to lowercase and white space normalization is performed. This step ensures that identical words with different capitalization (e.g., “Good” and “good”) are treated as the same token, thereby improving the consistency of the analysis.

3) *Tokenizing*

The cleaned text is separated into individual word units (tokens) based on spaces. The application uses a simple separation method with filtering to remove empty tokens. The output of this stage is a list of individual words that are ready for the next processing stage.

4) *Filtering*

Meaningless words are common words that are considered to make no significant contribution to sentiment analysis. This study applies comprehensive meaningless word removal using a combination of three sources: (1) Indonesian meaningless words from NLTK (Natural Language Toolkit), (2) meaningless words from the Sastrawi library (specific to Indonesian), and (3) 13 additional meaningless words specific to the context of mobile application reviews (via, nya, dr, sy, q, pd, &, di, min, sih, ny, aj,,). The total number of meaningless words used is a combination of the three sources with duplication removed to avoid repetition. The use of multiple lists of meaningless words ensures the complete removal of meaningless words while retaining words that carry important sentiment.

5) *Stemming*

Stemming is the process of reducing inflected and derived words to their base or root form. ^51^ This study uses Sastrawi StemmerFactory, which is specifically designed for Indonesian, enabling accurate handling of Indonesian morphology. Each word in the token list is stemmed, so that variations of the same word (e.g., “latihan”, ‘melatih’, “dilatih”) are converted to the same root form, improving feature consistency and reducing dimensionality. The output of the stemming stage is normalized text stored in the ‘clear’ column of the dataset.

6) *Translating*

The stemmed text in Indonesian was translated into English using the googletrans library version 3.1.0a0. The translation was performed with the source language parameter = ‘id’ (Indonesian) and the target language parameter = ‘en’ (English). The translation process with error handling was applied to address cases where translation failed, with the translation results stored in the ‘translated’ column. The use of translation facilitates compatibility with the VADER Sentiment Analyzer, which is optimized for English text and enables more robust analysis of linguistic variation.

7) *VADER*

After text preprocessing is complete, the translated reviews are classified into sentiment categories using the VADER (Valence Aware Dictionary and sEntiment Reasoner) Sentiment Analyzer ^52, 53^ . VADER was chosen for its superiority in analyzing sentiment in informal text, including emoticons, capital letters, and punctuation marks that often appear in mobile app reviews. ^54, 55^ The VADER Sentiment Analyzer calculates a compound score for each translated review. The Compound Score value is determined based on equation 1.

$$C=\frac{\sum_{i=1}^{n}s_{i}}{\sqrt{\sum_{i=1}^{n}s_{i}^{2}+\alpha^{2}}}$$ (1)

*C =* compound score.

S *_i_* = denotation of valence score of the *i*-th token.

α = normalization constant.

*n* = total number of token in text.

The core of decision-making in VADER is the composite score, which is a combined score calculated by summing the valence scores of each token in the text, then normalizing that sum within the range of −1 to +1. ^56^ Hernández-Pérez R 2024 classifying sentiment using three thresholds: ^57^

• Positive: combined score ≥ 0.05
• Negative: combined score ≤ −0.05
• Neutral: −0.05 < combined score < 0.05

A threshold of 0.05 was chosen to provide a reasonable margin in separating positive and negative sentiment from neutral sentiment, allowing for a more detailed classification of user opinions.

### C. Feature extraction and train-test split

After sentiment labeling using VADER, the next step is to convert the preprocessed text into numerical feature vectors that can be processed by machine learning algorithms.

1) *TF-IDF Encoding and Vectorization*

Categorical sentiment labels (Negative, Neutral, Positive) are converted into numerical labels using sklearn Label Encoder, resulting in the mapping: Negative = 0, Neutral = 1, Positive = 2 (based on alphabetical order). Preprocessed text from the ‘clear’ column is converted into feature vectors using TF-IDF (Term Frequency-Inverse Document Frequency) vectorization. The vectorizer is configured with a maximum number of features = 5000 to limit the number of features and prevent overfitting, retaining the 5000 features with the highest TF-IDF scores from the entire dataset vocabulary. The vectorization output is a dense array of dimensions 3000 × 5000 (3000 reviews × 5000 features).

2) *Splitting Data*

The vectorized dataset was split into training and testing sets with a 70:30 ratio using the sklearn train-test splitting function. The split was performed with a random seed (random_state) = 42 to ensure that the results could be processed. This configuration resulted in a training set with 2100 samples and a testing set with 900 samples.

### D. Visualization

Visualizations were generated to understand the characteristics of the data and the performance of the model in classifying the sentiment of Skill Academy user reviews. First, the bar chart displays the absolute distribution and percentage of the three sentiment categories resulting from VADER labeling, while the histogram shows the continuous distribution of compound scores to provide insight into the spread of sentiment in the dataset. ^58^ Word frequency analysis in reviews is displayed through horizontal bar charts showing the 20 most frequently occurring words and their contribution percentages, and separate word cloud visualizations are generated for each sentiment category (Positive, Neutral, Negative) to identify specific linguistic patterns that characterize each sentiment. ^59, 60^

### E. Modelling performance

This study uses six machine learning algorithms to analyze the sentiment of user reviews of the Skill Academy application: Naive Bayes, Support Vector Machine (SVM), Logistic Regression, Random Forest, Decision Tree, and K-Nearest Neighbors (KNN). Each algorithm has its own characteristics and advantages in sentiment classification based on review data. Model performance is evaluated using:

3) *Confusion Matrix*

It is an evaluation tool used to measure the performance of classification models, especially in sentiment classification problems such as positive, negative, and neutral. This matrix provides a clear picture of how well the model predicts the correct category and, conversely, predicts the wrong category. The confusion matrix consists of three target classes (positive, negative, neutral) which can be seen in Table I.

**Table I.** Confusion matrix evaluation.

	Positive Prediction	Negative Prediction	Neutral Prediction
**Actual Positive**	True Positives (TP)	False Negatives (FN)	False Negatives (FN)
**Actual Negative**	False Positives (FP)	True Negatives (TN)	False Negatives (FN)
**Actual Neutral**	False Positives (FP)	False Positives (FP)	True Negatives (TN)

The evaluation metrics for calculating the confusion matrix are as follows:

a. Accuracy: Measuring the proportion of correct predictions against the total predictions made.
$$\text{Accuracy}=\frac{\mathrm{TP}+\mathrm{TN}}{\mathrm{TP}+\mathrm{TN}+\mathrm{FP}+\mathrm{FN}}$$ (2)
b. Precision: Measuring how many positive predictions are actually positive.
$$\text{Precision}=\frac{\mathrm{TP}}{\mathrm{TP}+\mathrm{FP}}\;$$ (3)
c. Recall: Measuring how many positive cases are actually predicted as positive.
$$\text{Recall}=\frac{\mathrm{TP}}{\mathrm{TP}+\mathrm{FN}}$$ (4)
d. F1-Score: Measuring the model’s ability to correctly identify negative cases.
$$F1-\text{Score}=2\times \frac{\text{Precision}\times \text{Recall}}{\text{Precision}+\text{Recall}}$$ (5)

4) *ROC-AUC (Receiver Operating Characteristic - Area Under Curve)*

ROC-AUC is an evaluation metric used to measure the performance of a classification model in separating positive and negative classes. ROC (Receiver Operating Characteristic) is a curve that describes the trade-off between True Positive Rate (TPR) and False Positive Rate (FPR) at various classification threshold values. Meanwhile, AUC (Area Under Curve) measures the area under the ROC curve, which gives a value between 0 and 1.

a. True Positive Rate (TPR), often referred to as Recall or Sensitivity, measures how many of the true positive data are correctly predicted as positive by the model.
$$\mathrm{TPR}=\frac{\mathrm{TP}}{\mathrm{TP}+\mathrm{FN}}$$ (6)
b. The False Positive Rate (FPR) measures how much false negative data is predicted as positive by the model.
$$\mathrm{FPR}=\frac{\mathrm{FP}}{\mathrm{FP}+\mathrm{TN}}$$ (7)
c. AUC (Area Under Curve) measures the area under the ROC curve, which illustrates the model’s ability to separate positive and negative classes at various classification thresholds. AUC provides a clear picture of the model’s performance. To evaluate model performance based on AUC, the following categories are shown in Table II. ^61^

**Table II.** AUC Score classification.

AUC Score	Category
0.90–1.00	Excellent classification
0.80–0.90	Good classification
0.70–0.80	Fair classification
0.60–0.70	Poor classification
0.50–0.60	Failure classification

The AUC (Area Under Curve) value provides a clear picture of the model’s performance in distinguishing between positive and negative classes. If the AUC is in the range of 0.90–1.00, then the model falls into the Excellent classification category, which indicates that the model is very good at distinguishing classes with high precision in almost all cases. In the range of 0.80–0.90, the model is categorized as Good classification, which means that the model is reliable, although there is still room for minor improvements. If the AUC is in the range of 0.70–0.80, the model is considered Fair classification, which indicates that the model is quite good but there are still significant errors in distinguishing between positive and negative classes. If the AUC is in the range of 0.60–0.70, the model falls into the Poor classification category, with limited ability to distinguish classes and requiring significant improvement to work better. Finally, if the AUC is in the range of 0.50–0.60, the model is categorized as a failure classification, meaning that the model fails to distinguish classes well and is barely better than random guessing, thus requiring major improvements or significant model changes.

## 3. Result and discussion

### A. Data analysis and initial findings

**Figure 3.**
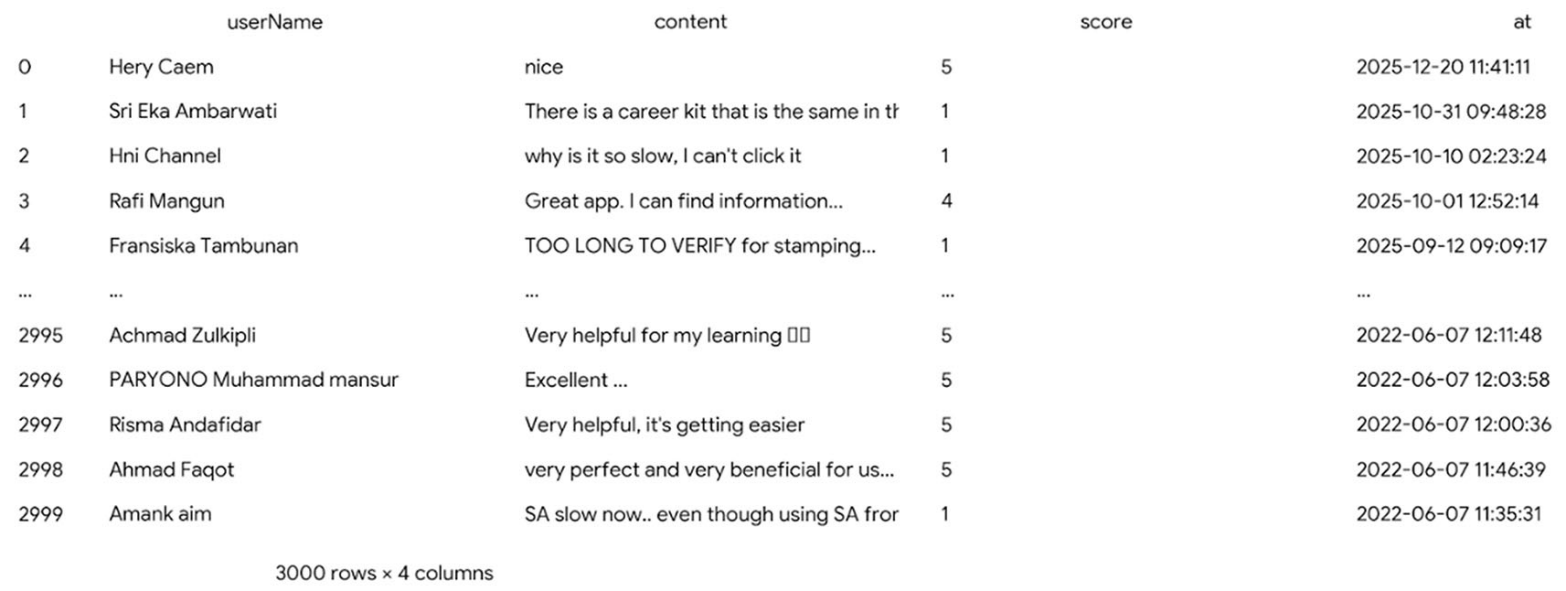
Initial finding results.

The research dataset consists of 3000 user reviews collected from the Google Play Store for the Skill Academy app. Each review contains information on the user name, the original review content text, a star rating (1–5), and the submission time. Figure 3 shows reviews with the preprocessing steps that have been applied, showing the text transformation from its original form to a form ready for sentiment analysis.

Initial observations from the data show that Skill Academy user reviews are generally descriptive and specific. Positive reviews tend to highlight the ease of use of the application, the quality of the training content, and the perceived benefits of learning. Conversely, negative reviews consistently focus on technical issues such as application bugs, slow loading speeds, and the need for certain feature updates. This pattern shows that users have clear expectations about what they expect from an online vocational training platform.

**Figure 4.**
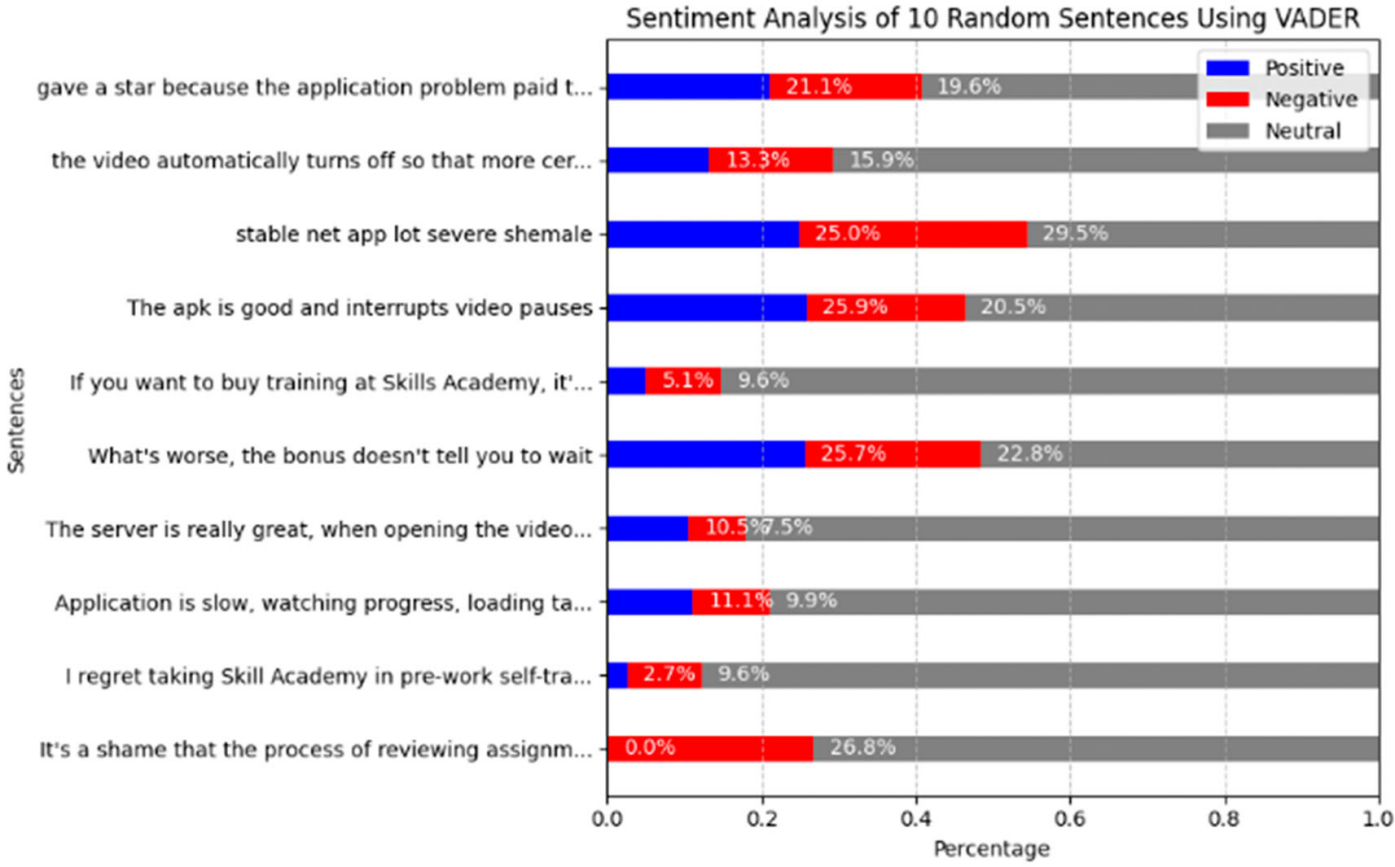
Basic sentiment analysis using VADER.

The results of sentiment analysis on 10 randomly selected sentences analyzed using VADER Sentiment Analysis are shown in Figure 4. Each bar shows the proportion of Positive, Negative, and Neutral sentiments for each sentence. From these results, it can be seen that some sentences, such as “The server is really great, when opening the video…”, have a predominance of Positive sentiment, reflecting user satisfaction with the application. Meanwhile, sentences complaining about technical issues, such as “Application is slow, watching progress, loading…”, tend to have Negative sentiment, illustrating disappointment with the application’s performance. There are also more moderate sentences with a Neutral distribution, indicating that some users feel neither particularly positive nor negative about the application. This indicates that VADER is able to classify sentiment well, even though there are some more ambiguous sentences, resulting in a significant proportion of Neutral sentiment. Overall, this model successfully distinguishes sentiment quite well, even though Neutral sentiment still dominates, reflecting that many users provide more balanced and less extreme feedback.

**Figure 5.**
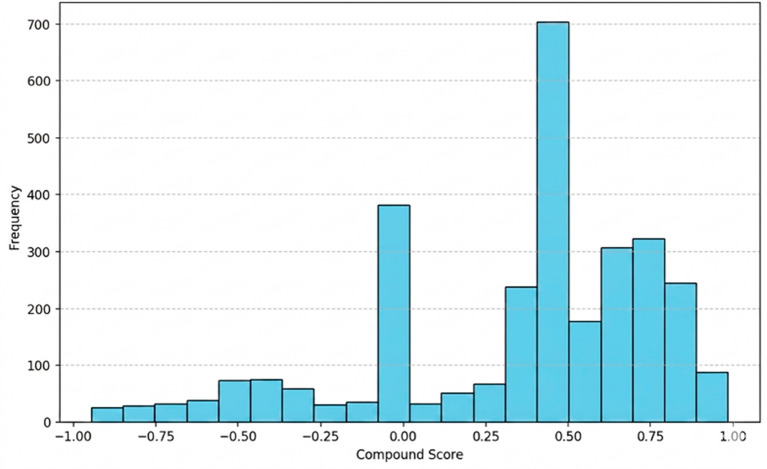
Compound score.

The distribution of Compound Sentiment Score in this histogram provides an overview of how strong the sentiment contained in user reviews is, with scores ranging from −1 (very negative) to +1 (very positive). From the results shown in Figure 5, the majority of reviews have scores close to 0, indicating that many user reviews are neutral, with little or no extreme emotions related to their experience with the app. This indicates that most users may feel indifferent or do not have strong feelings about the app. However, there are also a number of reviews with positive scores (0.25 to 0.75), indicating that some users are satisfied and like the app, especially in terms of ease of use and content quality. On the other hand, there are also reviews with negative scores (−0.25 to −0.75), reflecting dissatisfaction or complaints about technical issues such as bugs or poor app performance. Overall, this distribution shows that although most reviews tend to be moderate or neutral, there is a significant proportion of reviews that reflect both positive and negative experiences, providing a more complete picture of users’ perceptions of the app.

### B. Sentiment distribution

**Figure 6.**
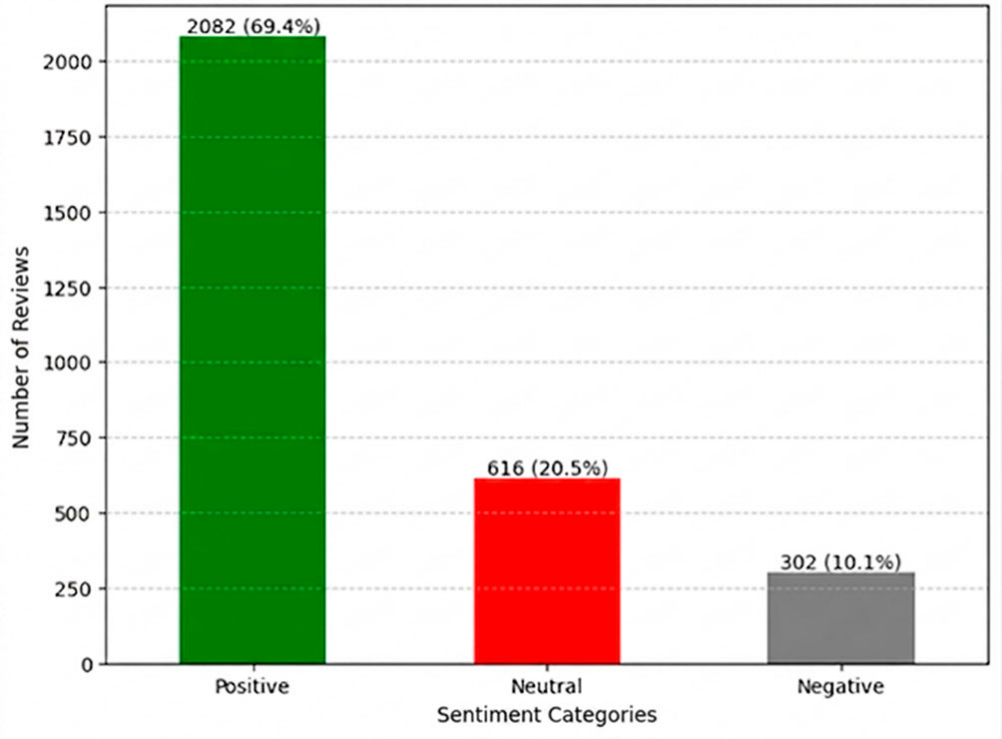
Sentiment count distribution.

This graph in Figure 6 shows the distribution of user sentiment based on sentiment analysis of the Skill Academy app review dataset. From the graph, it can be seen that the majority of user reviews are in the Positive category, with a total of 2082 reviews (69.4%). This shows that most users are satisfied or have a positive experience with the app, possibly related to the quality of content, ease of use, or benefits gained from the app. On the other hand, the Neutral category includes 616 reviews (20.5%), which shows that a group of users feel indifferent or do not have a strong opinion about the app. These reviews may reflect less remarkable experiences or simply provide more general feedback without clear emotions. Meanwhile, the Negative category only includes 302 reviews (10.1%), indicating that although there are a number of complaints or dissatisfaction with the app, the number is relatively smaller compared to positive reviews. These complaints may focus on technical issues such as app bugs, speed issues, or inadequate features.

### C. Top 20 Most frequently used words

**Figure 7.**
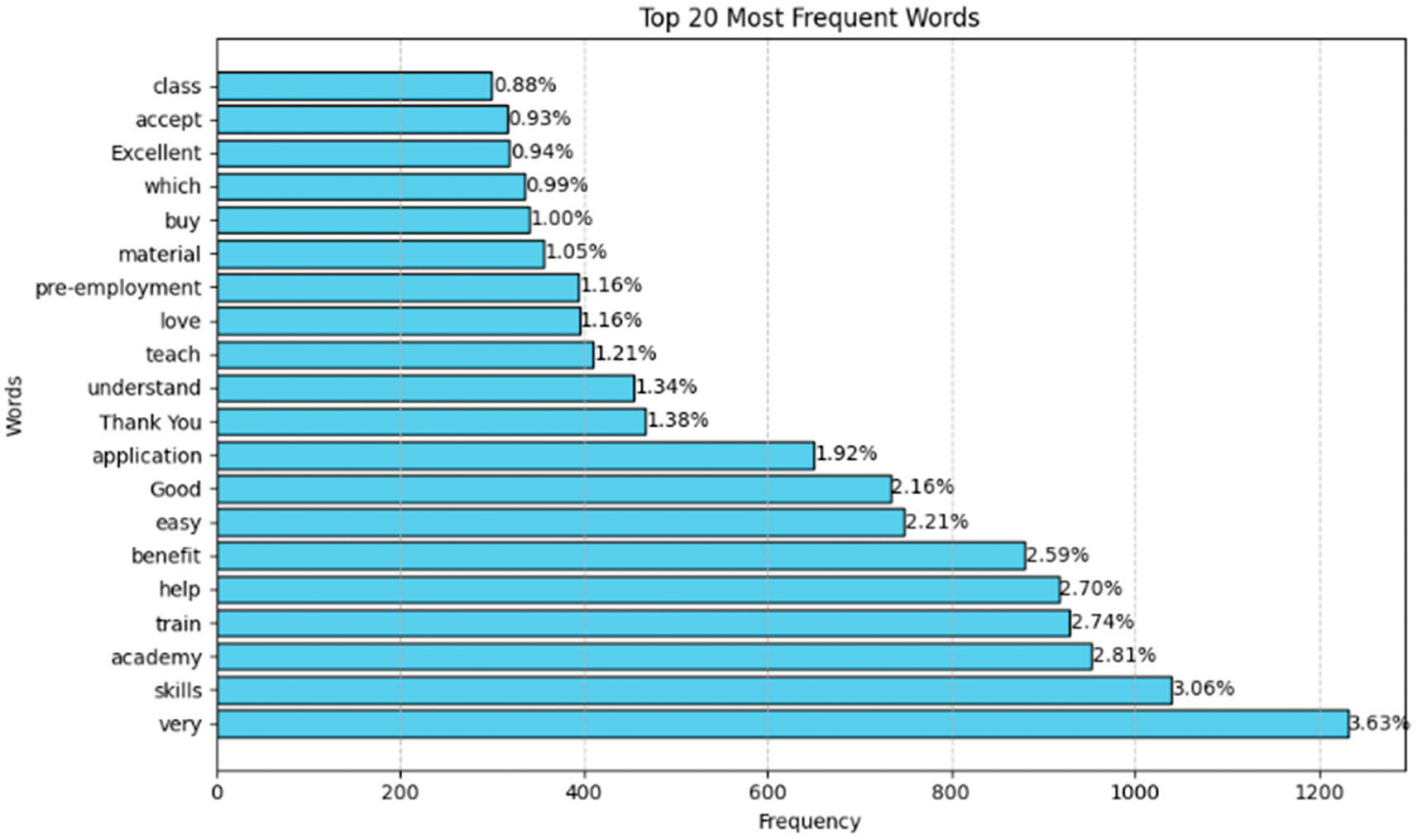
Top 20 Most Frequently Used Words.

This graph in Figure 7 shows the words that appear most frequently in the reviews analyzed, providing an overview of the main themes often discussed by respondents. From these results, we can identify words that reflect users’ keen interest in the quality of training materials, their experiences, and elements considered important in the programs they participated in.

The word “very” appeared with the highest frequency (6.63%), indicating that many respondents used this word to emphasize the intensity or strength of their opinions, especially when giving praise or positive assessments. The word “skills” (3.06%) appears very frequently, indicating that respondents are very focused on the skills they have acquired from the program or training they have participated in, which may be the main reason they joined the program. “Academy” (3.06%) shows that many users refer to the institution or organization where the training was held, illustrating the relevance of the institution or program organizer in the reviews provided.

The word “excellent” (2.92%) indicates that many reviews noted the program’s excellent quality, followed by “good” (1.92%), which emphasizes good quality but is more common. The word “easy” (2.70%) indicates that participants found the program easy to follow, and “help” (2.59%) highlights the assistance provided during training, showing satisfaction with the support they received. In addition, the word “pre-employment” (1.16%) appeared, reflecting that many reviews focused on preparation for work or programs aimed at helping participants gain skills relevant to the world of work. This shows that many participants took part in these programs as part of their preparation for entering the world of work or improving their qualifications for better jobs. Words such as “train” (2.74%), ‘benefit’ (2.59%), and “application” (1.38%) also appear, describing the practical benefits felt by participants, including skills that can be directly applied in a professional context.

Overall, the words that appear most frequently in these reviews describe a generally positive experience with the quality of the materials and training provided, with a strong focus on developing skills that can be applied in the workplace, as well as preparation for employment through pre-employment programs. This indicates that many participants feel that this training is beneficial for their career advancement. In addition, the most frequently used words describe a predominantly positive experience, especially in relation to skills development and satisfaction with the quality of the materials or training. This reflects that the majority of reviews tend to provide positive feedback on the programs or classes offered.

### D. Word cloud

This Word Cloud displays the words that appear most frequently in user reviews of the Skill Academy app. The words “good” and “Skill Academy” appear as the largest words, indicating that many users express their positive experiences with this app, often highlighting the quality and benefits they gain from the training. Words such as “helpful,” “easy,” and “training” show that users appreciate the app’s ease of use and the benefits they gain from skills training. In addition, “pre_employment” and ‘class’ indicate that many users are using the app for job preparation or further education. The word “thank” also reflects users’ appreciation for this app. Meanwhile, words such as “benefit,” “material,” and “application” indicate that users often comment on the features and quality of the materials provided. Overall, this word cloud reflects the positive experiences of users who find the app useful for improving their skills, with a focus on training and job preparation.

**Figure 8.**
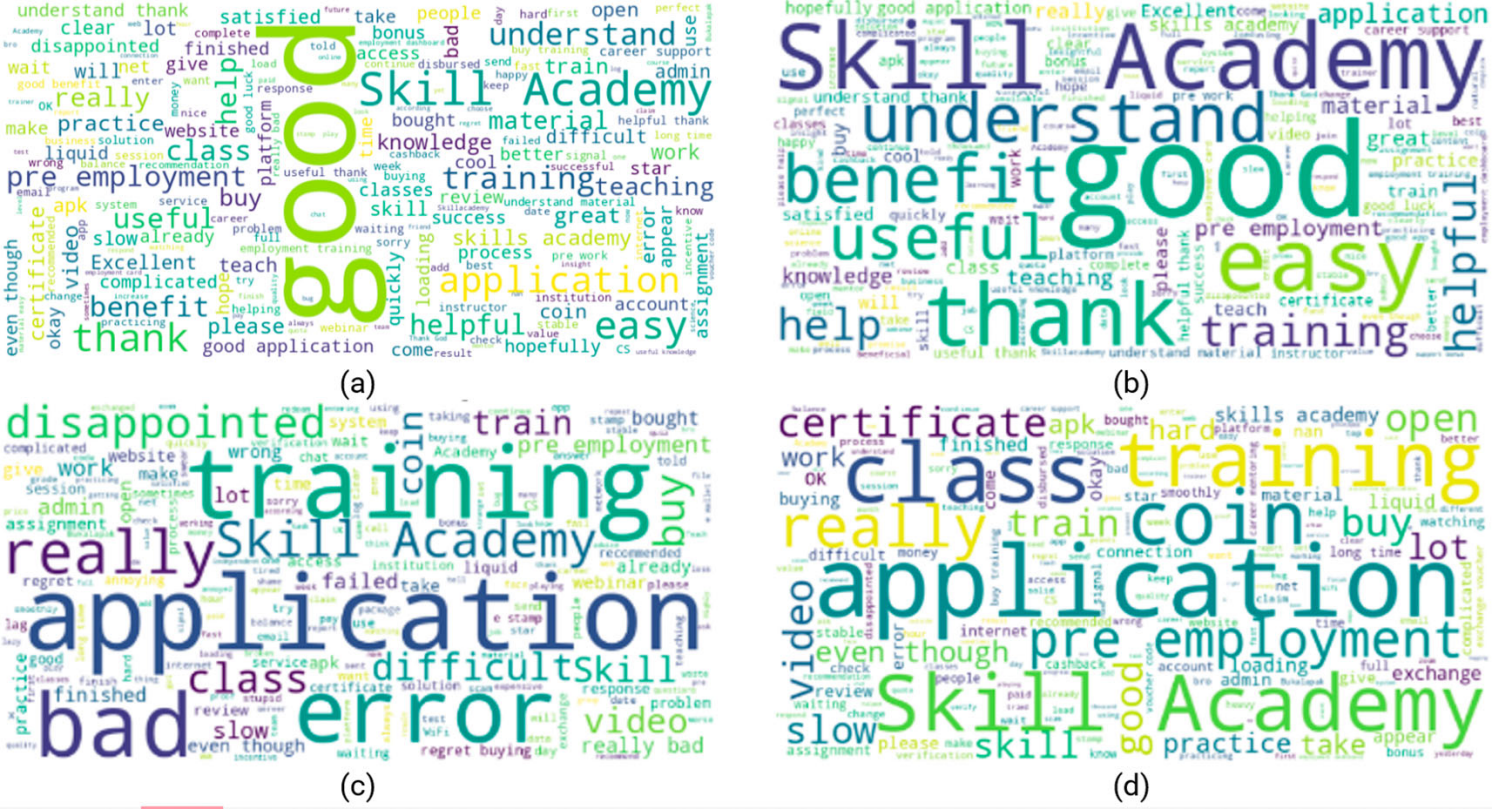
Wordcloud; (a) All keyword wordcloud; (b) Positive wordcloud; (c) Negative wordcloud; (d) Neutral wordcloud.

There are words with positive sentiment show in Figure 8b, such as “Skill Academy” and “Good,” which appear most frequently, indicating that many positive reviews are related to the quality of Skill Academy as a training institution and the good experiences received by participants. The word “Thank” also appears quite frequently, indicating the gratitude often expressed by participants who are satisfied with the programs or training they have attended. In addition, the words “Benefit” and ‘Useful’ show that participants feel the practical benefits of the training, and they feel that the program provides added value for their skills development. “Easy” also appears frequently, indicating that many participants feel that the training material is easy to understand and follow. Overall, these words reflect generally positive reviews that focus on satisfaction with the quality of the program.

In negative sentiment (see Figure 8c), the words “Application” and “Error” appear very dominantly, indicating that many negative reviews are related to technical issues encountered by participants, such as errors in the application or difficulties in accessing training materials. The words ‘Bad’ and “Disappointed” show the disappointment felt by participants regarding their experience, which is most likely caused by technical errors or training that did not meet expectations. In addition, the word “Training” appears quite frequently, indicating that although the training itself exists, many participants feel that the program is ineffective or difficult to follow. Negative reviews more often reflect frustration with technical quality and failure to meet user expectations.

For neutral sentiment in Figure 8d, the words “Application” and ‘Training’ are also dominant, but in a more neutral context, indicating that participants have mixed opinions or are unclear about the training. The word “Class” appears quite frequently, indicating that many participants provided reviews related to their classroom experience, but without overly positive or negative assessments. In addition, the words “Certificate” and “Pre-employment” show that participants view this training as part of their job preparation, but these reviews highlight a more passive or less emotional experience, tending to describe a more objective view of the quality of the program.

### E. Machine learning evaluation

In testing the Naive Bayes model (see Table III), an accuracy of 0.73 was obtained, indicating that this model is capable of correctly predicting approximately 73% of the total test data. Based on the precision, recall, and f1-score values for each category, it can be seen that the model’s performance varies between classes. For the Negative category, the model showed very high precision (0.88), indicating that the model often correctly predicted negative data. However, the low recall (0.07) indicated that the model failed to detect most of the negative data, resulting in many false negatives. Meanwhile, for the Neutral category, the precision (0.46) and recall (0.26) are relatively low, indicating that the model is less effective in identifying neutral data. In the Positive category, the model provides the best results with a precision of 0.77, a recall of 0.98, and an f1-score of 0.86, which indicates that the model is very effective in recognizing and predicting positive data.

**Table III.** Naïve bayes result.

Parameter	Precision	Recall	F1-score	Support
Negative	0.88	0.07	0.13	101
Neutral	0.46	0.26	0.33	180
Positive	0.77	0.98	0.86	619
Accuracy	0.7322222222222222			900
Macro avg	0.70	0.44	0.44	900
Weightes avg	0.72	0.73	0.67	900

**Figure 9.**
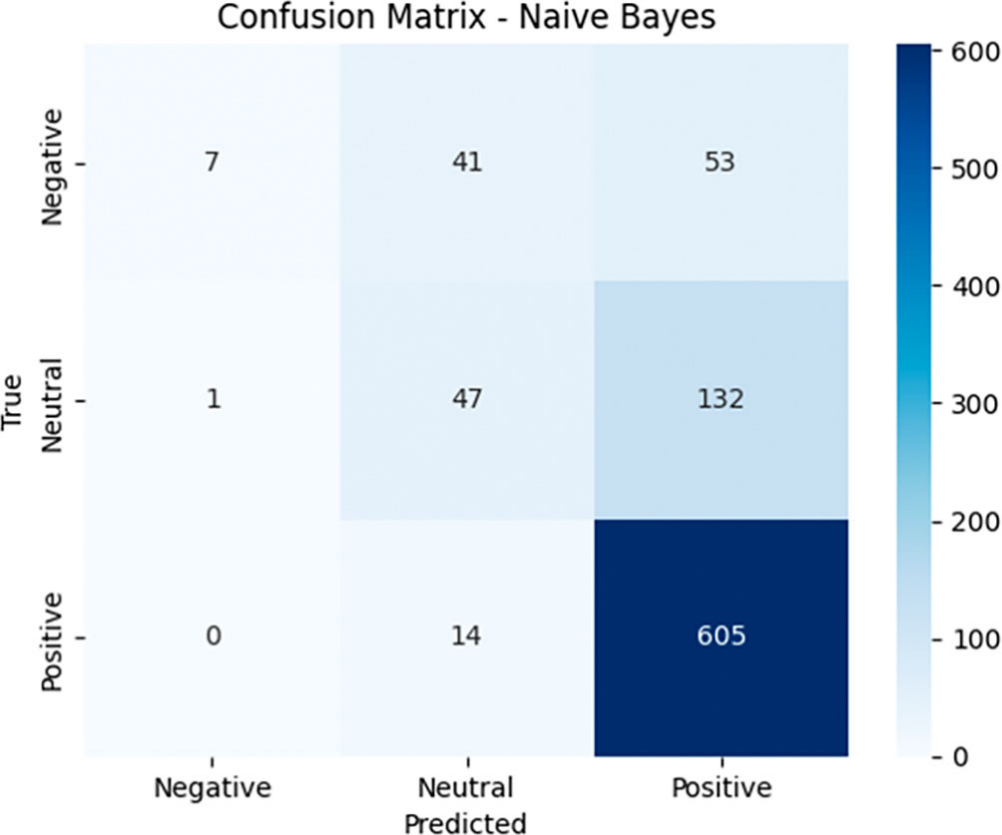
Naive bayes confusion matrix.

In the Confusion Matrix generated by the Naive Bayes model (see Figure 9), we can observe the distribution of the model’s predictions as follows. For the Negative category, the model successfully identified 7 data points as Negative (true negative), but there were 41 data points that were actually Negative but predicted as Neutral (false positive) and 53 data points that were predicted as Positive (false positive). For the Neutral category, the model correctly predicted 47 data points as Neutral (true positive), but there was 1 data point that should have been Neutral but was predicted as Negative (false negative) and 132 data points that were incorrectly predicted as Positive (false negative). Meanwhile, in the Positive category, the model successfully classified 605 data correctly as Positive (true positive), but there were 14 data that should have been Positive but were predicted as Neutral (false negative). Although Naive Bayes performed very well in predicting the Positive category with a high true positive value, this model still had difficulty distinguishing between Neutral and Positive, as seen in the large number of false negatives and false positives in both categories. In addition, Negative was also often mispredicted as Neutral and Positive, indicating that although the model’s accuracy was quite good, the separation between classes was not optimal, especially in the Neutral and Negative classes.

The tested Support Vector Machine (SVM) model produced an accuracy of 0.86 in Table IV, indicating that the model successfully predicted approximately 86% of the total test data correctly. Based on the precision, recall, and f1-score values for each category, the model’s performance varied between classes. For the Negative category, a precision of 0.84 indicates that the model was able to correctly predict approximately 84% of all predictions made for the negative class, but a lower recall (0.53) indicates that only 53% of the negative data was actually detected by the model. This means that the model fails to detect much of the existing negative data, resulting in many false negatives. In the Neutral category, a precision of 0.63 and a recall of 0.81 indicate that although the model tends to frequently mispredict neutral data as positive (due to high recall), precision can still be improved to reduce incorrect predictions. Meanwhile, in the Positive category, the model shows the best performance with a precision of 0.94, a recall of 0.92, and an f1-score of 0.93, which indicates that the model is very effective in recognizing and predicting positive data.

**Table IV.** SVM result.

Parameter	Precision	Recall	F1-score	Support
Negative	0.84	0.53	0.65	101
Neutral	0.63	0.81	0.71	180
Positive	0.94	0.92	0.93	619
Accuracy	0.8577777777777778			900
Macro avg	0.81	0.76	0.77	900
Weights avg	0.87	0.86	0.86	900

**Figure 10.**
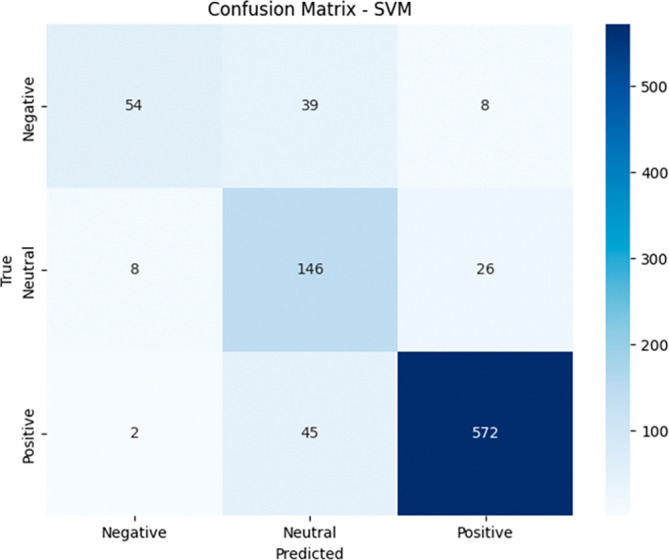
SVM confusion matrix.

In the resulting Confusion Matrix in Figure 10, it can be seen that the SVM model successfully identified 54 data points as Negative (true negative), but there were 39 data points that should have been Negative but were predicted as Neutral (false positive), and 8 data points that should have been Negative but were predicted as Positive (false positive). For the Neutral category, the model correctly predicted 146 data points as Neutral (true positive), but also classified 8 Neutral data points as Negative (false negative) and 26 data points as Positive (false negative). As for the Positive category, the model correctly predicted 572 data points (true positive), although there were 2 Positive data points predicted as Negative (false negative) and 45 data points predicted as Neutral (false negative). These results show that although the model is very good at detecting Positive data, it still has difficulty distinguishing between Negative and Neutral, with many false positives and false negatives in both categories.

The tested Logistic Regression model produced an accuracy of 0.85 (see Table V), indicating that this model successfully predicted approximately 85% of the total test data correctly. Based on the precision, recall, and f1-score values for each category, the model’s performance varies between classes. For the Negative category, a precision of 0.92 indicates that the model is very accurate in predicting negative data, but a lower recall (0.46) indicates that the model only manages to detect about 46% of the actual negative data, resulting in many false negatives. For the Neutral category, a precision of 0.64 and a recall of 0.75 indicate that the model is quite effective in identifying neutral data, although there is still room for improvement in precision to reduce prediction errors. Meanwhile, in the Positive category, the model shows excellent performance with a precision of 0.91, a recall of 0.94, and an F1-score of 0.93, indicating that the model is highly effective in recognizing and predicting positive data with a very low error rate.

**Table V.** Logistic regression result.

Parameter	Precision	Recall	F1-score	Support
Negative	0.92	0.46	0.61	101
Neutral	0.64	0.75	0.69	180
Positive	0.91	0.94	0.93	619
Accuracy	0.8488888888888889			900
Macro avg	0.82	0.72	0.74	900
Weights avg	0.86	0.85	0.84	900

**Figure 11.**
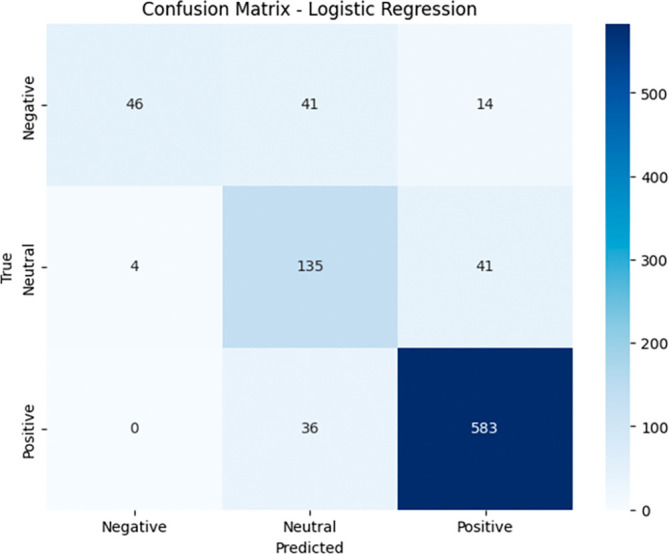
Logistic regression confusion matrix.

In the Confusion Matrix generated by the Logistic Regression model (see Figure 11), the distribution of model predictions can be observed as follows. For the Negative category, the model correctly classified 46 data points as Negative (true negative), but there were 41 Negative data points that were predicted as Neutral (false positive) and 14 data points that were predicted as Positive (false positive). For the Neutral category, the model correctly predicted 135 data points as Neutral (true positive), but there were 4 data points that should have been Neutral but were predicted as Negative (false negative) and 41 data points that were incorrectly predicted as Positive (false negative). As for the Positive category, the model successfully identified 583 data correctly as Positive (true positive), although there were 36 data that should have been Positive but were predicted as Neutral (false negative). These results show that even though the model performs very well in predicting Positive, there are still errors in Negative and Neutral, especially with the large number of false positives and false negatives in both categories. This model is highly effective in recognizing positive data, but improvements are needed for the Negative and Neutral categories to minimize classification errors between classes.

The Random Forest model tested produced an accuracy of 0.84 in Table VI, indicating that this model successfully predicted approximately 84% of the total test data correctly. Based on the precision, recall, and f1-score values for each category, the model’s performance varies between classes. For the Negative category, the model shows high precision (0.89), which means that the model is quite accurate in predicting negative data, but lower recall (0.48) indicates that only 48% of negative data was actually detected, leading to many false negatives. For the Neutral category, the model has a precision of 0.59 and a recall of 0.83, which shows that although the model is quite effective in detecting neutral data (with high recall), there is still room to improve precision to reduce prediction errors. In the Positive category, the model shows the best performance with a precision of 0.93, a recall of 0.90, and an f1-score of 0.92, which shows that the model is very effective in recognizing and predicting positive data with very few errors.

**Table VI.** Random forest result.

Parameter	Precision	Recall	F1-score	Support
Negative	0.89	0.48	0.62	101
Neutral	0.59	0.83	0.69	180
Positive	0.93	0.90	0.92	619
Accuracy	0.8355555555555556			900
Macro avg	0.80	0.73	0.74	900
Weights avg	0.86	0.84	0.84	900

**Figure 12.**
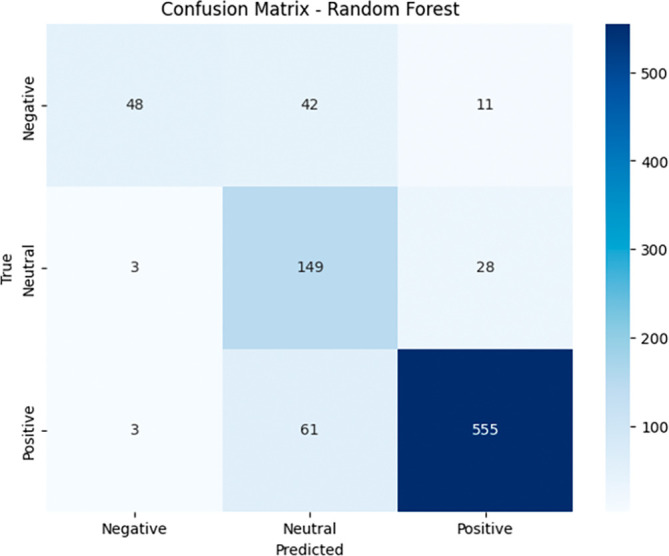
Random forest confusion matrix.

In the Confusion Matrix generated by the Random Forest model (see Figure 12), we can see the distribution of the model’s predictions as follows. For the Negative category, the model successfully identified 48 data points correctly as Negative (true negative), but there were 42 data points that should have been Negative but were predicted as Neutral (false positive) and 11 data points that were predicted as Positive (false positive). For the Neutral category, the model successfully classified 149 data points correctly as Neutral (true positive), but there were 3 data points that should have been Neutral but were predicted as Negative (false negative) and 28 data points that were incorrectly predicted as Positive (false negative). As for the Positive category, the model successfully predicted 555 data correctly as Positive (true positive), although there were 61 data that should have been Positive but were predicted as Neutral (false negative). Overall, although the Random Forest model performed very well in predicting Positive, difficulties still occurred in Negative and Neutral, with quite a number of false positives and false negatives in both categories. The model is very effective in detecting Positive data, but improvements are needed in separating Negative and Neutral, particularly by increasing recall in the Negative category and precision in the Neutral category.

The tested Decision Tree model produced an accuracy of 0.84 in Table VII, indicating that this model successfully predicted approximately 84% of the total test data correctly. Based on the precision, recall, and f1-score values for each category, the model’s performance varies between classes. For the Negative category, the model has a precision of 0.73, which indicates that the model is quite accurate in predicting negative data, but the lower recall (0.59) indicates that only 59% of negative data was actually detected, leading to many false negatives. For the Neutral category, a precision of 0.64 and a recall of 0.74 indicate that the model is effective in detecting neutral data, although there is still room for improvement in precision to reduce prediction errors. Meanwhile, in the Positive category, the model shows the best performance with a precision of 0.93, a recall of 0.91, and an f1-score of 0.92, indicating that the model is very effective in recognizing and predicting positive data with a very low error rate.

**Table VII.** Decision tree result.

Parameter	Precision	Recall	F1-score	Support
Negative	0.73	0.59	0.66	101
Neutral	0.64	0.74	0.69	180
Positive	0.93	0.91	0.92	619
Accuracy	0.8433333333333334			900
Macro avg	0.77	0.75	0.76	900
Weightes avg	0.85	0.84	0.84	900

**Figure 13.**
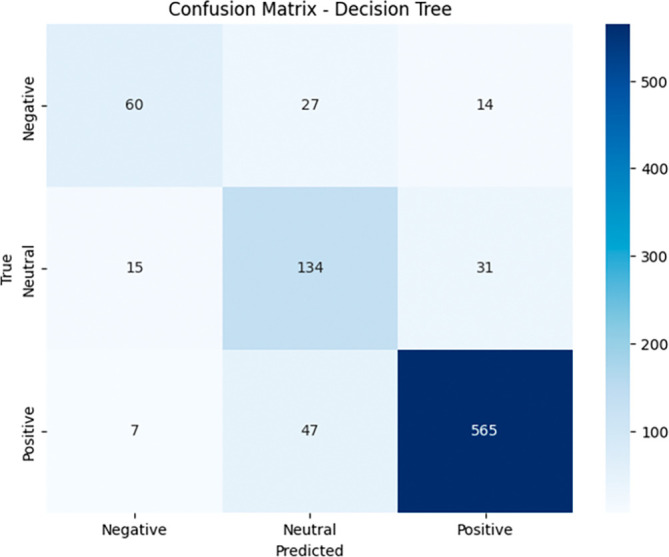
Decision tree confusion matrix.

In the Confusion Matrix generated by the Decision Tree model (see Figure 13), the distribution of model predictions can be observed as follows. For the Negative category, the model successfully identified 60 data points as Negative (true negative), but there were 27 Negative data points that were predicted as Neutral (false positive) and 14 data points that were predicted as Positive (false positive). For the Neutral category, the model successfully classified 134 data points as Neutral correctly (true positive), but there were 15 data points that should have been Neutral but were predicted as Negative (false negative) and 31 data points that were incorrectly predicted as Positive (false negative). As for the Positive category, the model successfully identified 565 data as Positive (true positive), although there were 7 data that should have been Positive but were predicted as Negative (false negative) and 47 data that were predicted as Neutral (false negative).

The K-Nearest Neighbors (KNN) model tested produced an accuracy of 0.66 in Table VIII, indicating that this model successfully predicted approximately 66% of the total test data correctly. Based on the precision, recall, and f1-score values for each category, the model’s performance varied between classes. For the Negative category, a precision of 0.75 indicates that when the model predicted data as Negative, 75% of those predictions were correct. However, the very low recall (0.06) indicates that the model only managed to detect 6% of the actual Negative data, resulting in many false negatives. In the Neutral category, a precision of 0.36 and a recall of 0.94 indicate that although the model is very good at detecting Neutral data (with high recall), its precision is very low, which means that many Neutral predictions are incorrect, especially predictions that should be Positive. In the Positive category, the model performed quite well with a precision of 0.98 and a recall of 0.67, indicating that the model is effective at predicting positive data, although there are some errors in detecting Positive as Neutral.

**Table VIII.** KNN Result.

Parameter	Precision	Recall	F1-score	Support
Negative	0.75	0.06	0.11	101
Neutral	0.36	0.94	0.52	180
Positive	0.98	0.67	0.80	619
Accuracy	0.6566666666666666			900
Macro avg	0.70	0.56	0.48	900
Weights avg	0.83	0.66	0.66	900

**Figure 14.**
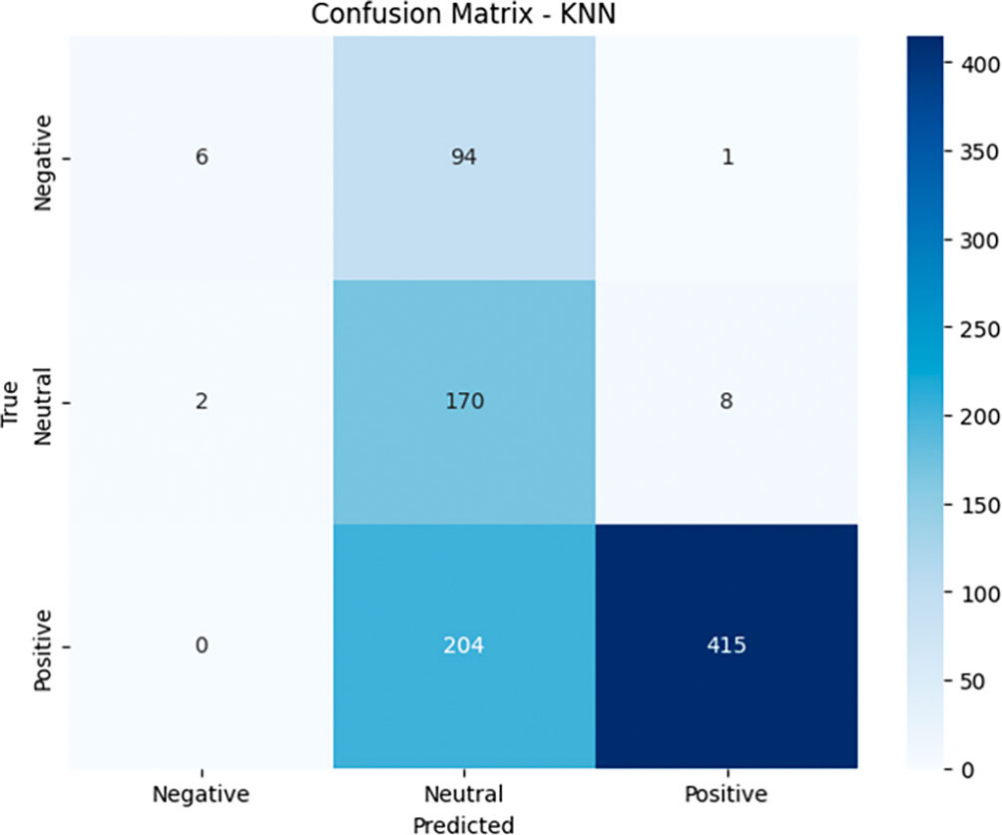
KNN confusion matrix.

In the Confusion Matrix generated by the KNN model (see Figure 14), the distribution of the model’s predictions can be observed as follows. For the Negative category, the model successfully classified 6 data correctly as Negative (true negative), but there were 94 data that should have been Negative but were predicted as Neutral (false positive), and 1 data that was predicted as Positive (false positive). For the Neutral category, the model correctly predicted 170 data points as Neutral (true positive), but there were 2 data points that should have been Neutral but were predicted as Negative (false negative), and 8 data points that were incorrectly predicted as Positive (false negative). As for the Positive category, the model correctly identified 415 data points as Positive (true positive), but there were 204 data points that should have been Positive but were predicted as Neutral (false negative). Overall, although the KNN model performed very well in predicting Positive with high precision, it performed poorly in the Negative and Neutral categories. In the Negative category, the very low recall indicates that the model is almost unable to correctly detect negative data, while in the Neutral category, despite high recall, low precision indicates many incorrect predictions. This model requires significant improvement, especially in separating the Negative and Neutral categories, as well as reducing classification errors in the Neutral category.

### F. ROC Curve

The Receiver Operating Characteristic (ROC) graph shown illustrates a comparison of the performance of the models tested, namely Naive Bayes, SVM, Logistic Regression, Random Forest, Decision Tree, and KNN. In this graph, each model is represented by a curve that shows the relationship between True Positive Rate (TPR) and False Positive Rate (FPR) for various classification thresholds. The area under the ROC curve (AUC) provides an indication of how well the model distinguishes between positive and negative classes.

**Figure 15.**
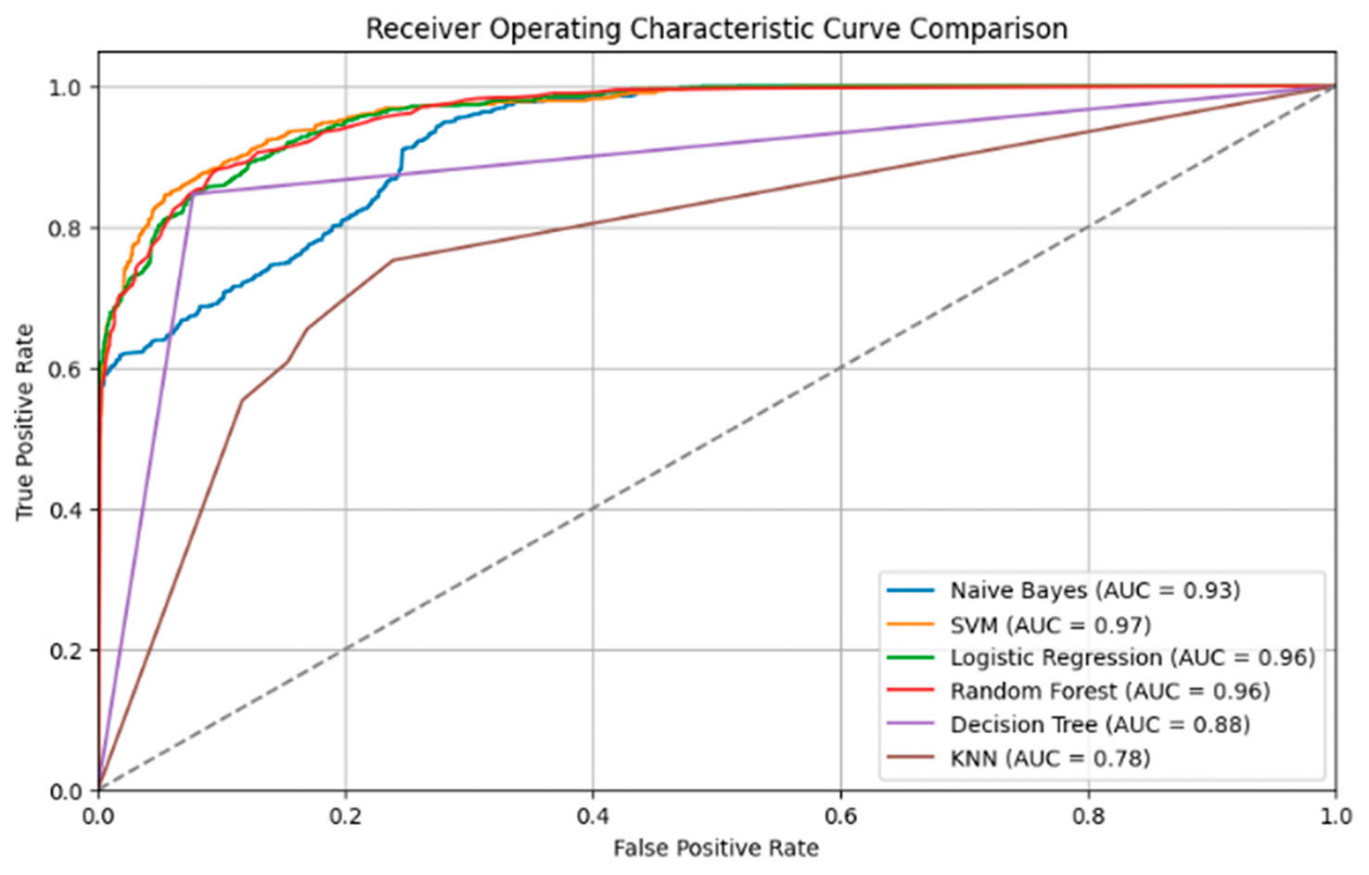
Reciever operating characteristic curve comparison.

From Figure 15, it can be seen that SVM has the best performance with AUC = 0.97, followed by Naive Bayes and Logistic Regression, with AUC = 0.93 and AUC = 0.96, respectively. Both models have curves that are very close to the upper left corner, which indicates their excellent ability to detect positive and negative classes and avoid prediction errors (false positives and false negatives). Random Forest also shows excellent performance with an AUC of 0.96, but is slightly lower than SVM and Logistic Regression. Decision Tree has an AUC = 0.88, which is still quite good, but shows that this model is slightly worse at distinguishing classes than the others. KNN, with an AUC = 0.78, shows the worst performance among the models tested, because its curve is further from the upper left corner and closer to the diagonal line, indicating that this model is hardly any better than random guessing.

Overall, SVM is the most effective model in distinguishing between positive and negative classes, followed by Naive Bayes and Logistic Regression, which have nearly equivalent performance. Decision Tree and Random Forest also show good performance, although slightly lower than the best models, while KNN shows poorer performance in terms of class separation. A higher AUC indicates a better model in separating classes, with SVM at the top of the list.

### G. Model accuracy comparison

**Figure 16.**
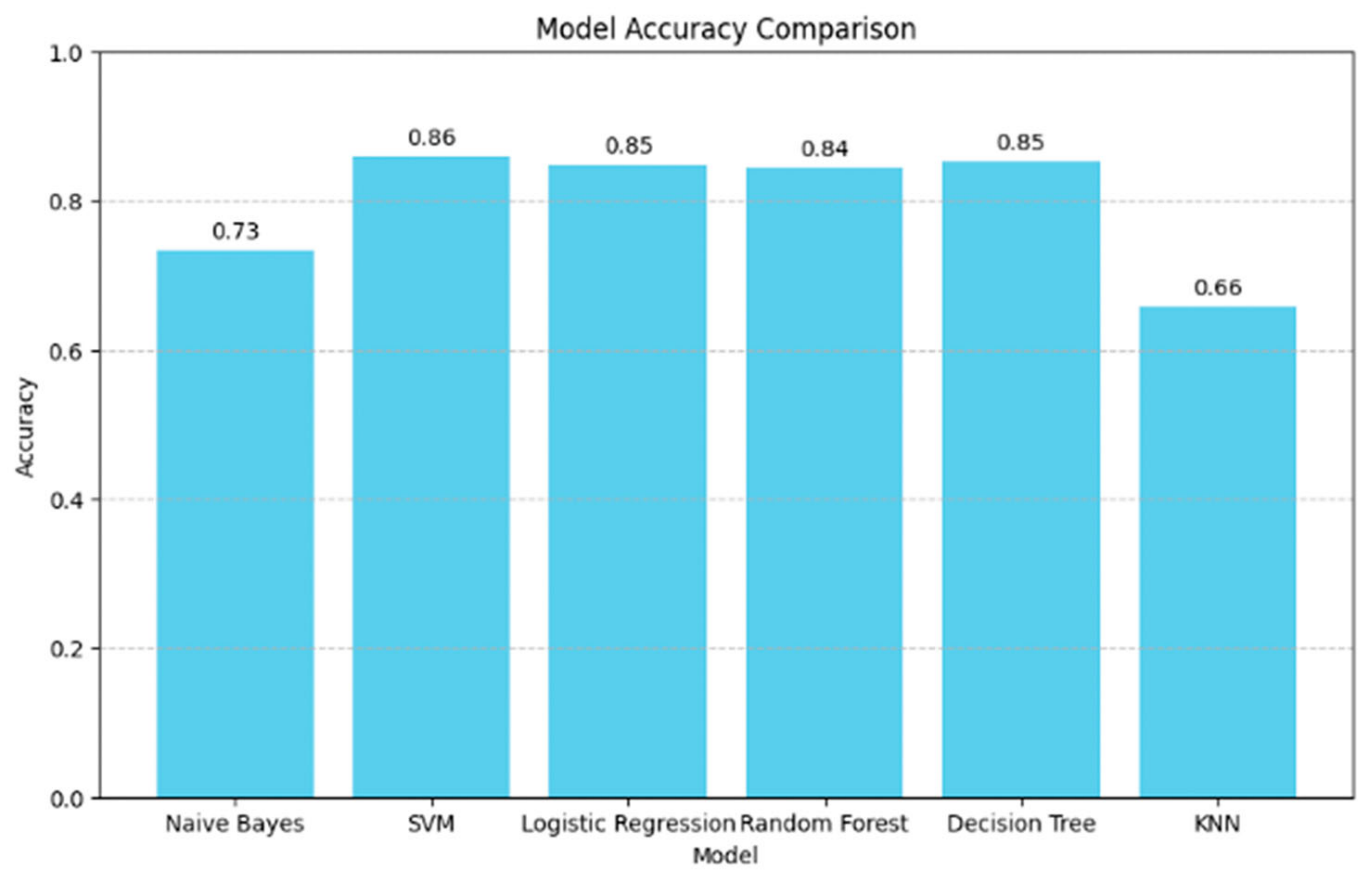
Model accuracy comparison.

This Model Accuracy Comparison graph shows a comparison of the accuracy levels of various models that were tested (see Figure 16), namely Naive Bayes, SVM, Logistic Regression, Random Forest, Decision Tree, and KNN. From the graph, we can see that SVM, Logistic Regression, Random Forest, and Decision Tree have relatively high accuracy, each in the range of 0.84–0.86. Among these models, SVM has the highest accuracy of 0.86, followed by Logistic Regression and Decision Tree, both with an accuracy of 0.85, and Random Forest with 0.84.

In contrast, Naive Bayes showed lower performance with an accuracy of 0.73, which means that this model only managed to correctly predict around 73% of the test data. The KNN model has the lowest accuracy, at 0.66, indicating that this model only successfully predicted about 66% of the data correctly, which is much lower than the other models. Overall, SVM provides the best performance in terms of accuracy, while KNN shows much lower performance, with an accuracy that is only slightly better than random guessing.

**Figure 17.**
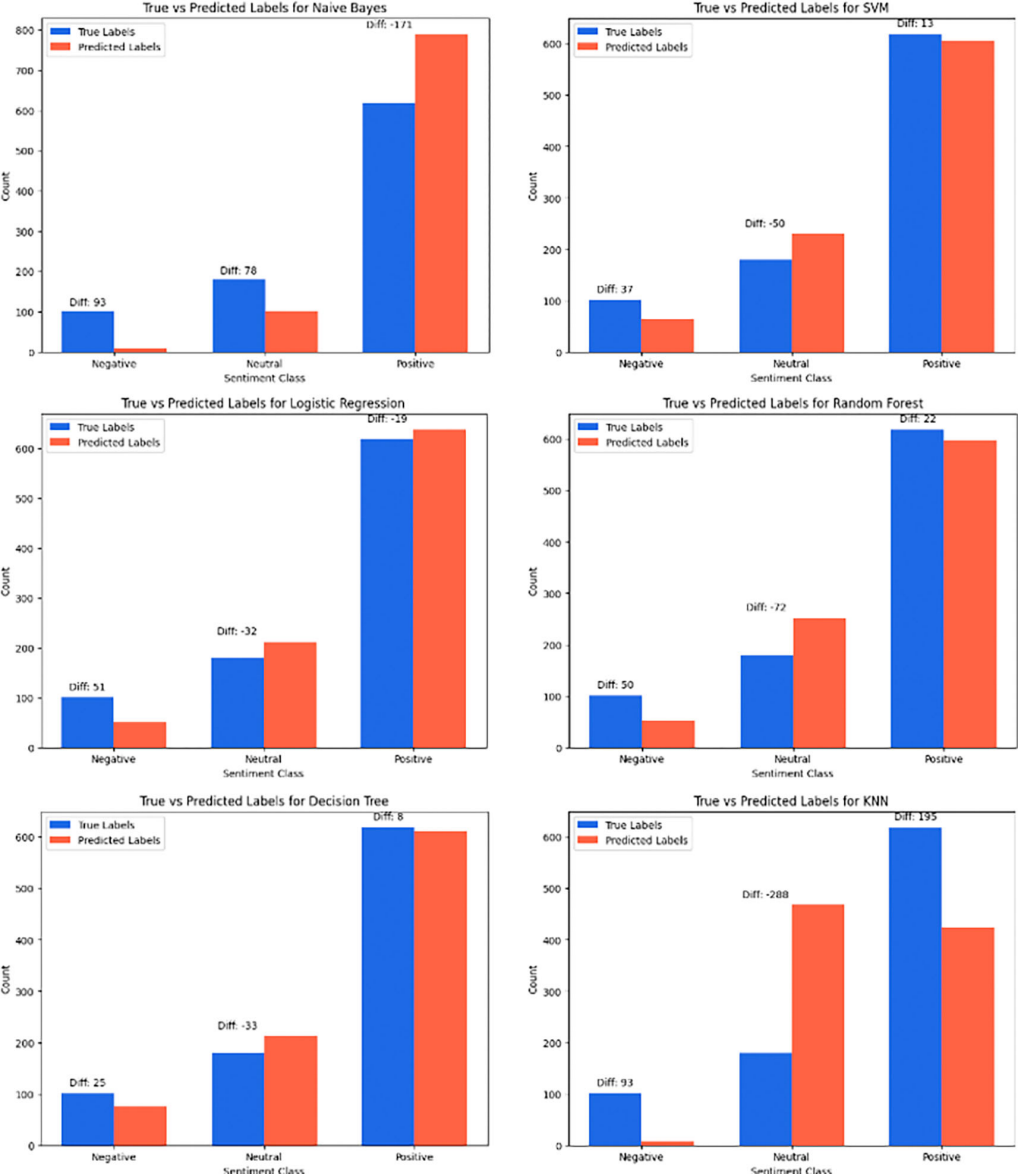
Comparison true and predicted label graph.

Based on the True vs. Predicted Labels graph for each model in Figure 17, we can analyze how well each model predicts the actual sentiment category compared to the generated predictions. In KNN, there is a very large difference in the Neutral category (Diff: 288), which shows that this model often mispredicts Neutral as Positive and has great difficulty in distinguishing this category correctly. For Negative, the error is also quite significant with Diff: 93, indicating that this model often classifies Negative as Neutral or Positive. Conversely, for Positive, the KNN model performs better with Diff: 195, although there are still some prediction errors. The Decision Tree model shows fairly good performance, especially in predicting Positive with Diff: 8, indicating a very low error rate. However, this model still struggles with the Negative and Neutral categories, with Diff: 25 and Diff: 33, indicating that these categories are often mispredicted as other categories. Random Forest shows a similar pattern, with Diff: 50 on Negative and Diff: 72 on Neutral, but excellent performance on Positive (Diff: 22), indicating that this model is superior in detecting positive data.

In Logistic Regression, this model has a Diff: 51 for Negative and Diff: 32 for Neutral, which indicates difficulty in correctly separating these categories. However, for the Positive category, this model has a Diff: 29, which indicates that predictions for Positive are quite accurate despite a few minor errors. SVM shows the greatest difficulty in predicting Neutral, with Diff: 50, which indicates that this model often misclassifies Neutral as Positive. However, in the Negative and Positive categories, this model performs quite well, with Diff: 37 and Diff: 13 respectively, indicating that this model is more effective in separating the Negative and Positive classes. Finally, Naive Bayes shows the greatest difficulty with Negative, with a Diff of 93, meaning that much of the Negative data is predicted as Neutral or Positive. However, in the Positive category, this model is very effective with a Diff of 71, indicating very good predictions despite some errors. All models perform well in predicting Positive, with significant variations in how they handle the Negative and Neutral categories. SVM and Naive Bayes have the most difficulty in separating Neutral, while the Decision Tree and Random Forest models are better at separating the three categories, with KNN showing the largest prediction errors, especially in predicting Neutral and Negative.

## 4. Discussion

### A. Interpretation of key findings

Based on the results of sentiment analysis of user reviews of the Skill Academy app, it was found that the majority of reviews given by users were positive, with 69.4% of reviews categorized as positive sentiment. This indicates that most users are satisfied with their experience using the app, particularly in terms of ease of use, quality of learning materials, and the benefits gained from the app. Words such as “good,” “helpful,” “easy,” and “useful” often appear in positive reviews, which illustrate that users feel the app meets their needs in acquiring vocational skills relevant to the world of work. Positive reviews also express users’ gratitude towards the app, which reflects their appreciation for its functionality and the results obtained from using.

However, there were also 20.5% of reviews categorized as neutral sentiment, indicating that some users felt that the app met their needs but did not provide an exceptional experience that exceeded expectations. This was reflected in words such as “class,” “application,” and “pre-employment,” which showed recognition of the app, but with some aspects that were considered to need further improvement. On the other hand, 10.1% of reviews contained negative sentiment, indicating dissatisfaction among a number of users regarding technical issues with the application. Frequent complaints included “error,” “slow,” and “application failure,” indicating that although the application is widely accepted by most users, there are significant challenges that need to be addressed, particularly in terms of the stability and performance of the application.

### B. Machine learning performance

The SVM (Support Vector Machine) model showed the best performance among the other models, with an accuracy of 85.77%. This model also had excellent precision and recall in the Positive category (precision 0.94, recall 0.92), demonstrating its ability to predict positive reviews with a very low error rate. Although the SVM’s performance in classifying Neutral and Negative is also relatively good, this model still faces challenges in handling Negative sentiment, with a lower recall compared to other categories. Nevertheless, the very high F1-score for the Positive category shows that SVM can effectively minimize errors in both precision and recall, making it a superior choice for sentiment classification in applications. Meanwhile, the Logistic Regression model also showed solid results with an accuracy of 84.89%. This model has a good balance between precision and recall across all sentiment categories, although the precision value for Neutral is slightly lower than for Positive. Random Forest and Decision Tree performed similarly, with accuracies of 83.5% and 84.33%, respectively, and excellent precision for the Positive category. However, both models struggled to identify Negative, as reflected in the lower recall for that category. In addition, Naive Bayes and KNN (K-Nearest Neighbors) showed lower accuracy, at 73.22% and 66.67% respectively, with KNN recording the lowest performance. The KNN model shows high precision for Positive (0.98), but very low recall for Negative, indicating that this model has difficulty handling Negative and Neutral sentiments well. Naive Bayes has better accuracy than KNN, but precision and recall for the Neutral and Negative categories are still relatively low, indicating that this model tends to predict more Positive.

### C. Relevance to Online TVET Admission through the Skill Academy Application

Acceptance of digital-based vocational training platforms, such as Skill Academy, is increasing, especially in online TVET training in Indonesia. ^62, 63^ The findings of this study indicate that ease of use is a key factor supporting high acceptance of app-based TVET among users. Words such as “easy” and “practical” frequently appear in positive reviews, reflecting that users view these apps as user-friendly tools, even for those with limited digital literacy. This finding is in line with the 2021 study by Muktiarni et al., which states that the ease of use of online training platforms is the biggest driving factor in the adoption of technology among users from various backgrounds. ^64^ In addition, the high appreciation for training content such as “material,” “exercises,” and “good” shows that users consider Skill Academy successful in providing job-ready skills or abilities that can be directly applied in the world of work in line with TVET needs. This is highly relevant to the objective of TVET to prepare students to face the challenges of the world of work with practical skills that can be directly applied in the workplace (pre-employment skills). ^65, 66, 67^

However, even though Skill Academy has succeeded in offering a positive experience for most of its users, negative reviews criticizing “bugs” and “slow performance” indicate a gap between user expectations and performance expectancy. These reviews illustrate that although the application is easy to use and useful in terms of training content, frequent technical issues can hinder further adoption by users who are more disconnected or less patient with technical instability. ^68^ This kind of performance expectancy gap can damage perceived usefulness and reduce satisfaction levels, which could potentially hinder adoption in the long term. ^69, 70^ Therefore, to improve perceived ease of use and perceived usefulness, app developers need to focus on improving technical performance to ensure the app runs smoothly and stably on all devices used by various user groups.

### D. Strategic implications for the development of digital TVET

Technical Improvement Priorities: Although overall user satisfaction is quite high, negative feedback focusing on technical issues such as bugs, speed, and errors indicates that technical improvements should be a primary focus. Recurring performance issues not only damage the user experience, but also have the potential to hinder the adoption of new applications and user retention. ^71^ Therefore, investing in quality assurance, application testing, and performance optimization is essential to turn negative feedback into a more positive experience and increase user trust in the application. ^72, 73^

Leverage the Power of Content: Reviews that show high appreciation for training content (for example, words such as “material,” “exercises,” and “good” dominate positive reviews) indicate that Skill Academy’s content strategy successfully meets user needs and is relevant to the workplace. To increase adoption and user satisfaction, the platform may consider expanding its training content with similar quality standards and introducing new industries or skills that are more relevant to the ever-evolving labor market demand. ^74, 75, 76^ By enriching its content offerings, Skill Academy can continue to meet the needs of training participants and expand its reach to new users.

User Segmentation and Personalization: Analyzing user segmentation based on TVET course categories and user demographics can generate more specific insights into the needs and behaviors of each segment. ^77, 78^ By identifying courses with the highest sentiment ratings, the platform can allocate training and marketing resources more efficiently, while understanding which groups are most responsive to different types of learning content. ^79^ In addition, personalizing course recommendations based on user behavior and community sentiment allows Skill Academy to improve content relevance and user engagement. ^80^ Personalized recommendations can help platforms offer a more focused and relevant learning experience tailored to individual needs, thereby potentially increasing user satisfaction and the duration of app usage in the long term.

## 5. Conclusions

This study has provided valuable new insights into user sentiment toward the online TVET learning platform, Skill Academy, through analysis of user reviews on Google Play Store using six different machine learning algorithms. The results show that Skill Academy has achieved significant success in the Indonesian market with a high level of user acceptance, as indicated by a positive sentiment score of 69.4%. This shows that the platform meets user needs in terms of ease of use, quality of learning content, and practical benefits for skills development. Specifically, the study revealed that the Support Vector Machine (SVM) model showed the best performance, with an accuracy of 85.77% and an AUC of 0.97, demonstrating its superiority in classifying sentiment. However, despite positive user acceptance, 10.1% of reviews mentioned technical issues, including application bugs, slow loading speeds, and performance glitches. These technical issues must be addressed to maintain user satisfaction and ensure sustainable growth. Further analysis identified three strategic priorities for future development: 1) Technical improvements and performance optimization, 2) Content expansion to meet evolving industry demands, and 3) Personalization and segmentation of user experiences to increase engagement. These steps will help Skill Academy remain the leading digital TVET platform in Indonesia, driving sustainable digital transformation in vocational education. However, this study has limitations. As a cross-sectional quantitative analysis, causal relationships cannot be drawn. In addition, the data is limited to reviews from the Google Play Store, and future research should consider data from other platforms. Further studies should also analyze user sentiment over time to understand its evolution as updates are made, and explore specific features that influence user satisfaction and dissatisfaction. This study provides a comprehensive understanding of Skill Academy’s position in the digital TVET space in Indonesia. With a continued focus on user feedback and strategic improvements, Skill Academy can strengthen its position as a leading platform for vocational education in Indonesia, contributing to broader digital transformation in the sector. Future research should build on these findings by conducting longitudinal studies to establish causal relationships and test the generalization of these results across other platforms and education sectors.

## Ethical considerations

This study did not involve direct interaction with human participants. The research exclusively analyzed publicly available user reviews obtained from the Google Play Store for the Skill Academy application. All data were collected in accordance with the platform’s publicly accessible interfaces, and no private, sensitive, or personally identifiable information was collected, stored, or analyzed. Usernames and any identifiable information were excluded during data processing, and all analyses were conducted in aggregate form. As the study relied solely on secondary, publicly available data, formal ethical approval and informed consent were not required, in line with institutional research ethics guidelines and established standards for social science research using public online data.

## Author contribution

D.D = Conceptualization, Formal Analysis, Funding Acquisition, Methodology, Resources, Supervision, Validation, Writing – Review & Editing; Y.A.F = Formal Analysis, Investigation, Methodology, Resources, Writing – Original Draft Preparation, Validation, Writing – Review & Editing; K. M = Formal Analysis, Investigation, Methodology, Resources, Software, Validation, Writing – Review & Editing; M.R.M = Data Curation, Formal Analysis, Writing – Original Draft Preparation, Resources; A.A.S = Data Curation, Formal Analysis, Funding Acquisition, Project Administration, Resources; RBS = Funding Acquisition, Project Administration, Resources, Visualization; B.B.B = Funding Acquisition, Methodology, Validation, Visualization. R.J.S = Data Curation, Formal Analysis, Investigation, Methodology, Software, Visualization, Writing – Original Draft Preparation, Writing – Review & Editing; S. S = Funding Acquisition, Project Administration, Validation, Writing – Review & Editing. All authors have read and agreed to the published version of the manuscript.

## Data Availability

The datasets supporting the findings of this study are available in the Zenodo repository under an open license. The shared data include anonymized and curated outputs derived from publicly available user reviews of the Skill Academy application on the Google Play Store, along with sentiment labels, processed text features, and data used to generate figures and tables reported in this article. Raw user reviews cannot be redistributed due to the Google Play Store terms of service. However, all preprocessing steps and data transformation procedures are fully documented to ensure reproducibility. The datasets supporting the findings of this study are available in the Zenodo repository ^81^ at: https://doi.org/10.5281/zenodo.19916911. Source code available from: https://github.com/yanuaragung2025/Yanuar-Agung-Fadlullah/tree/1. Archived software available from: https://doi.org/10.5281/zenodo.19916911. ^81^ **License**: MIT License. This study reports findings from a quantitative text mining and machine learning analysis of user reviews obtained from a mobile application platform. As the study does not involve clinical trials, systematic reviews, experimental interventions, or in vivo research, no specific reporting guideline checklist such as CONSORT, PRISMA, or ARRIVE applies. The manuscript has been prepared to ensure transparency and reproducibility by clearly reporting data sources, preprocessing steps, analytical methods, model evaluation procedures, and study limitations, in accordance with best practices for computational social science and machine learning research.
