# Sentiment Analysis of Acceptance TVET Online Courses on the Skill Academy App from Google Play: Leveraging Text Mining with Comparison Machine Learning Model

**DOI:** 10.12688/f1000research.181728.2

**Published:** 2026-09-24

**Authors:** Darmono Darmono, Yanuar Agung Fadlullah, Khakam Ma’ruf, Muhamad Riyan Maulana, Apry Aditya Saputra, Ramzy Bin Sulaiman, Bagus Banjar Bagaskara, Rizal Justian Setiawan, Sahril Sahril

**Affiliations:** 1 Civil Engineering Education, Faculty of Engineering, Universitas Negeri Yogyakarta, Yogyakarta, Special Region of Yogyakarta, 55281, Indonesia; 2 Technology and Vocational Education, Universitas Negeri Yogyakarta, Yogyakarta, Special Region of Yogyakarta, 55281, Indonesia; 3 Industrial Engineering, Faculty of Engineering, Universitas Gadjah Mada, Yogyakarta, Special Region of Yogyakarta, 55281, Indonesia; 4 Mechanical Engineering Education, Faculty of Engineering, Universitas Negeri Yogyakarta, Yogyakarta, Special Region of Yogyakarta, 55281, Indonesia; 5 Information Technology, Faculty of Engineering, Universitas Gadjah Mada, Yogyakarta, Special Region of Yogyakarta, 55281, Indonesia; 6 Asia and China Studies, College of Law and Politics, National Chung Hsing University, Taichung, Taichung City, 402204, Taiwan; 7 Industrial Engineering and Management, College of Engineering, Yuan Ze University, Zhongli District, Taoyuan City, 32003, Taiwan

**Keywords:** Machine Learning, Sentiment Analysis, Skill Academy App, Text Mining, TVET Acceptance, VADER Sentiment Analyzer.

## Abstract

**Background:**

Online Technical and Vocational Education and Training (TVET) has expanded rapidly in Indonesia through platforms such as Skill Academy. User reviews provide valuable insights into user acceptance, but their volume makes manual analysis impractical. Therefore, this study applies text mining to analyze user sentiment and compare eight machine learning models for sentiment classification.

**Methods:**

The study used 3,000 reviews collected via web scraping using the google-play-scraper library. The data was then anonymized, cleaned, translated into English, and automatically sentiment-labeled using VADER. The validity of the labeling was verified by comparing it with TextBlob and the original star-rating categories using Cohen’s Kappa. Eight classification algorithms Naive Bayes, SVM, Logistic Regression, Random Forest, Decision Tree, KNN, XGBoost, and LightGBM were trained using a TF-IDF pipeline with GridSearchCV and five-fold cross-validation.

**Results:**

The labeling validation results showed moderate agreement, with a Cohen’s Kappa of 0.500 for TextBlob and 0.533 for the original star rating categories. On the test data, Logistic Regression achieved the best performance with an accuracy of 85.48%, a macro-F1 score of 76.08%, a balanced accuracy of 80.86%, and a macro-AUC of 0.9486, followed by SVM and XGBoost. The sentiment distribution was dominated by positive reviews at 75.31%, followed by negative reviews at 15.57%, and neutral reviews at 9.12%. The SMOTETomek experiment improved balanced accuracy and recall for the minority class but reduced macro-precision. Word cloud and word frequency analysis revealed that the main complaints were related to the app being slow, resource-intensive, and prone to errors, while user appreciation centered on ease of learning and the quality of the material.

**Conclusions:**

Research shows that logistic regression achieved the best classification performance. While user sentiment was largely positive, improving application stability remains essential for sustaining user acceptance, enhancing learning experiences, and supporting long-term adoption of online TVET platforms.

## 1. Introduction

Digital transformation has changed the global education landscape, particularly in Technical and Vocational Education and Training (TVET). ^1^ ^,^ ^2^ The integration of digital technologies into vocational education has created broader opportunities for flexible, skill-oriented, and lifelong learning through online platforms. ^3^ Mobile applications have also become an important medium for delivering educational content because they allow users to access training materials, learning activities, and skill-development resources anytime and anywhere. ^4^ ^,^ ^5^ In Southeast Asia, particularly Indonesia, the adoption of online learning platforms for TVET has accelerated significantly over the past five years. ^6^ ^,^ ^7^ Skill Academy, as one of Indonesia’s online course platforms in the Google Play Store ecosystem, has attracted users seeking vocational and professional skills training. ^8^ Along with this growth, user reviews on the Google Play Store have become a valuable source of naturally occurring feedback for understanding users’ perceptions, satisfaction, complaints, and experiences regarding TVET content quality and application features. ^9^ ^,^ ^10^ However, in this study, user reviews are interpreted as expressions of user perception and sentiment, not as direct measurements of formal technology acceptance constructs.

Understanding users’ perceptions and sentiments toward online learning platforms is a crucial step for the continuous improvement of digital vocational education services. ^11^ ^,^ ^12^ User reviews stored on the Google Play Store contain authentic feedback about learning experiences, course content quality, teaching effectiveness, application usability, and overall user satisfaction. ^13^ ^,^ ^14^ Nevertheless, application developers and education stakeholders often face substantial challenges in extracting meaningful insights from large volumes of unstructured review data. ^15^ ^,^ ^16^ Therefore, sentiment analysis and machine learning can serve as practical approaches for classifying user review sentiment into positive, negative, and neutral categories and for transforming raw textual feedback into actionable insights for application developers and TVET stakeholders. ^17^ ^,^ ^18^ In this study, the analysis is conducted on 3,000 Indonesian-language Google Play reviews of the Skill Academy application collected using the Google Play scraper, with 2,993 reviews retained after minimal text cleaning.

Sentiment analysis, also known as opinion mining, is a subdiscipline of Natural Language Processing (NLP) that focuses on the automatic identification, extraction, and classification of opinions and emotions contained in text. ^19^ This technique has been applied in various domains, including customer feedback analysis, product evaluation, and the analysis of public perceptions of digital services and education. ^20^ ^,^ ^21^ Research by Marry A et al. 2025 shows that sentiment analysis can provide actionable insights into student satisfaction, the effectiveness of online learning, and the quality of educational content. ^22^ Sharin et al. 2025 analyzed the sentiment of TVET discussions on social media and found that machine learning-based algorithms can identify sentiment patterns with good performance, opening opportunities for sentiment analysis in TVET-related educational application reviews. ^23^

Previous studies have also widely used VADER, or Valence Aware Dictionary and Sentiment Reasoner, as a lexicon-based sentiment analysis tool optimized for social media and informal text because it can capture sentiment intensity through capitalization, punctuation, and intensifier words. ^24^ ^,^ ^25^ VADER has been reported to provide consistent and useful results for sentiment analysis, particularly on social media texts and user reviews. ^26^ ^,^ ^27^ However, because VADER is primarily optimized for English text, its application to Indonesian reviews requires translation, which may introduce changes in nuance, idiomatic meaning, negation, or sentiment intensity. To reduce meaning distortion before translation, this study applies only minimal Indonesian text cleaning, such as removing URLs, emails, numbers, excessive characters, and duplicate spaces, without stemming or stopword removal before machine translation. ^28^ The cleaned Indonesian reviews are then translated into English before VADER is applied. Therefore, the sentiment categories produced in this study should be understood as VADER-based automatic sentiment labels rather than independently validated human sentiment judgments.

The development of sentiment analysis has also expanded from classical machine learning toward more advanced contextual language models and explainable artificial intelligence. Transformer-based approaches such as BERT, RoBERTa, and language-specific models such as IndoBERT have strengthened NLP research because they are able to capture contextual meaning more effectively than traditional bag-of-words representations. ^29^ ^,^ ^30^ At the same time, explainable AI has become increasingly important because model accuracy alone does not fully explain why a model assigns a particular sentiment class to a review. ^31^ Despite these recent developments, classical machine learning models remain relevant as transparent, computationally efficient, and reproducible baselines, particularly when applied with TF-IDF features and evaluated using careful validation procedures. ^32^ The comparative evaluation of classical models therefore remains valuable for applied educational technology research, especially in contexts where reproducibility, interpretability, and computational simplicity are important considerations. ^18^

In developing a sentiment analysis model for user reviews, the selection of machine learning algorithms is an important factor that affects the accuracy of sentiment classification and the quality of insights produced. Various machine learning algorithms are widely used for text classification tasks, including sentiment analysis, and each algorithm has distinct assumptions, advantages, and limitations. Naive Bayes is a probabilistic classifier that remains relevant as a baseline model because of its simplicity, computational efficiency, and ability to handle high-dimensional text features. ^33^ ^,^ ^34^ Support Vector Machine (SVM), in contrast, is known for its ability to construct an optimal separating hyperplane between classes and often performs well in high-dimensional feature spaces such as TF-IDF-based text classification. ^35^ ^,^ ^36^ Logistic Regression offers an attractive combination of competitive accuracy and interpretability, making it useful for understanding patterns that influence classification results. ^37^ Decision tree-based algorithms such as Random Forest and Decision Tree provide flexibility in handling non-linear relationships and interactions between features, while Random Forest often functions as a strong baseline in sentiment classification tasks. ^38^ ^,^ ^39^ Meanwhile, K-Nearest Neighbors (KNN), as a non-parametric algorithm, can capture local similarity patterns in data, although it may require higher computational cost for larger datasets because distance calculations must be performed during prediction. ^38^ ^,^ ^40^ In addition to these classical models, this study also evaluates XGBoost and LightGBM as boosting-based models to provide a broader comparison between conventional machine learning and ensemble-based approaches.

User reviews on the Google Play Store for educational applications contain important information about how users perceive the quality of online learning, course content, application features, and overall user experience. ^41^ ^,^ ^42^ Positive sentiment in reviews may reflect ease of access, ease of use, flexibility of time, and perceived completeness of learning materials, all of which contribute to a favorable learning experience. ^43^ Negative sentiment may reveal technical issues, usability problems, content limitations, or mismatches between user expectations and actual application performance. ^13^ ^,^ ^44^ Neutral sentiment usually reflects a more balanced or less emotionally explicit view, in which users are neither fully satisfied nor clearly disappointed. ^45^ By analyzing the distribution and patterns of these sentiments, application developers can identify strengths that should be maintained and weaknesses that require priority improvement. ^46^ ^,^ ^47^ In addition, insights from user sentiment can help TVET stakeholders understand how actual users perceive the quality and effectiveness of online vocational learning in Indonesia. ^48^ Nevertheless, such sentiment patterns should not be interpreted as direct evidence of technology acceptance unless established acceptance constructs, such as perceived usefulness, perceived ease of use, behavioral intention, and actual use, are explicitly measured.

There is a significant research gap in the analysis of user sentiment toward online TVET platforms in Indonesia, especially in relation to the Skill Academy application as one of the vocational learning platforms available in the Indonesian mobile learning ecosystem. ^49^ Previous research on sentiment analysis in the education sector has generally focused more on feedback from general social media or broader digital learning contexts, without sufficient attention to online TVET platforms. ^50^ Beyond the application domain, several methodological limitations also remain important. Many sentiment analysis studies rely on automatically generated labels without sufficiently examining label consistency, use translation-based preprocessing with limited discussion of possible meaning shifts, or apply feature extraction before data splitting and cross-validation, which may introduce data leakage and produce overly optimistic estimates of model performance. In addition, model comparisons are often reported mainly through accuracy, although application review datasets commonly show imbalanced class distributions. Therefore, evaluation metrics such as macro-F1, balanced accuracy, class-specific recall, ROC-AUC One-vs-Rest, and precision-recall analysis are needed to provide a more reliable assessment of model performance across sentiment classes.

This study addresses these gaps by conducting a systematic comparative analysis of VADER-based sentiment classification on Google Play Store reviews of the Skill Academy application. Sentiment categories are generated using VADER after translation into English; therefore, the resulting labels are treated as automatic sentiment labels rather than independently validated human sentiment judgments. ^51^ To examine the consistency of the automatic labels, this study compares VADER labels with two independent signals: TextBlob sentiment labels and star-rating-based sentiment categories derived from the original user ratingsThe agreement between these sources is assessed using agreement rate and Cohen’s Kappa. ^52^ ^,^ ^53^

To reduce methodological bias in model evaluation, this study applies a pipeline-based machine learning workflow in which TF-IDF vectorization is integrated into model training and hyperparameter tuning. ^54^ The data are first divided into stratified training and holdout test sets, and TfidfVectorizer is fitted only within the training process inside the cross-validation pipeline. This allows feature extraction to be fitted only on the training subset within each cross-validation fold and helps reduce the risk of data leakage. ^55^ The TF-IDF configuration uses a maximum of 5,000 features, unigram and bigram features, minimum document frequency of two, lowercase normalization, and sublinear term frequency scaling. Class imbalance is also considered through class-weighting strategies and, when applicable, resampling strategies implemented within the cross-validation pipeline rather than applied globally before model training. In particular, SMOTETomek is examined as an additional experiment for selected models, namely SVM and Random Forest, by placing the resampling step inside an imbalanced-learn pipeline so that resampling is applied only to the training portion of each cross-validation fold. ^56^ ^–^ ^58^

The novelty of this study lies in four main aspects. First, it applies a leakage-aware machine learning pipeline by integrating TF-IDF vectorization into cross-validation and hyperparameter tuning. Second, it explicitly acknowledges the limitations of VADER-based automatic labeling and translation-based sentiment analysis, thereby avoiding the assumption that the resulting labels represent human-validated ground truth. Third, it evaluates model performance using metrics that are more appropriate for imbalanced sentiment data, including macro-F1, balanced accuracy, class-specific recall, ROC-AUC One-vs-Rest, and precision-recall analysis, rather than relying only on accuracy. Fourth, it focuses on the relatively underexplored domain of Indonesian online TVET application reviews, where user feedback can provide practical insights into perceived learning experience, application usability, content quality, and technical issues. In addition, this study strengthens reproducibility by saving generated tables and visualizations as supplementary outputs, including review data summaries, sentiment distributions, cross-method agreement tables, model evaluation reports, confusion matrices, ROC-AUC summaries, precision-recall curves, learning curves, and feature-importance outputs.

Based on these gaps and methodological considerations, this study is guided by three research questions (RQ). First, how are the sentiment distribution and review characteristics of Skill Academy users represented in Google Play Store reviews? Second, to what extent do VADER-based automatic sentiment labels show consistency with TextBlob sentiment labels and star-rating-based sentiment categories? Third, which machine learning model provides the best classification performance for VADER-based sentiment labels when evaluated using metrics suitable for imbalanced multiclass data?

This research has theoretical, methodological, and practical significance for stakeholders in the digital TVET education ecosystem. For developers of the Skill Academy application and similar TVET platforms, the findings provide user-review-based evidence on how users express satisfaction, complaints, and perceived learning experiences through application reviews. These insights can support user experience optimization, course content improvement, technical issue prioritization, and more responsive product development. For machine learning practitioners, data scientists, and researchers, this study provides an empirical benchmark on the performance of standard machine learning and boosting algorithms in the domain of VADER-based sentiment classification for TVET learning applications. These findings can inform algorithm selection in similar sentiment analysis projects, improve understanding of the trade-off between model complexity and interpretability, and contribute to the body of knowledge on sentiment classification for e-learning and mobile education platforms. For policymakers, educators, and TVET stakeholders, the findings provide user-review-based insight into how Indonesian users describe their experiences with online TVET learning platforms, which can support further evaluation of digital transformation strategies in vocational education.

## 2. Method

### 2.1 Research design

This study employs a quantitative research design using text mining and machine learning approaches to classify the sentiment of user reviews for the Skill Academy app on Google Play. The research process consists of eight main stages: (1) data collection via web scraping; (2) data anonymization; (3) text preprocessing; (4) automatic sentiment translation and labeling; (5) cross-validation of labels; (6) exploratory data analysis; (7) training and tuning of the classification model; and (8) evaluation and comparison of model performance, including experiments on handling class imbalance. The entire process was conducted in accordance with the principle of reproducibility by setting the random state to 42 for all randomization processes.

### 2.2 Data collection

Review data was collected from the Skill Academy app on Google Play using the Python library ‘google-play-scraper’, employing a batch scraping approach with continuation tokens until the target of 3000 reviews was reached. Each review record consists of the user’s name, the review content, a star rating (1–5), and the date the review was written (at). A total of 3000 raw reviews were successfully collected. As a descriptive baseline and an independent validation tool for subsequent stages, the star ratings were grouped into three categories: Negative (ratings 1–2), Neutral (rating 3), and Positive (ratings 4–5). To protect user privacy, the username (userName) column was hashed using the SHA-256 algorithm to create an anonymous identifier (user_id) before the data was used in the text cleaning, sentiment labeling, or visualization stages. The original username column was no longer used in any subsequent stages of the analysis.

### 2.3 Preprocessing data

The review text was minimally cleaned (removal of excess non-alphabetic characters, normalization of spaces, and removal of empty reviews) without stemming at this stage. In the initial iteration, the minimally cleaned text was translated into English and labeled with VADER sentiment. After cleaning, the number of valid (non-empty) data points decreased from 3000 to 2993 reviews. Table 1 shows descriptive statistics on text length calculated to provide additional quantitative context beyond the machine learning model, including the number of characters and the number of words before and after cleaning.

**Table 1.** Descriptive statistics on the length of review texts.

Statistics	Number of characters (Raw)	Word count (Raw)	Word count (Clean)
N	2993	2993	2993
Mean	73.57	11.08	10.94
Standard Deviation	84.26	13.18	12.95
Minimum	2	1	1
Quatile 1 (25%)	17	2	2
Median (50%)	44	6	6
Quartile 3 (75%)	98	14	14
Maximum	500	93	90

**Figure 1.**
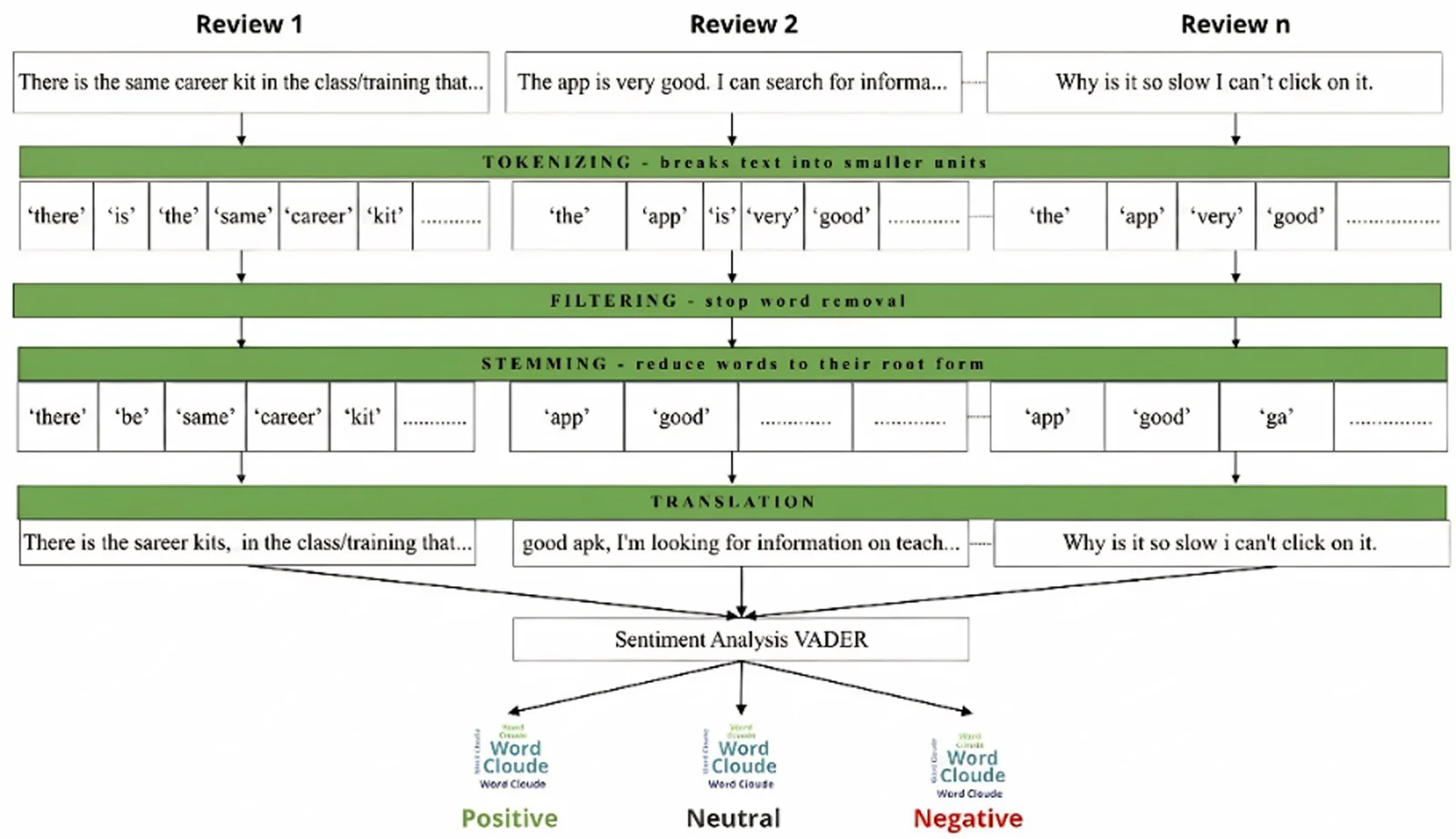
Text preprocessing steps.

Text preprocessing is a stage to ensure data quality and model accuracy. This study implements five preprocessing stages sequentially, as performed by Dias et al. ^59^ which is visualized in Figure 1.

1. Cleansing Data
  The first stage of preprocessing involves removing irrelevant characters from the text data. This includes removing links (URL), user mentions, hashtags, special characters (?, $,.,!, ^, _, “,), (, −, +,,), and emojis converted to ASCII characters. This process is performed by matching regular expression patterns (re.sub) to ensure consistency and efficiency in cleaning 3000 reviews.
2. Case Folding
  All text is converted to lowercase and white space normalization is performed. This step ensures that identical words with different capitalization (e.g., “Good” and “good”) are treated as the same token, thereby improving the consistency of the analysis.
3. Tokenizing
  The cleaned text is separated into individual word units (tokens) based on spaces. The application uses a simple separation method with filtering to remove empty tokens. The output of this stage is a list of individual words that are ready for the next processing stage.
4. Filtering
  Meaningless words are common words that are considered to make no significant contribution to sentiment analysis. This study applies comprehensive meaningless word removal using a combination of three sources: (1) Indonesian meaningless words from NLTK (Natural Language Toolkit), (2) meaningless words from the Sastrawi library (specific to Indonesian), and (3) 13 additional meaningless words specific to the context of mobile application reviews (via, nya, dr, sy, q, pd, &, di, min, sih, ny, aj,,). The total number of meaningless words used is a combination of the three sources with duplication removed to avoid repetition. The use of multiple lists of meaningless words ensures the complete removal of meaningless words while retaining words that carry important sentiment.
5. Stemming
  Stemming is the process of reducing inflected and derived words to their base or root form. ^60^ This study uses Sastrawi StemmerFactory, which is specifically designed for Indonesian, enabling accurate handling of Indonesian morphology. Each word in the token list is stemmed, so that variations of the same word (e.g., “latihan”, ‘melatih’, “dilatih”) are converted to the same root form, improving feature consistency and reducing dimensionality. The output of the stemming stage is normalized text stored in the ‘clear’ column of the dataset.
6. Sentiment Translation and Tagging
  Indonesian review text that has been minimally cleaned up is translated into English using the Google Translate API so that it can be processed by the VADER sentiment lexicon, which was developed for English-language text. The translated reviews are classified into sentiment categories using the VADER (Valence Aware Dictionary and sEntiment Reasoner) Sentiment Analyzer. ^61^ ^,^ ^62^ VADER was chosen for its superiority in analyzing sentiment in informal text, including emoticons, capital letters, and punctuation marks that often appear in mobile app reviews. ^26^ ^,^ ^63^ The VADER Sentiment Analyzer calculates a compound score for each translated review. The Compound Score value is determined based on Equation 1.
$$C=\frac{\sum_{i=1}^{n}s_{i}}{\sqrt{\sum_{i=1}^{n}s_{i}^{2}+\alpha^{2}}}$$ (1)
  *C =* compound score.
  S *_i_* = denotation of valence score of the *i*-th token.
  α = normalization constant.
*n* = total number of token in text.

The core of decision-making in VADER is the composite score, which is a compound score calculated by summing the valence scores of each token in the text, then normalizing that sum within the range of −1 to +1. ^27^ Hernández-Pérez R 2024 classifying sentiment using three thresholds ^64^:

• Positive: compound score ≥ 0.05
• Negative: compound score ≤ −0.05
• Neutral: −0.05 < compound score < 0.05

A threshold of 0.05 was chosen to provide a reasonable margin in separating positive and negative sentiment from neutral sentiment, allowing for a more detailed classification of user opinions.

### 2.4 Cross-validation of sentiment labels

The quality of VADER labels is validated through two independent automated processes. First, VADER labels are compared with labels generated by TextBlob using a polarity threshold of >0.1 for Positive and < −0.1 for Negative. ^65^ ^,^ ^66^ Second, the VADER label was compared with the original star rating category entered by users when submitting reviews (Rating_Category), as a form of independent external validation of the two lexicon-based methods. The agreement rate and Cohen’s Kappa statistic were calculated for each comparison, with the following interpretations: ≤20—slight, 21–40—fair, 41–60—moderate, 61–80—substantial, and 81–100—almost perfect. ^67^

### 2.5 Data analysis and explanation

Exploratory analysis was conducted to understand the qualitative and quantitative characteristics of the data, including: word cloud visualization by sentiment class, frequency analysis of single words (unigrams) and two-word phrases (bigrams) using CountVectorizer, distribution of review lengths by sentiment class (box plots and violin plots), as well as trends in sentiment proportions over time (monthly) based on the date the reviews were written.

### 2.6 Data preparation for machine learning

The data was split into training and test sets in an 80:20 ratio, stratified by sentiment class, resulting in 2394 training data points and 599 test data points. The data split was performed before the feature extraction process to ensure that no information from the test set leaked into the training process. Unlike common practice, which involves directly fitting the TfidfVectorizer to the entire training dataset before cross-validation, this study places the TfidfVectorizer within a scikit-learn Pipeline object along with each classifier, so that the TF-IDF fitting process is repeated for each fold of the StratifiedKFold cross-validation. In this way, the vocabulary and inverse document frequency (IDF) weights are learned only from a subset of the training data in each fold, rather than from the entire training dataset, making the model performance estimates obtained through cross-validation more valid and free from optimistic bias. ^68^

### 2.7 Model training and hyperparameter tuning

Eight classification algorithms were trained and tuned using GridSearchCV with a StratifiedKFold scheme (k = 5) that optimizes the macro-F1 score: Multinomial Naive Bayes, Support Vector Machine (SVM) with a linear kernel, Logistic Regression, Random Forest, Decision Tree, K-Nearest Neighbors (KNN), XGBoost, and LightGBM. The classical models (Naive Bayes, SVM, Logistic Regression, Random Forest, Decision Tree, KNN) use the standard scikit-learn pipeline, while XGBoost and LightGBM use additional numerical label encoding (LabelEncoder) but still retain the pipeline structure with TfidfVectorizer included.

### 2.8 Model evaluation

Model performance was evaluated on test (holdout) data that was not used at all in the tuning process, using the following metrics: accuracy, macro-F1, weighted-F1, balanced accuracy, macro-precision, macro-recall, recall per class, confusion matrix, and ROC-AUC for the One-vs-Rest (OvR) scheme, both macro and micro. Macro-F1 and balanced accuracy were chosen as the primary metrics because the distribution of sentiment classes in this dataset is imbalanced; therefore, relying solely on accuracy could potentially mislead the interpretation of the model’s performance on minority classes (Negative and Neutral). ^69^ Precision-recall curves by class are also presented for the best-performing model, given that precision-recall curves are more informative than ROC curves in minority-class scenarios. ^70^ The learning curve was also analyzed to check for signs of overfitting or underfitting, as well as feature importance for tree-based models (XGBoost and LightGBM).

### 2.9 Addressing class imbalances

As an additional experiment, the SMOTETomek resampling technique (a combination of SMOTE oversampling and Tomek Links undersampling) was applied to the two models that performed best in the initial experiment. ^71^ ^,^ ^72^ Unlike the common practice of applying ‘fit_resample’ to the entire training dataset before model training, resampling in this study is incorporated into the ‘imblearn.pipeline.Pipeline’, so that SMOTETomek is applied only to a subset of the training data in each cross-validation fold, while the test (holdout) data remains unaffected by the resampling intervention. The parameter ‘k_neighbors = 3’ is used to accommodate the limited number of minority class instances in each fold.

### 2.10 Computing devices and environments

All analyses were performed using Python with the following main libraries: pandas 2. 2.2, numpy 2.0.2, scikit-learn 1.6.1, xgboost 3.3.0, lightgbm 4.6.0, matplotlib 3.10.0, seaborn 0.13.2, vaderSentiment, TextBlob, Sastrawi, wordcloud, imbalanced-learn, and googletrans. The random seed was set to 42 throughout the randomization process to ensure the reproducibility of the results.

## 3. Result and discussion

### 3.1 Data characteristics and rating distribution

**Figure 2.**
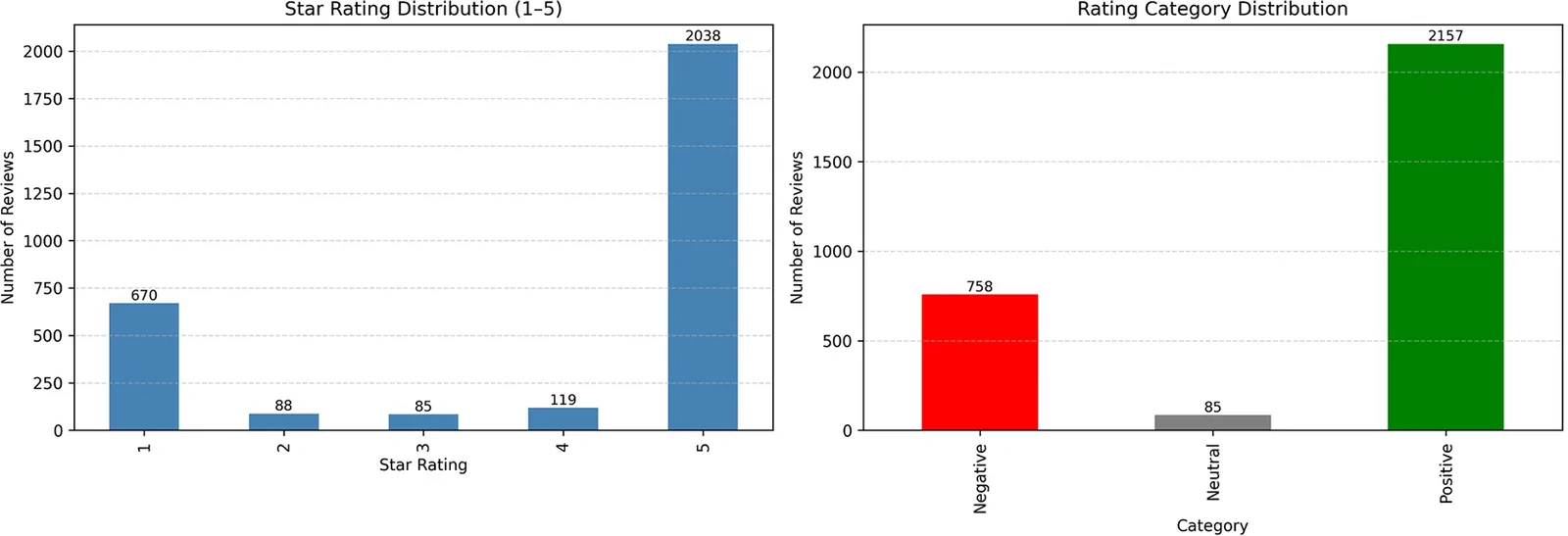
Distribution of star ratings (left) and rating categories (right) in Skill Academy app reviews.

A total of 3,000 reviews were collected from the Skill Academy app on Google Play, and after basic filtering, 2993 valid reviews remained for analysis. Figure 2 shows the distribution of raw star ratings (1–5) along with their categorization into Negative, Neutral, and Positive. Most users gave high ratings (4–5 stars), but the proportion of 1-star ratings was also quite significant, indicating the presence of a group of users with unsatisfactory experiences.

### 3.2 Sentiment distribution based on VADER labeling results

**Figure 3.**
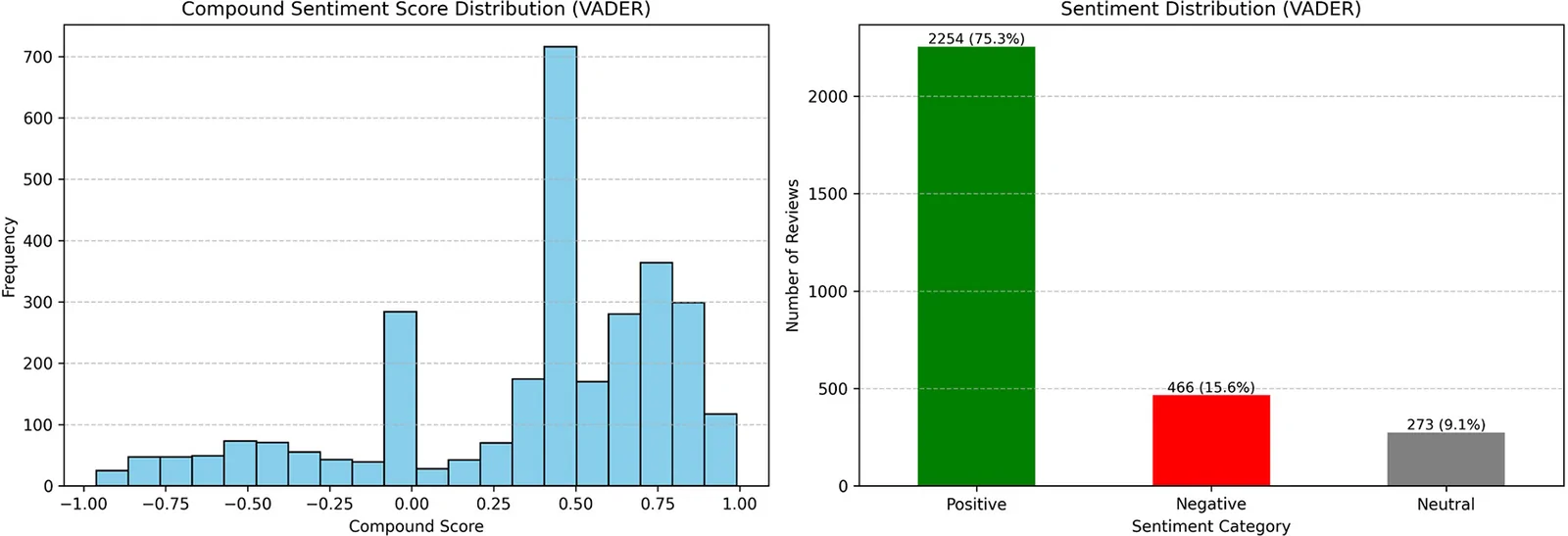
Distribution of the number of reviews by VADER sentiment class (left) and distribution of compound scores (right).

After the translation and labeling process using VADER, the sentiment distribution showed that the Positive class dominated with 2254 reviews (75.31%), followed by the Negative class with 466 reviews (15.57%), and the Neutral class with 273 reviews (9.12%), as shown in Figure 3. This trend is consistent with the empirical star-rating distribution shown in Figure 2, where the majority of users have had a positive experience with the Skill Academy app, although there remains a significant proportion of complaints.

The analysis of the continuous value distribution (compound score) in Figure 3 (left) provides a more specific picture of the depth of users’ emotions. The highest frequency peak is in the 0.4 to 0.5 score range, with over 700 reviews. A fairly high secondary frequency accumulation in the 0.6 to 0.9 score range reflects a group of users with high levels of satisfaction. Conversely, there is a sharp local peak at exact values representing objective reviews or text in which positive and negative sentiment cancel each other out in the lexical calculation. On the negative spectrum (<0.05), review frequencies are evenly distributed and relatively low, ranging from −0.25 to close to −1.00. This even distribution indicates that the intensity of user dissatisfaction varies widely, ranging from mild complaints to very strong dissatisfaction.

### 3.3 Cross validation of sentiment labels

The reliability and construct validity of the automatic labeling method were cross-validated through two validation stages: a cross-lexicon comparison between VADER and TextBlob, and a comparison with an empirical ground-truth proxy between VADER and Star Rating.

**Figure 4.**
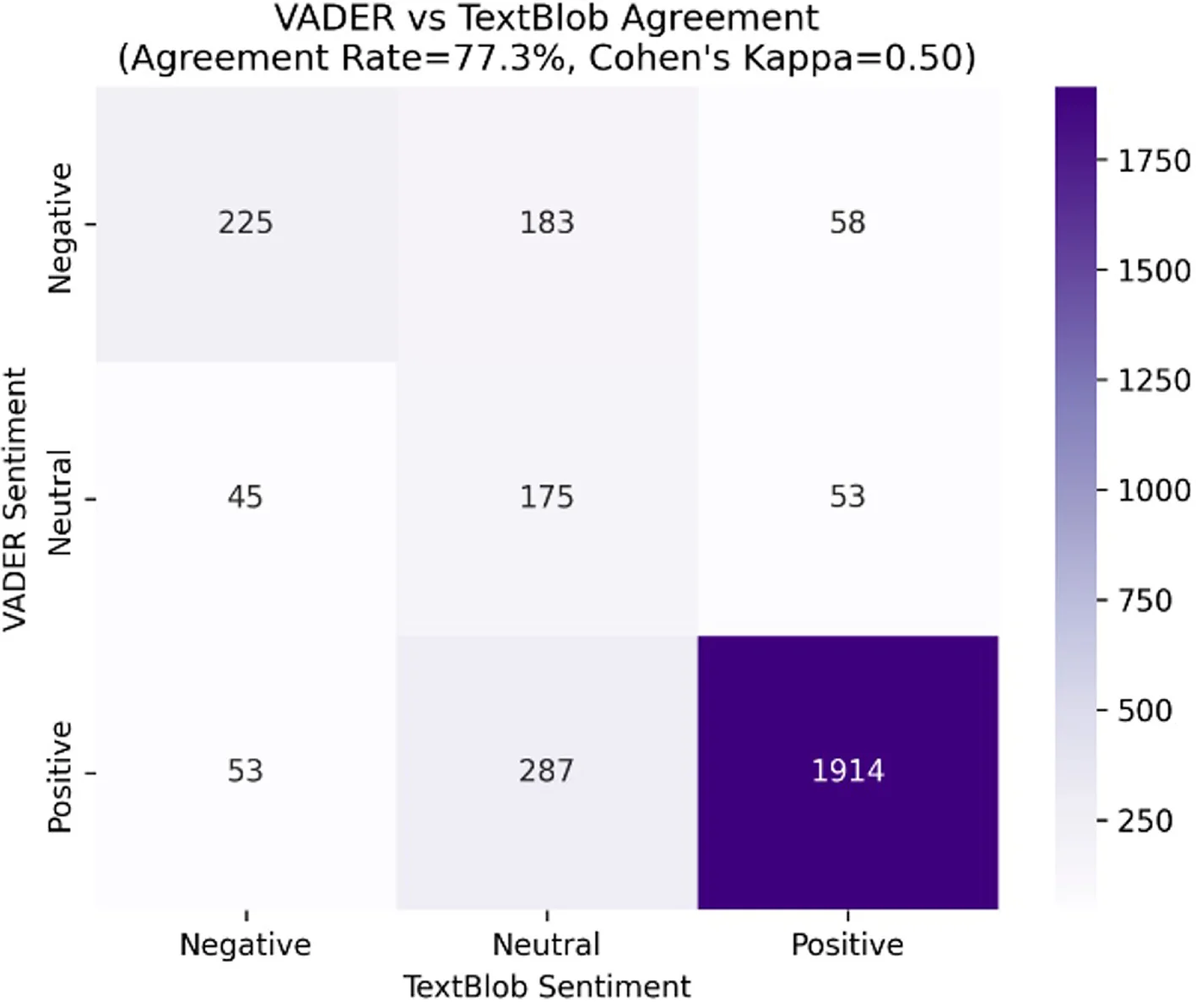
Heatmap of the VADER vs. TextBlob Sentiment Label Cross-Table.

The first validation showed an agreement rate of 77.31% between the VADER and TextBlob labels, with a Cohen’s kappa value of 0.50. This value indicates “moderate agreement” based on the criteria established by Landis and Koch (1977). The classification differences shown in Figure 4 are due to the operational characteristics of the two lexicons. VADER is specifically optimized for social media text and informal language, taking into account emotional intensity such as capitalization and punctuation, whereas TextBlob is based on standard word polarity, which tends to be more rigid in detecting informal or non-standard language expressions in translations.

**Figure 5.**
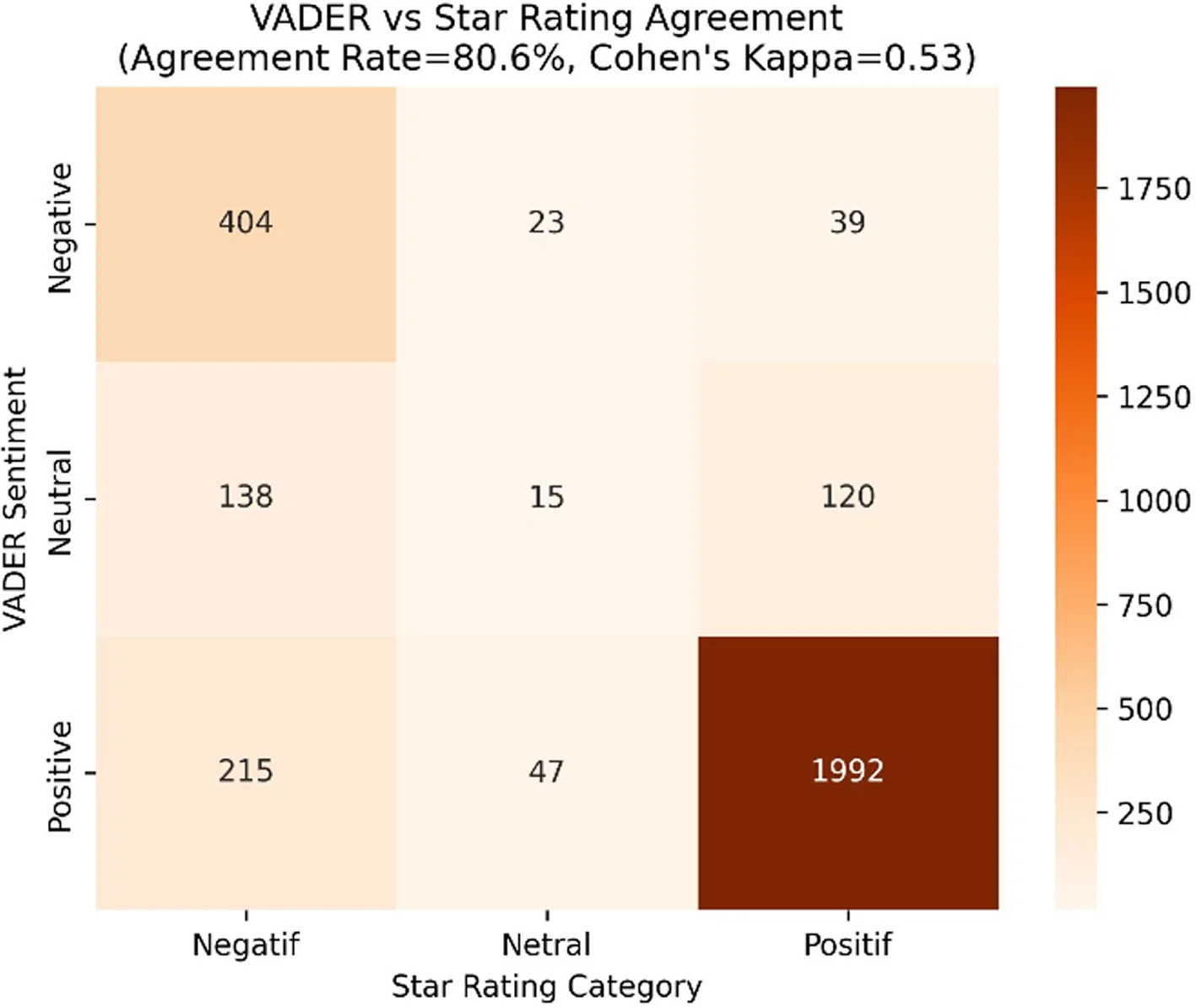
Heatmap of a cross-tabulation table showing VADER sentiment labels versus star rating categories.

The second validation compared VADER labels with users’ original star ratings and yielded a higher agreement rate of 80.55%, with Cohen’s kappa = 0.533, falling within the moderate agreement category (see Figure 5). This strong consistency demonstrates that automatic labeling using VADER is capable of representing the actual sentiment and self-reported satisfaction of platform users.

**Figure 6.**
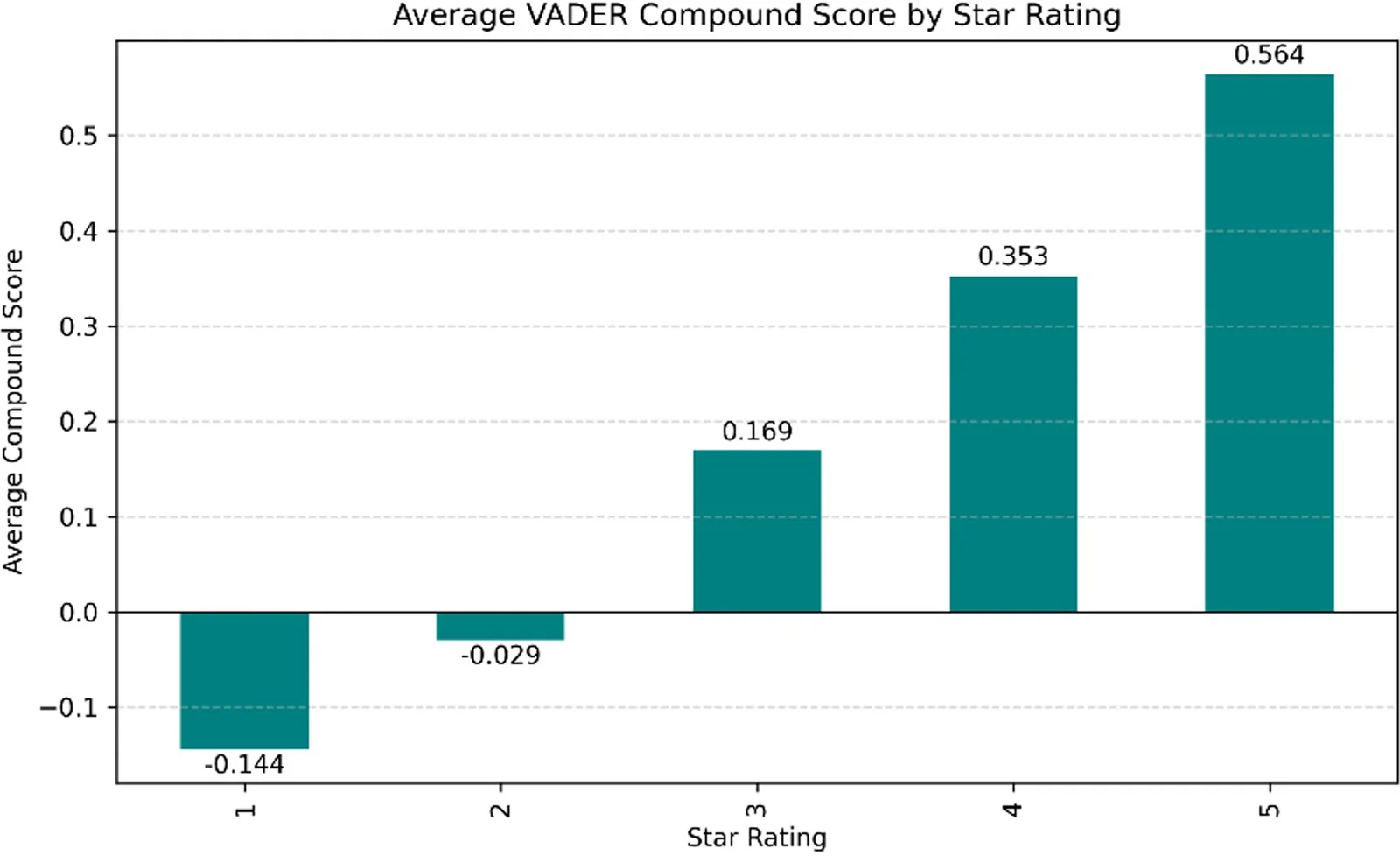
VADER’s average compound score based on star ratings.

An analysis of the average VADER compound score for each star rating level (1–5) is presented in Figure 6 as further confirmation. The visualization results show an ideal linear monotonic pattern, in which the average compound score increases consistently as the star rating rises, moving from negative values at a rating of 1 to high positive values at a rating of 5. This linear trend reinforces the construct validity of using the VADER lexicon to accurately measure user perceptions within the digital TVET ecosystem.

### 3.4 Keyword analysis by sentiment class

**Figure 7.**
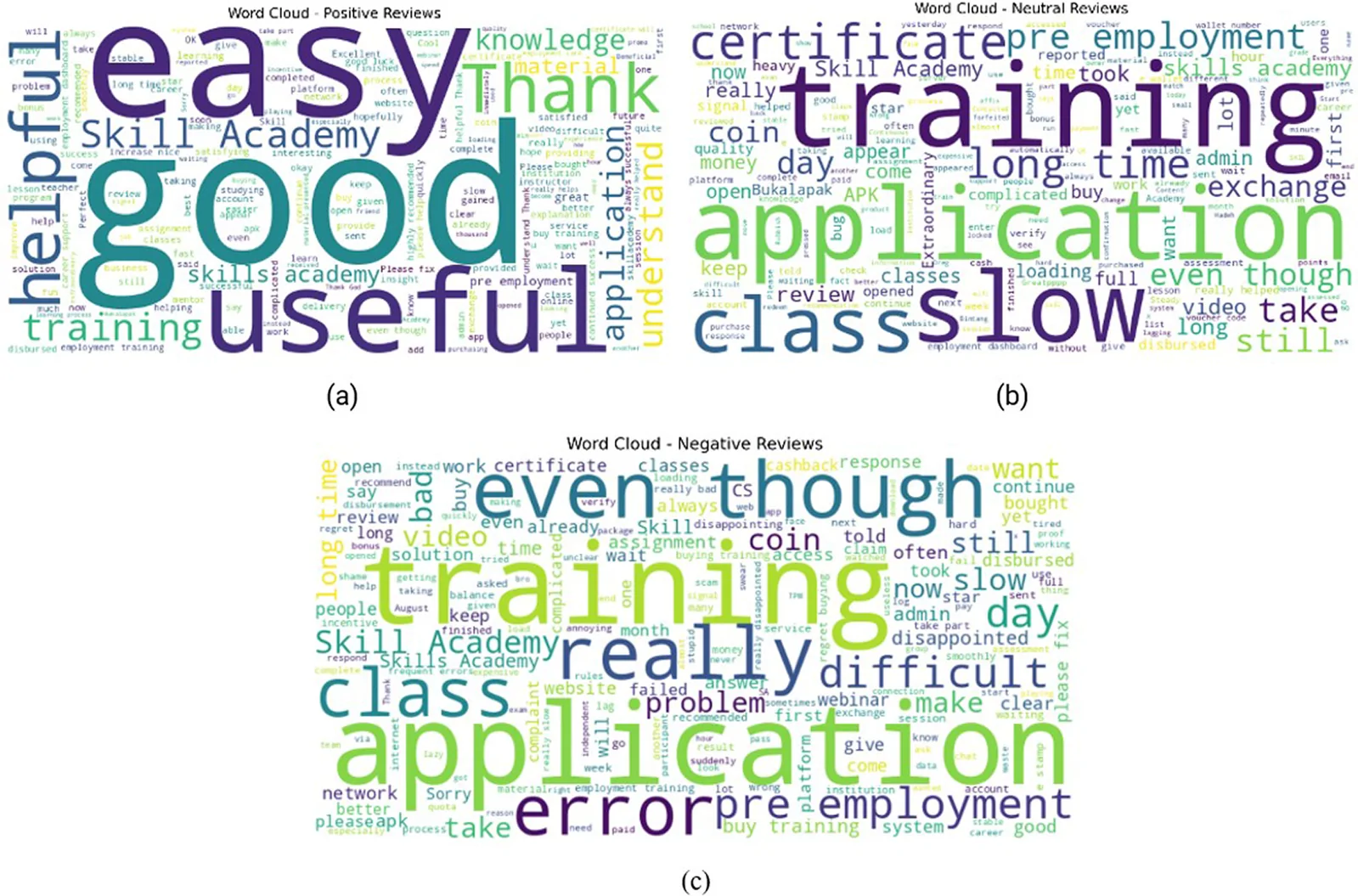
Word cloud of reviews: (a) Positive; (b) Negative; (c) Neutral.

**Figure 8.**
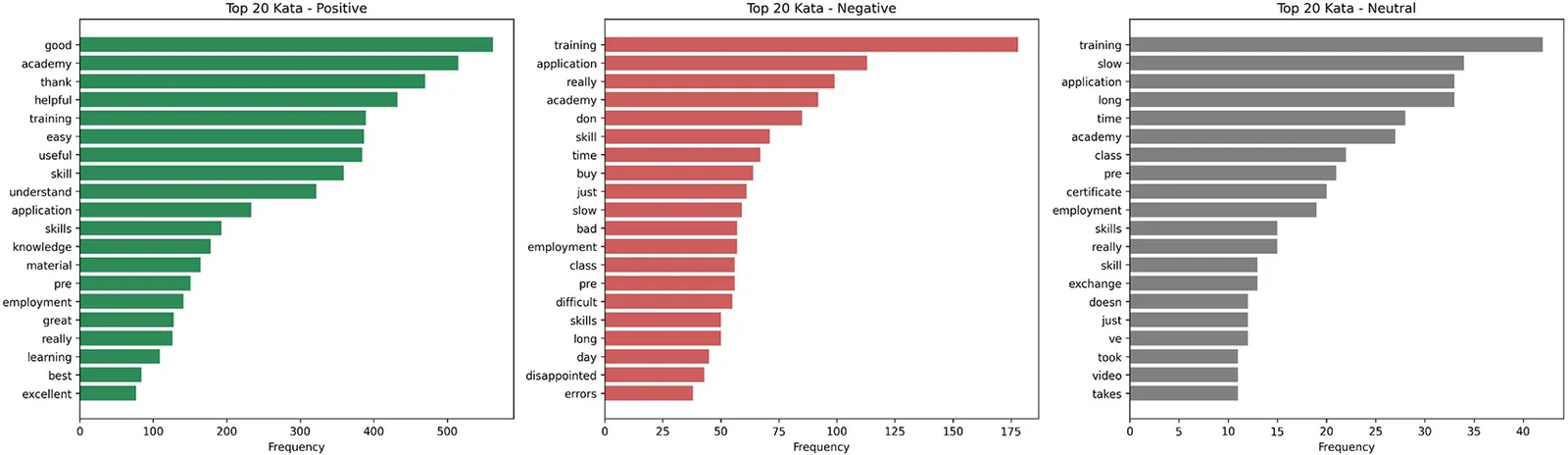
Top 20 words (unigrams) with the highest frequency per sentiment class.

**Figure 9.**
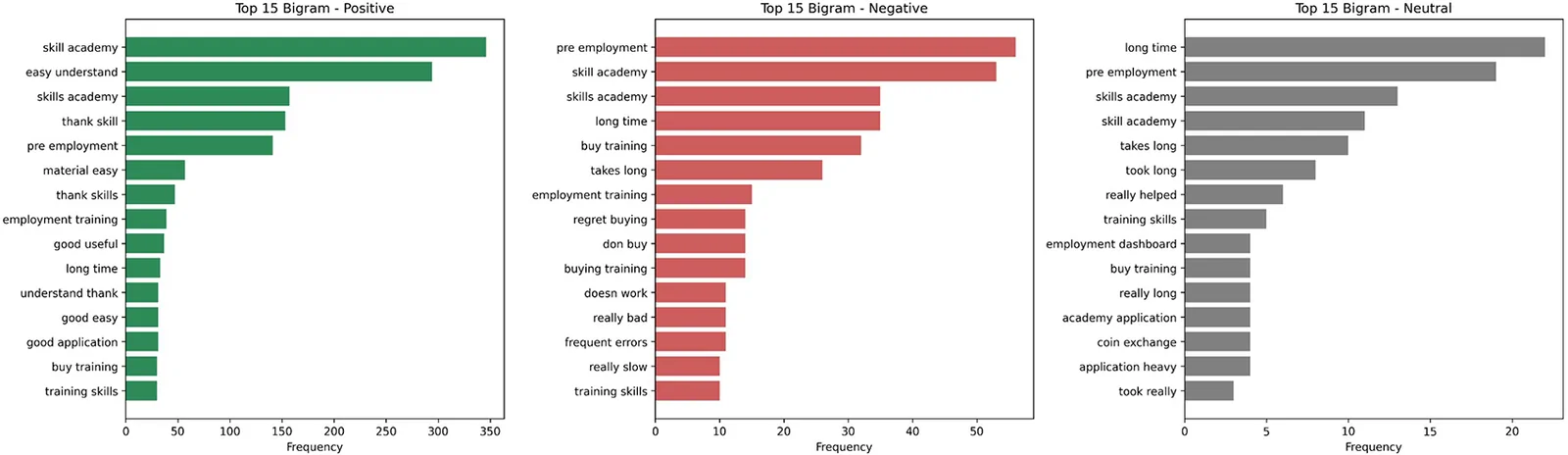
The top 15 two-word phrases (bigrams) with the highest frequency per sentiment class.

The linguistic characteristics of the translation output for each sentiment category were identified through the word cloud visualization in Figure 7, then quantitatively confirmed through the frequency of the top 20 unigrams in Figure 8 and the top 15 bigrams in Figure 9. In the positive cluster, the dominance of single words such as “good,” “helpful,” “easy,” and “useful” supported by strong phrases like “easy to understand” and “easy material” demonstrates the platform’s instructional quality. In the context of online TVET, the high frequency of these phrases indicates that the delivery of work competency materials via video and digital modules is considered interactive, clear, and capable of facilitating learners’ independent upskilling needs without the physical assistance of an instructor.

Far more critical operational dynamics within TVET were revealed in the negative and neutral sentiment clusters, where structural issues with government incentive programs took center stage. Negative unigram and bigram data overwhelmingly highlighted the phrase “pre-employment” as the top occurrence (approaching a frequency of 60 in bigrams), followed by the words “slow,” “long time,” “difficult,” “errors,” as well as the phrases “takes long,” “regret buying,” “really bad,” and “frequent errors.” These empirical findings indicate that learners’ deep dissatisfaction stems not from the substance of the vocational curriculum, but rather from systemic procedural barriers. Users face significant obstacles related to system processing times, ranging from the length of time required for post-training data synchronization and slow coin or voucher redemption processes to technical disruptions while completing classes.

The close connection between technical and bureaucratic obstacles and this government program is reinforced by the patterns in the neutral-class reviews. The neutral unigram and bigram graphs are absolutely dominated by the phrases “long time” (with the highest frequency exceeding 20), “pre-employment,” “takes long,” “took long,” and “coin exchange.” The presence of neutral clusters with these word patterns represents a group of learners providing objective reports on the waiting time for the system’s administrative processes. They neither criticize nor praise the learning content but simply note that transactions or class validations take an unreasonably long time to complete. For nationwide digital TVET governance, the comparative findings from these three keyword clusters confirm that the effectiveness of online vocational education cannot rely solely on high-quality infographics or videos; rather, it must be supported by the integration of digital infrastructure that is responsive, stable, and free from systemic bureaucratic delays.

### 3.5 Distribution of review length (Number of words) by sentiment class

**Figure 10.**
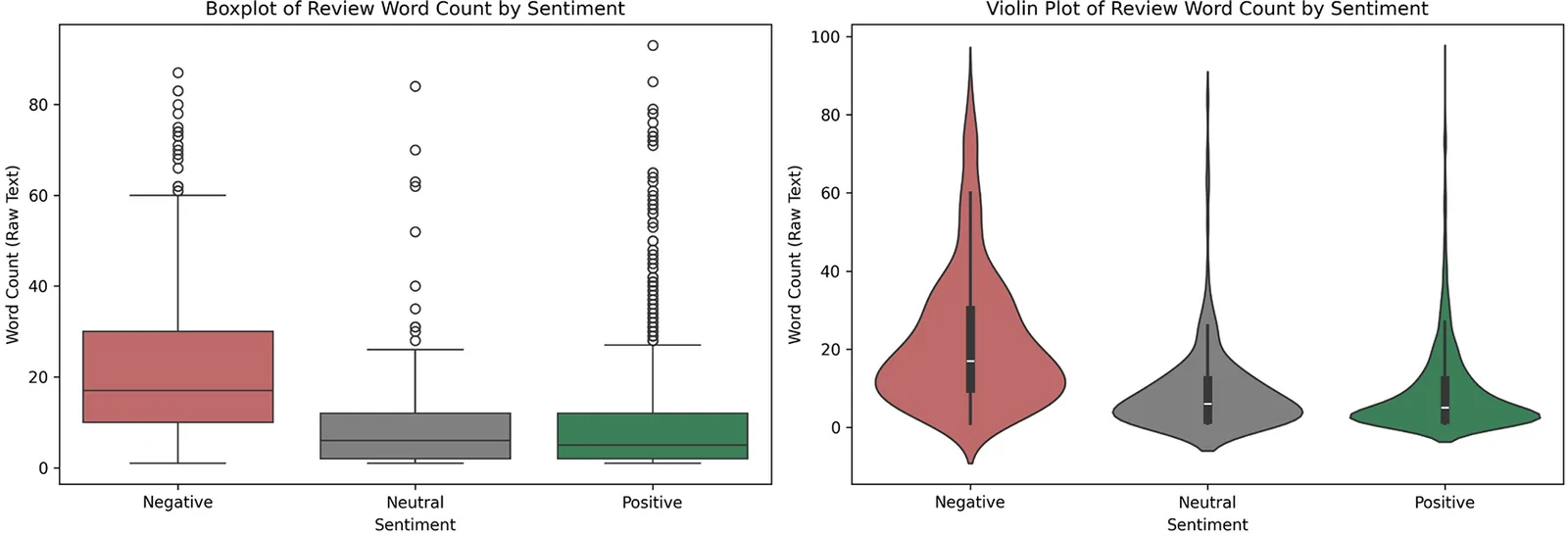
Distribution of review length (number of words) by sentiment class: boxplot (left) and violin plot (right).

The structural characteristics of user reviews show significant differences in text length (number of words) across sentiment classes, as summarized in Table 2 and visualized in Figure 10. Negative reviews had the highest word count, with a mean of 22.12 words and a standard deviation of 17.47 words. These values are more than double those of both Neutral reviews (mean = 9.01; SD = 10.76) and Positive reviews (mean = 9.04; SD = 11.13). The distribution of values across quartiles further underscores this polarization, with the median length of negative reviews reaching 17 words, while neutral and positive reviews range only between 5 and 6 words.

**Table 2.** Review length statistics (Word count) by sentiment class.

Sentiment	N	Mean	Std	Min	Q1 (25%)	Median	Q3 (75%)	Max
Negative	466	22.12	17.47	1	10	17	30	87
Neutral	273	9.01	10.76	1	2	6	12	84
Positive	2.254	9.04	11.13	1	2	5	12	93

This structural asymmetry reflects fundamental differences in users’ psychological behavior and motivations when providing feedback within the digital TVET ecosystem. Users who provide positive reviews tend to express their appreciation briefly and concisely using standard phrases that focus directly on the effectiveness of learning. In contrast, the high average word count and high variability in negative reviews indicate that users require more descriptive, chronological, and detailed narratives when facing operational issues. In the context of the Skill Academy platform, which is integrated with the Kartu Prakerja (pre-employment) program, these lengthy reviews generally contain chronological details of failed transactions, technical obstacles during the coin redemption process, or procedural complaints regarding delays in data synchronization that hinder their financial incentives.

**Figure 11.**
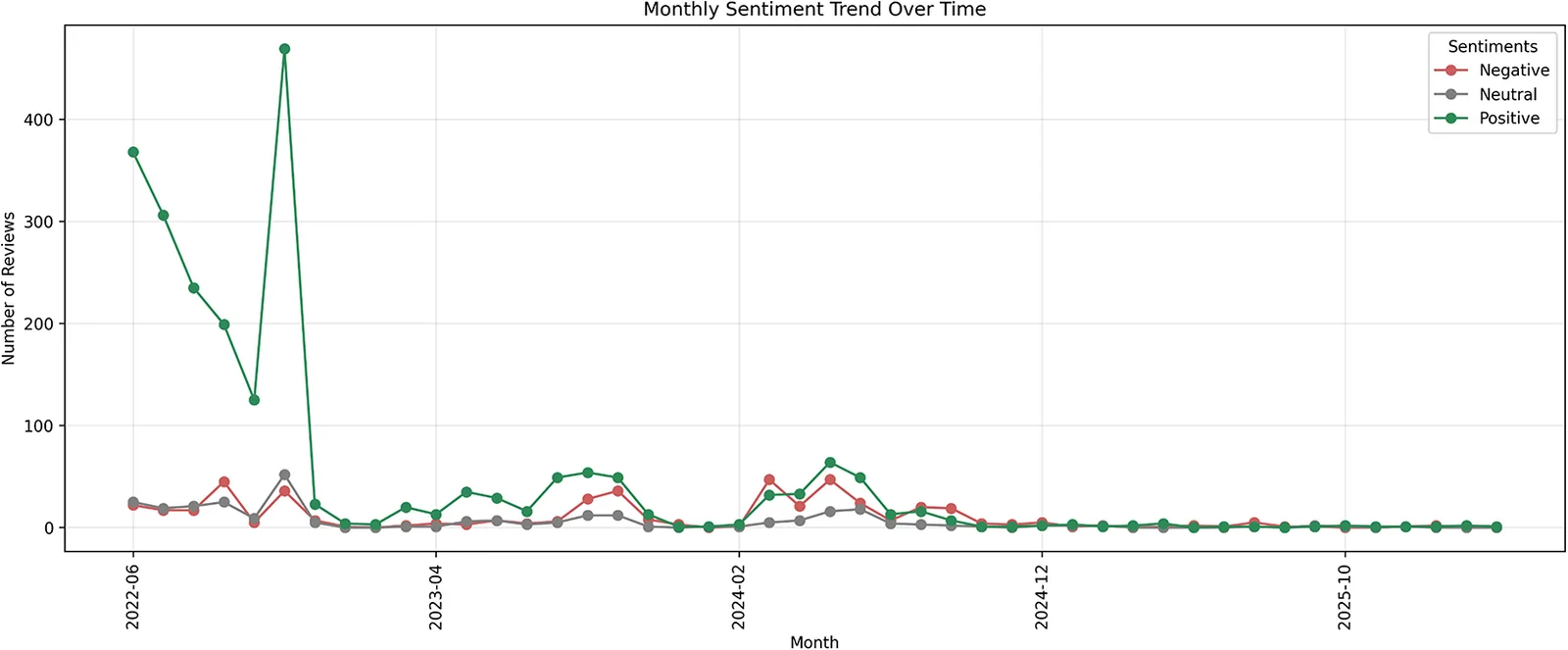
Trend in the number of reviews by sentiment category by month.

**Figure 12.**
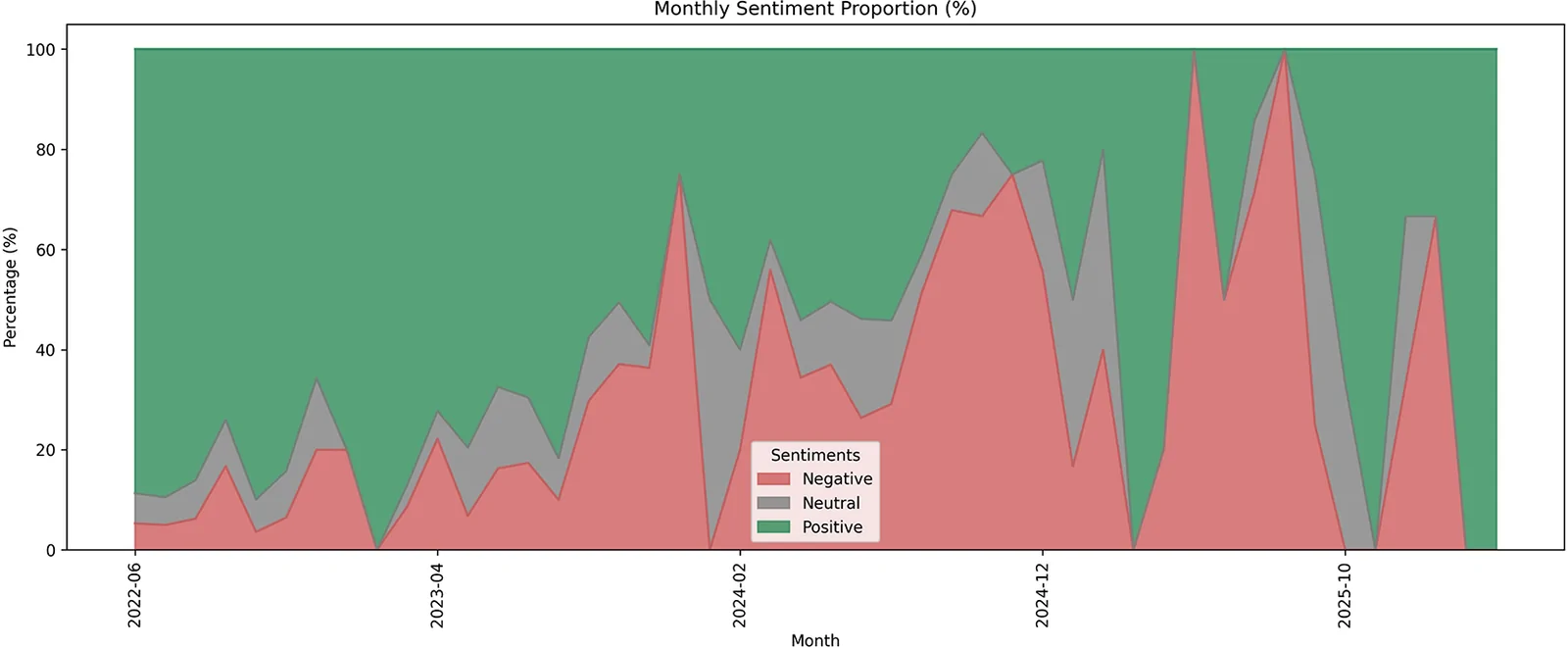
Trend in the percentage (%) of reviews by sentiment category by month.

A temporal analysis of the monthly sentiment trends presented in Figures 11 and Figure 12 shows that the proportion of positive sentiment remained consistently dominant throughout the observation period. This phenomenon demonstrates consistent and sustained acceptance of the platform’s vocational curriculum content. However, fluctuations specifically spikes in the volume of negative reviews during certain months indicate periods of specific operational disruptions. These fluctuating patterns represent temporary crises typically triggered by app updates or massive spikes in user traffic during the closing phase of training sessions that exceed server capacity. The integration of continuous word-length analysis with these temporal trends underscores the importance of regularly monitoring system performance to maintain the stability of nationwide digital TVET services.

### 3.6 Data preparation and training-test data split

After the labeling stage, a total of 2.993 reviews were used as the research dataset. The dataset was then divided into training and test data in an 80:20 ratio. The division was performed using a stratified sampling method based on sentiment classes to maintain the proportion of each class in both data subsets. A total of 2.394 reviews (80%) were allocated as training data, while 599 reviews (20%) were used as test data. The distribution of sentiment classes in each subset is presented in Table 3.

**Table 3.** Class distribution in the training and test datasets.

Sentiment class	Training data (n)	Training data (%)	Test data (n)	Test data (%)
Positive	1.803	75.31	451	75.29
Negative	373	15.58	93	15.53
Neutral	218	9.11	55	9.18
Total	2.394	100	599	100

**Figure 13.**
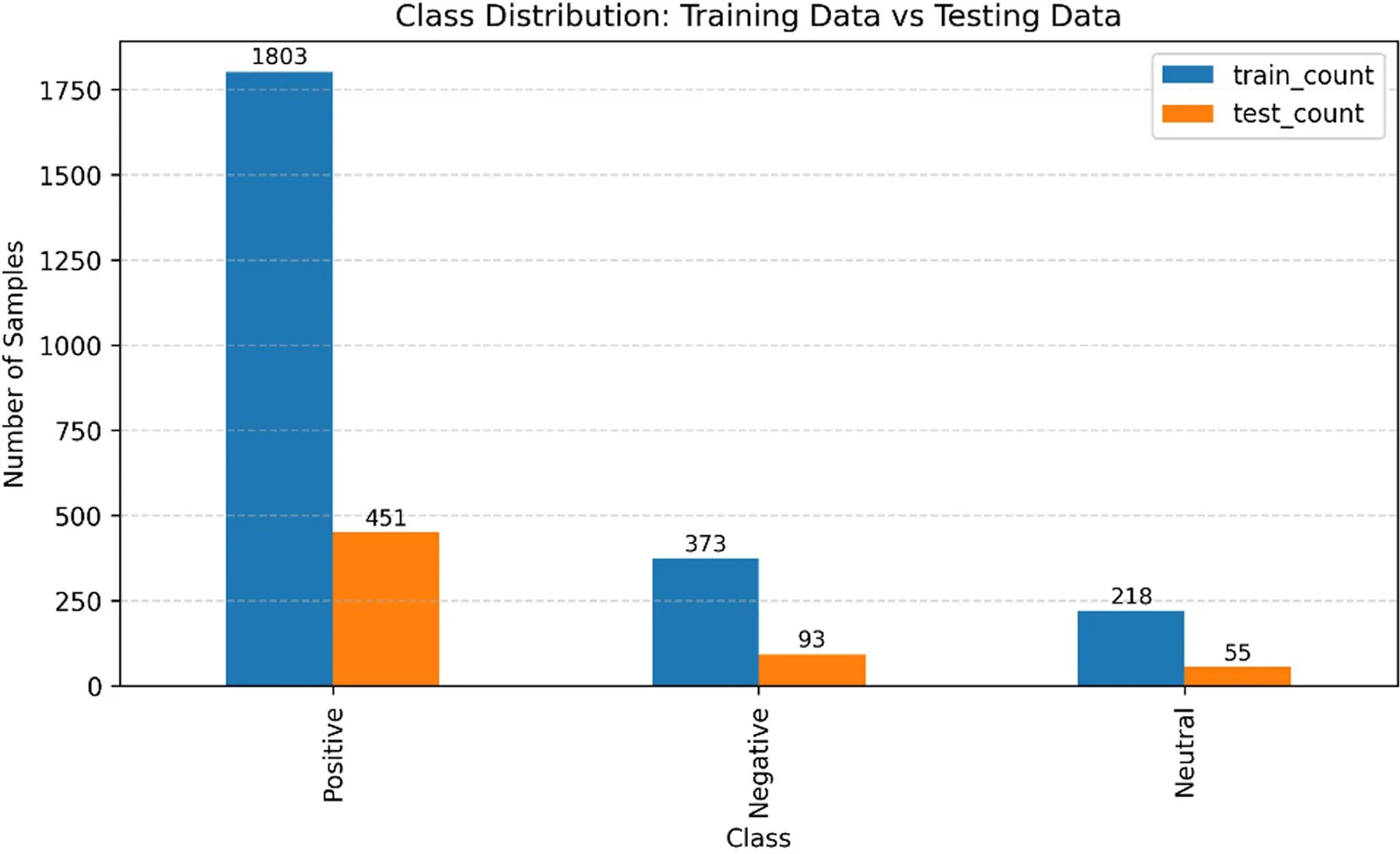
Comparison of class distributions in the training and test data.

Based on Table 3 and Figure 13, the class distributions in the training and test data show patterns that are relatively consistent with the distribution of the dataset as a whole. The Positive class is the most dominant, accounting for 75.31% of the training data and 75.29% of the test data. Meanwhile, the Negative class accounts for 15.58% and 15.53%, respectively, while the Neutral class has the lowest proportion, at 9.11% in the training data and 9.18% in the test data. The differences in proportions between subsets are less than 0.1 percentage points for all classes. These findings indicate that the application of stratified data splitting successfully maintained a proportional representation of each sentiment class. Thus, differences in class distribution between the training and test data can be minimized, ensuring that the model training and evaluation processes are conducted on comparable data distribution structures. Nevertheless, the distribution also reveals a class imbalance, as the majority of the data falls into the Positive class. This condition must be taken into account during the model evaluation stage, particularly since relying solely on accuracy metrics has the potential to produce a biased interpretation of performance favoring the majority class. Therefore, model evaluation was conducted using class-based metrics, including precision, recall, F1-score, and the confusion matrix.

**Figure 14.**
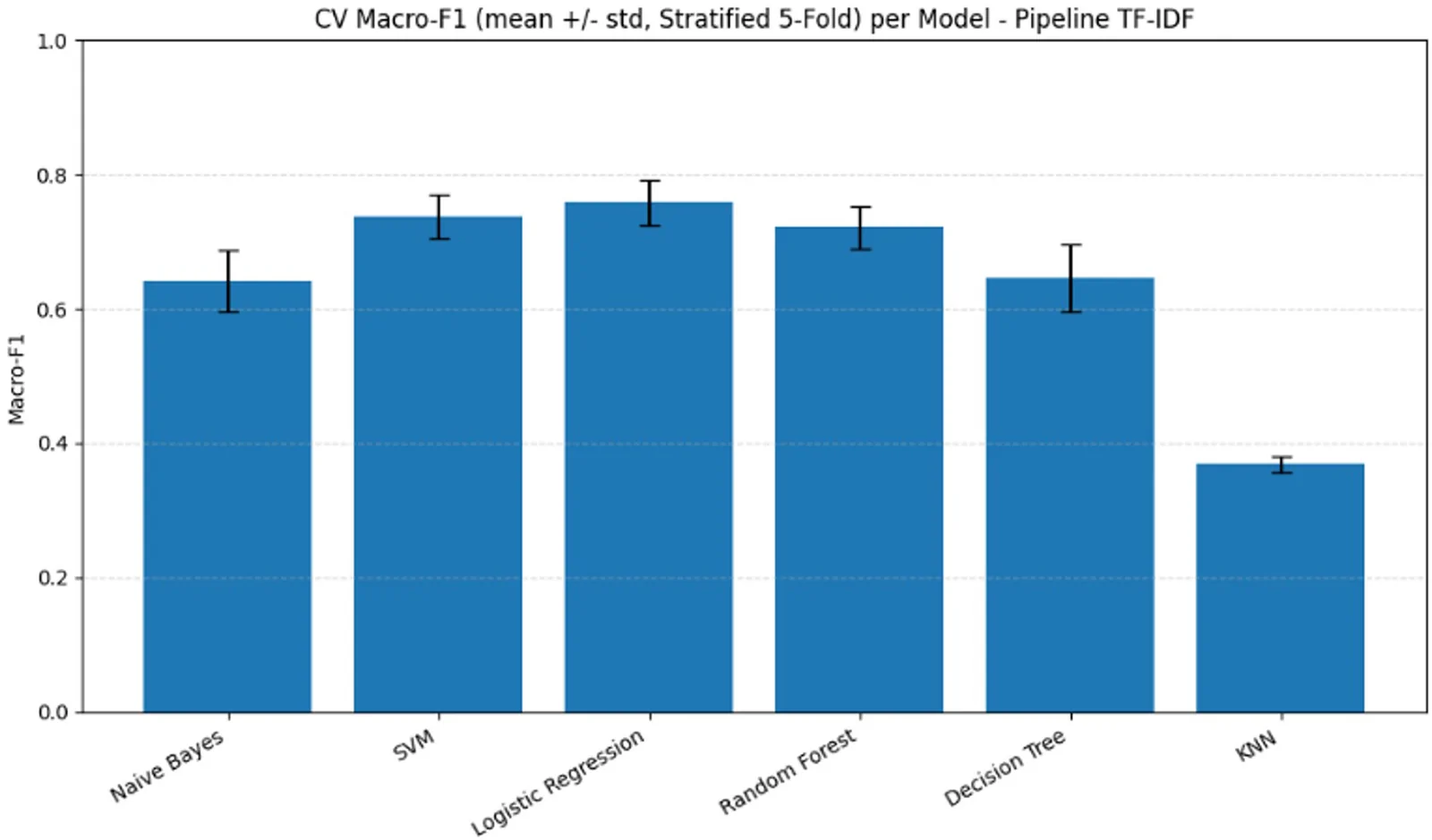
Comparison of cross-validation macro-F1 scores across models.

### 3.7 Hyperparameter tuning results (Cross-validation)

Hyperparameter optimization using a five-fold cross-validation scheme resulted in significant variations in performance among the eight classification model architectures, as summarized in Table 4. Evaluation based on the macro-F1 metric shows that Logistic Regression achieved the highest performance with an average cross-validation (CV) score of 0.7581 ± 0.0330. This result outperformed both ensemble-based and complex kernel models, such as XGBoost (0.7387 ± 0.0340) and Support Vector Machine (SVM) (0.7377 ± 0.0327). In contrast, K-Nearest Neighbors (KNN) recorded the lowest efficiency, with an average macro-F1 score that dropped sharply to 0.3683 ± 0.0126.

**Table 4.** Summary of best hyperparameters and cross-validation macro-F1 scores.

Model	Hyperparameter	CV Macro-F1 (Mean ± SD)
Logistic Regression	C = 10; class_weight = balanced	0.7581 ± 0.0330
XGBoost	learning_rate = 0.1; max_depth = 6; n_estimators = 200	0.7387 ± 0.0340
SVM	C = 10; kernel = linear	0.7377 ± 0.0327
LightGBM	learning_rate = 0.05; n_estimators = 200; num_leaves = 15	0.7042 ± 0.0208
Random Forest	class_weight = balanced; max_depth = None; n_estimators = 200	0.7216 ± 0.0315
Decision Tree	class_weight = balanced; max_depth = 40	0.6463 ± 0.0507
Naive Bayes	alpha = 0.1	0.6419 ± 0.0462
KNN	n_neighbors = 3	0.3683 ± 0.0126

The superior performance of Logistic Regression is supported by an optimal hyperparameter configuration with a regularization value of C = 10 and the application of the class_weight = balanced parameter. This class weight tuning plays a crucial role in mitigating the effects of extreme class imbalance in the Skill Academy review dataset. Through inverse proportional weighting based on class frequency, the linear estimator is able to construct an objective decision boundary without being biased by the dominance of the majority class. Furthermore, the characteristics of text data represented as a high-dimensional sparse matrix—are well-suited to the regularized linear loss function, making this model more effective than single decision tree algorithms such as Decision Tree (0.6463 ± 0.0507) or the probabilistic Naive Bayes model (0.6419 ± 0.0462).

The poor performance of KNN with the configuration n_neighbors = 3 is due to the inherent limitations of distance-metric-based algorithms in high-dimensional vector spaces. In sparse unigram and bigram text feature representations, the calculation of Euclidean distances between documents loses its geometric differentiation because the distances between data points tend to become uniform. The absence of a balancing class weighting function in KNN exacerbates misclassification in minority classes (negative and neutral). On the other hand, gradient-boosting models such as XGBoost and LightGBM (0.7042 ± 0.0208) demonstrate good resilience through iterative optimization of the loss function, although their parameter combinations have not yet been able to match the computational efficiency and generalization accuracy of the linear decision boundary produced by Logistic Regression.

The interaction between hyperparameter tuning and model architecture has strategic implications for the implementation of an automated monitoring system on a digital TVET platform. The stability of low standard deviation values across high-scoring models demonstrates a reliable level of generalization. The use of Logistic Regression as the selected model ensures the detection of minority sentiments. This balanced classification accuracy validates the model’s readiness to be deployed as an automated text analytics module to support the continuous evaluation and improvement of online training service quality.

### 3.8 Model performance evaluation on test data (Holdout)

This Model Accuracy Comparison graph shows a comparison of the accuracy levels of Table 5 presents the complete evaluation results for the eight models on test data that was not used at all in the tuning process. Logistic Regression delivered the best overall performance based on macro-F1 (0.7608) and balanced accuracy (0.8086), followed by SVM (macro-F1 = 0.7596), which had the highest accuracy (0.8631, tied with XGBoost). XGBoost recorded the highest recall for the Positive class (0.9224) but a lower recall for the Neutral class (0.5455) compared to Logistic Regression (0.6727). KNN consistently showed the lowest performance across all metrics, with a very low Negative class recall (0.0215), indicating that this model failed to adequately identify negative reviews despite its very high Neutral class recall (0.9636)—a pattern typical of models biased toward the majority class based on the proximity of high-dimensional TF-IDF features.

**Table 5.** Summary of model evaluation metrics on the test (Holdout) data.

Model	Accuracy	Macro-averaged F1-Score	Weighted-averaged F1-Score	Balanced accuracy	Macro-averaged precision	Macro-averaged recall	Recall for negative class	Recall for neutral class	Recall for positive class
Logistic Regression	0.8548	0.7608	0.8618	0.8086	0.7290	0.8086	0.8817	0.6727	0.8714
SVM	0.8631	0.7596	0.8649	0.7707	0.7508	0.7707	0.7849	0.6182	0.9091
XGBoost	0.8631	0.7440	0.8629	0.7438	0.7484	0.7438	0.7634	0.5455	0.9224
LightGBM	0.8314	0.7240	0.8420	0.7784	0.6895	0.7784	0.8065	0.6727	0.8559
Random Forest	0.8381	0.7095	0.8379	0.7082	0.7195	0.7082	0.7312	0.4909	0.9024
Naive Bayes	0.8214	0.6460	0.8187	0.6743	0.7026	0.6743	0.8925	0.2545	0.8758
Decision Tree	0.7830	0.6424	0.7972	0.6869	0.6190	0.6869	0.6022	0.6182	0.8404
KNN	0.5509	0.3599	0.5999	0.5316	0.7174	0.5316	0.0215	0.9636	0.6098

**Table 6.** Summary of macro and micro AUC (One-vs-Rest) by model.

Model	AUC macro (OvR)	AUC micro (OvR)
Logistic Regression	0,9486	0,9683
SVM	0,9436	0,9709
XGBoost	0,9383	0,9680
Random Forest	0,9306	0,9617
LightGBM	0,9277	0,9562
Naive Bayes	0,9233	0,9532
Decision Tree	0,7791	0,8762
KNN	0,7155	0,7025

**Figure 15.**
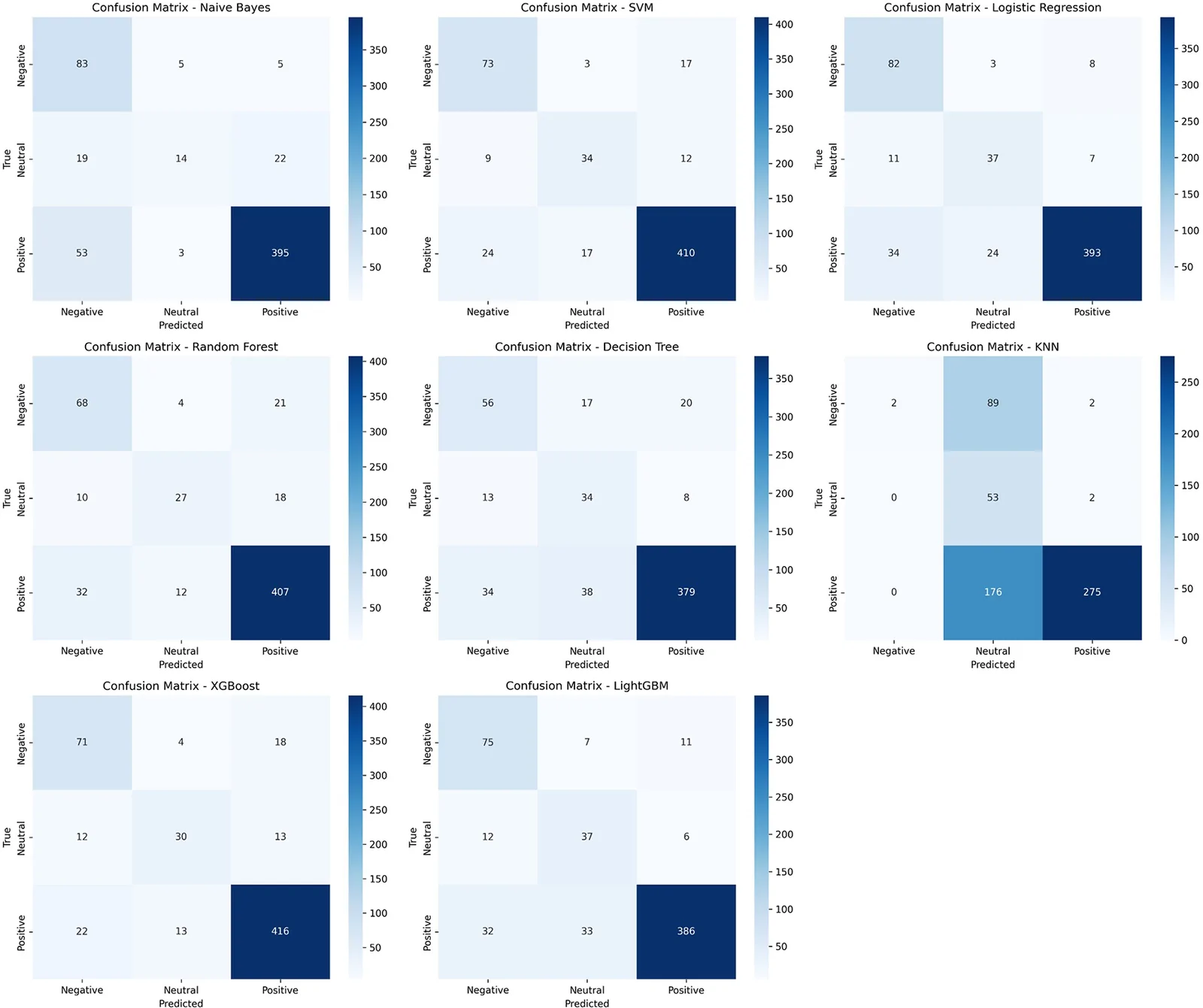
Confusion matrices for the eight models on the test data.

Figure 15 shows that Logistic Regression, SVM, and XGBoost produce the best confusion matrix patterns because most of the predictions are concentrated on the main diagonal. Logistic Regression correctly classified 82 Negative data points, 37 Neutral data points, and 393 Positive data points, with the largest errors occurring in 34 Positive data points predicted as Negative and 24 Positive data points predicted as Neutral. This pattern indicates that Logistic Regression is relatively capable of maintaining a balance in classification across classes, although it still faces difficulties when Positive reviews contain words or sentence structures that resemble those in Negative or Neutral reviews. SVM produced 73 correct predictions for the Negative class, 34 for Neutral, and 410 for Positive. The most notable errors in SVM were 24 positive data points predicted as negative and 17 negative data points predicted as positive, indicating that the cross-class misclassification between the negative and positive classes remains quite significant. XGBoost produced the highest number of correct predictions in the Positive class 416 data points but still classified 18 Negative and 13 Neutral data points as Positive. This demonstrates XGBoost’s tendency to more frequently choose the Positive class when a review’s characteristics lack sufficiently strong distinguishing features. LightGBM was able to correctly classify 75 Negative data points, 37 Neutral data points, and 386 Positive data points, but there were 32. XGBoost produced the highest number of correct predictions in the Positive class 416 data points but still classified 18 Negative and 13 Neutral data points as Positive. This demonstrates XGBoost’s tendency to more frequently select the Positive class when a review’s characteristics lack sufficiently strong distinguishing features. LightGBM was able to correctly classify 75 Negative data points, 37 Neutral data points, and 386 Positive data points, but there were 32 Positive data points predicted as Negative and 33 Positive data points predicted as Neutral. Compared to SVM and XGBoost, LightGBM appears to reclassify Positive data points into other classes more frequently, indicating that the decision boundary for the Positive class is still not sufficiently well-defined. Random Forest successfully classified 68 Negative data points, 27 Neutral data points, and 407 Positive data points; however, 21 Negative data points and 18 Neutral data points were predicted as Positive. This pattern indicates that Random Forest tends to favor the majority class, causing some data from other classes to be misclassified as Positive. The Decision Tree produced 56 correct predictions for the Negative class, 34 for Neutral, and 379 for Positive, but its errors were more widespread, particularly with 17 Negative data points predicted as Neutral, 20 Negative data points predicted as Positive, and 38 Positive data points predicted as Neutral. This wider error distribution indicates that the Decision Tree has not yet been able to form a stable decision boundary between classes. Naive Bayes was able to correctly classify 83 Negative data points and 395 Positive data points, but only 14 Neutral data points were successfully identified. A total of 19 Neutral data points were predicted as Negative and 22 others were predicted as Positive, indicating that the Neutral class shares overlapping vocabulary characteristics with both of those classes. KNN exhibited the most extreme error pattern, as only two Negative data points were classified correctly, while 89 Negative and 176 Positive data points were instead predicted as Neutral. This model produced a very large number of Neutral predictions 318 data points even though the actual number of Neutral class data points was only 55. This indicates that KNN exhibits a strong bias toward the Neutral class and fails to achieve effective separation in the high-dimensional TF-IDF feature space.

Based on the overall confusion matrix, the Neutral class is the most difficult for most models to consistently identify. This is evident from the Neutral class’s lower recall compared to the other two classes in Logistic Regression, SVM, XGBoost, Random Forest, LightGBM, and Naive Bayes. This difficulty may arise because neutral reviews often contain vocabulary that also appears in both positive and negative reviews, but without strong sentiment intensity. However, it is incorrect to conclude that errors are always more prevalent between the Neutral and Positive classes. In the three high-performing models, the number of two-way errors between the Negative and Positive classes was actually higher: 42 errors in Logistic Regression, 41 errors in SVM, and 40 errors in XGBoost. By comparison, the errors between Neutral and Positive totaled 31, 29, and 26 data points, respectively.

Overall, Logistic Regression provides the most balanced prediction pattern across classes, while SVM and XGBoost yield higher overall accuracy but are more biased toward the Positive class. XGBoost is the best model at identifying the Positive class, while Logistic Regression is best at maintaining balanced recall across all three classes. These findings confirm that determining the best model cannot be based solely on accuracy; it is necessary to consider macro-F1, balanced accuracy, recall for each class, and the type of classification error deemed most important for the research objectives.

**Figure 16.**
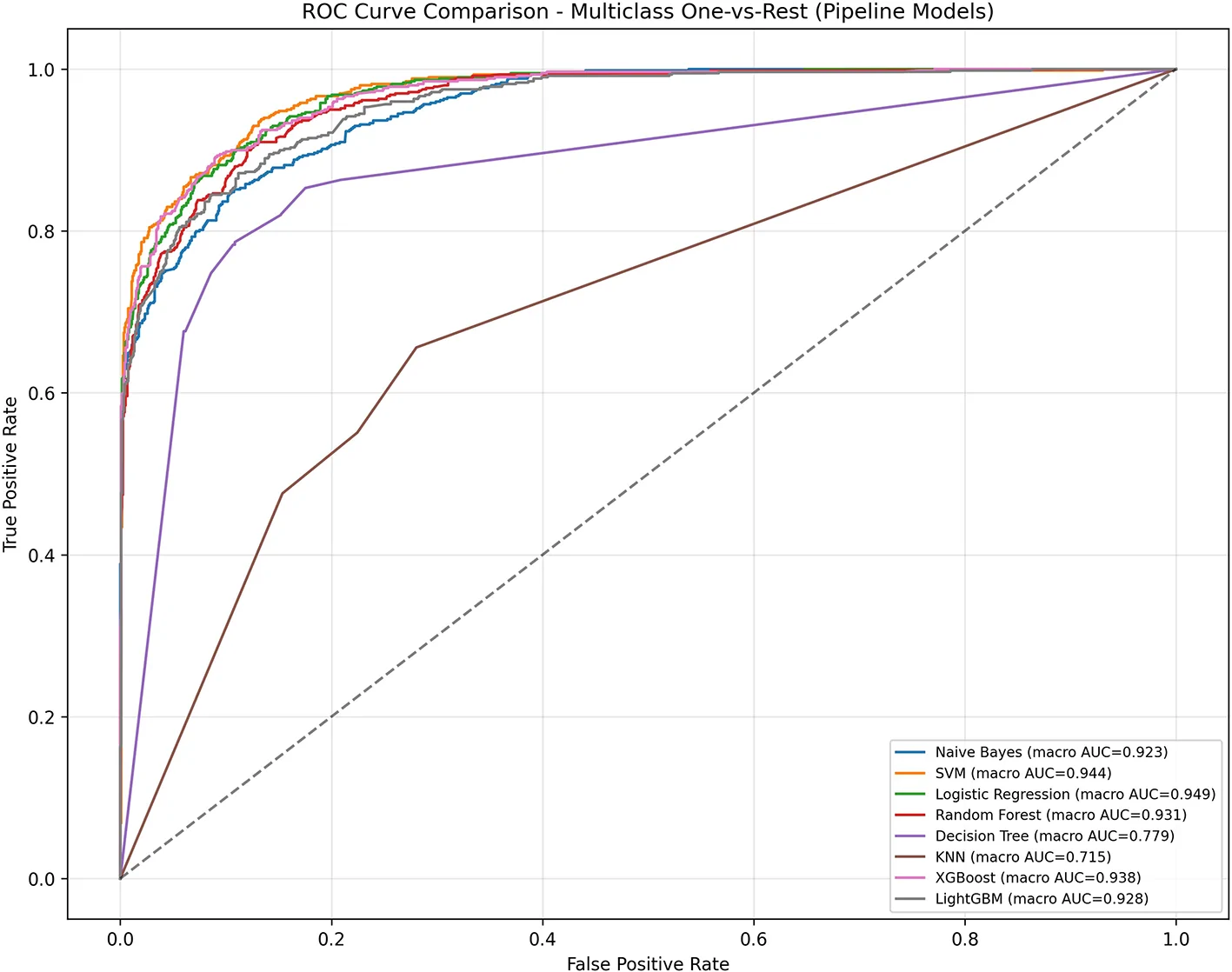
One-vs-Rest ROC curve for all models.

The ROC curves using the One-versus-Rest scheme in Figure 16 show that most models have good discriminatory power, as their curves lie well above the diagonal line. Logistic Regression achieved the highest macro AUC of 0.9486, followed by SVM at 0.9436 and XGBoost at 0.9383. These macro AUC values indicate that Logistic Regression is the most consistent in distinguishing the Negative, Neutral, and Positive classes from the combined set of other classes. On the other hand, SVM recorded the highest micro-AUC of 0.9709, slightly higher than Logistic Regression at 0.9683 and XGBoost at 0.9680, indicating the best aggregate discrimination ability across the entire sample. However, the micro AUC is more heavily influenced by the class with the largest amount of data, so its value should be interpreted in conjunction with the macro AUC. Random Forest, LightGBM, and Naive Bayes also demonstrate high discriminatory power with macro AUCs above 0.92, although the confusion matrix results show that their performance is not consistent across all classes. In contrast, Decision Tree and KNN produced the lowest macro AUCs, at 0.7791 and 0.7155, respectively, indicating weaker class separation capabilities. Overall, the ROC-AUC results reinforce that Logistic Regression has the best balance of discrimination across classes, while SVM excels in aggregate performance.

**Figure 17.**
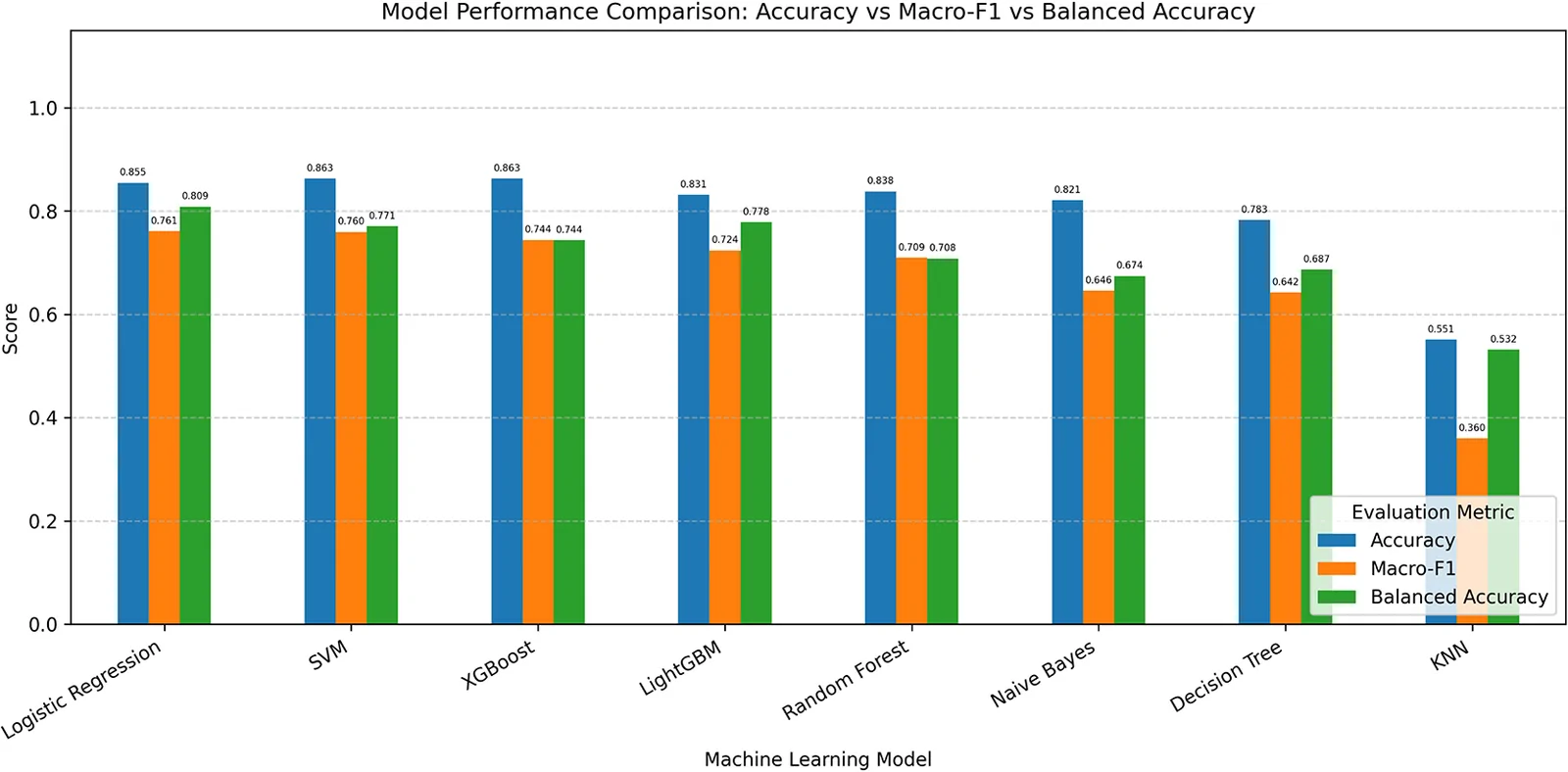
Comparison of accuracy, Macro-F1, and balanced accuracy across models.

**Figure 18.**
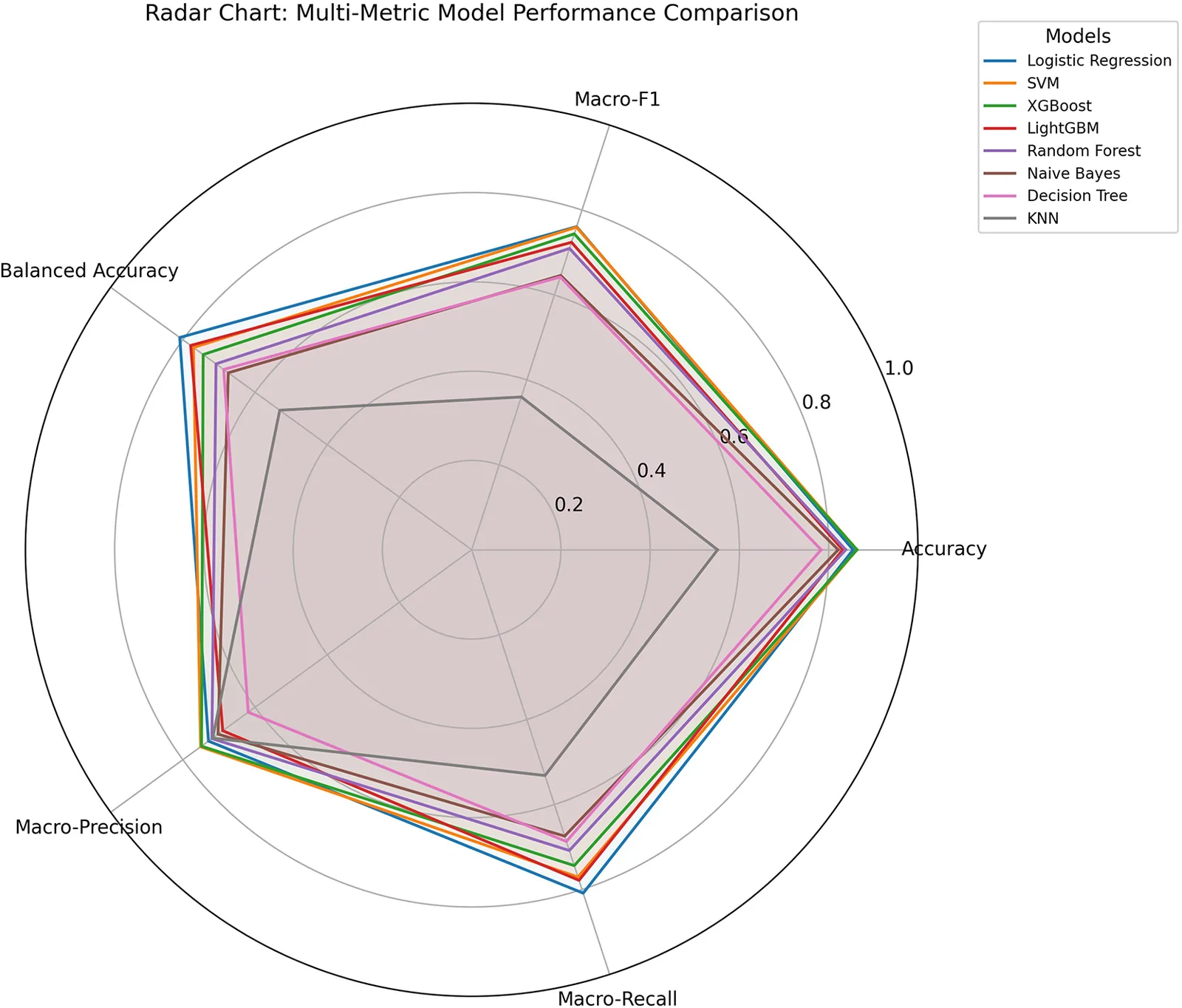
Radar chart comparing five evaluation metrics across models.

The visual comparison of accuracy, macro-F1, and balanced accuracy shown in Figure 17 underscores the importance of using multiple metrics on imbalanced data: models with high accuracy (SVM, XGBoost) do not always achieve balanced accuracy as high as Logistic Regression, because accuracy is naturally skewed by performance on the majority class (Positive). The radar chart in Figure 18 illustrates the strengths and weaknesses of each model across all five metrics (accuracy, macro-F1, balanced accuracy, macro-precision, and macro-recall), with Logistic Regression showing the most balanced profile across all five metric axes.

**Figure 19.**
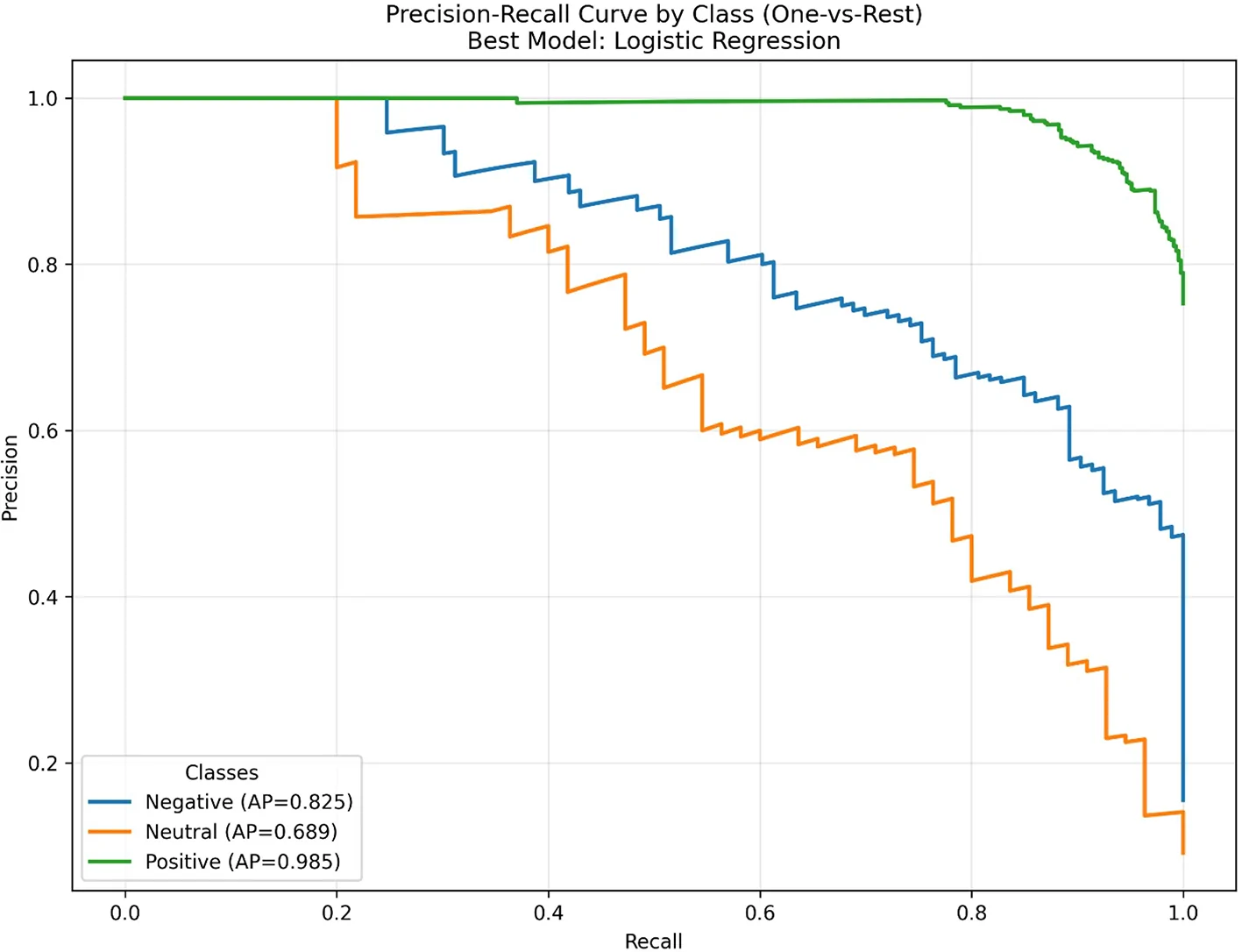
Precision-recall curves by class for the best model (Logistic Regression).

As the model with the highest macro-F1 score, Logistic Regression was further analyzed using precision-recall curves for each class ( Figure 19). The Positive class exhibited the best precision-recall curve (closest to the top-right corner), while the Neutral class showed the flattest curve, confirming that this minority class is the most difficult for the model to classify consistently.

**Figure 20.**
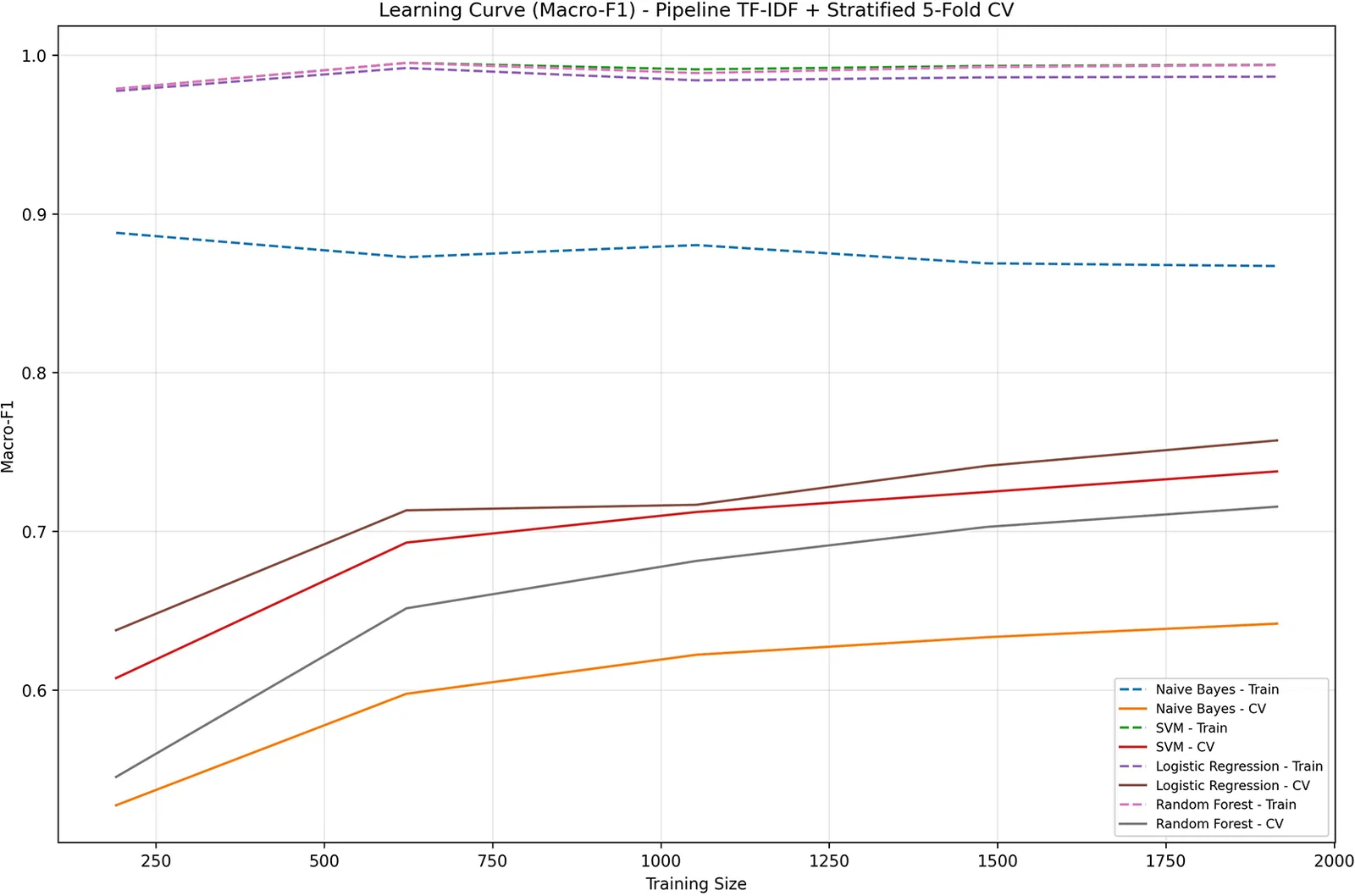
Learning curves for the four best-performing models.

The learning curve ( Figure 20) for the top four models shows that the cross-validation scores increase steadily as the training dataset size increases and begin to converge toward the training scores at the maximum dataset size, indicating that there is no severe overfitting, although a gap between the training and validation scores is still evident in more complex models such as Random Forest, suggesting that these models could potentially benefit from additional data.

**Figure 21.**
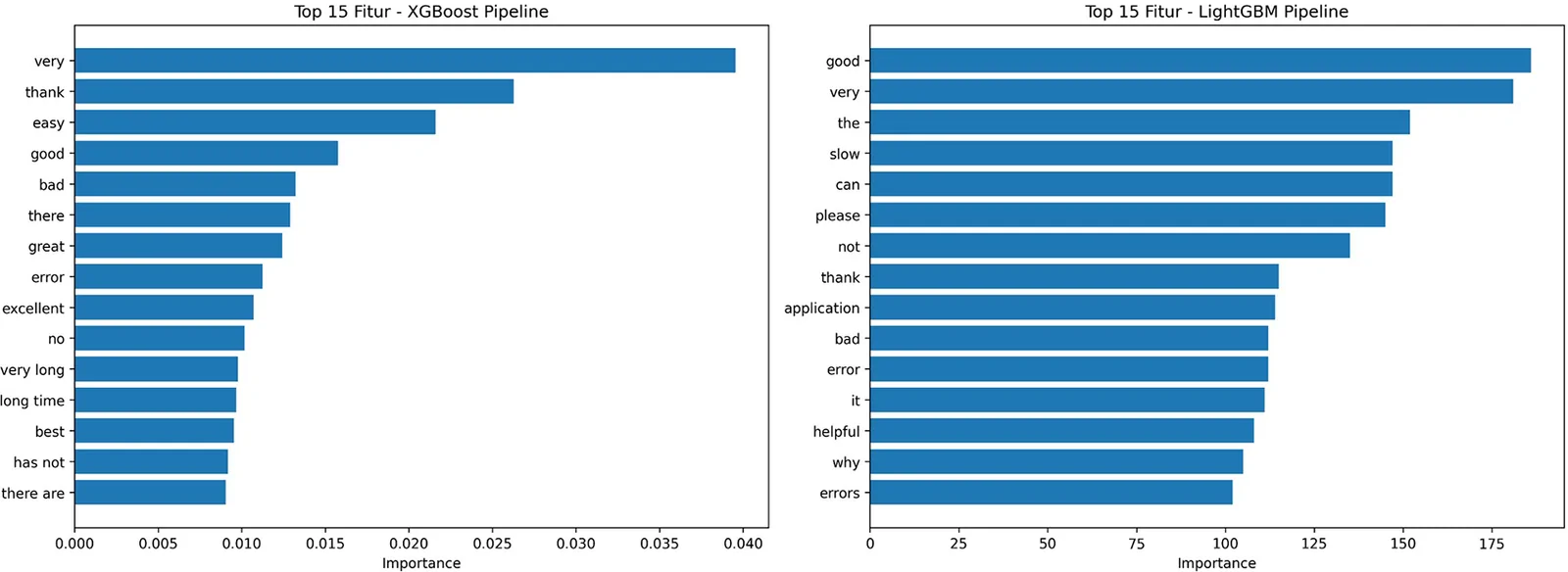
Top 15 feature importance models for XGBoost (left) and LightGBM (right).

The feature importance analysis of the tree-based models (XGBoost and LightGBM) shown in Figure 21 reveals that the features most influential on sentiment classification decisions are consistent with the findings of the unigram/bigram analysis, namely words related to application performance and the quality of learning materials.

### 3.9 Experiment in addressing class imbalance using SMOTETomek

**Figure 22.**
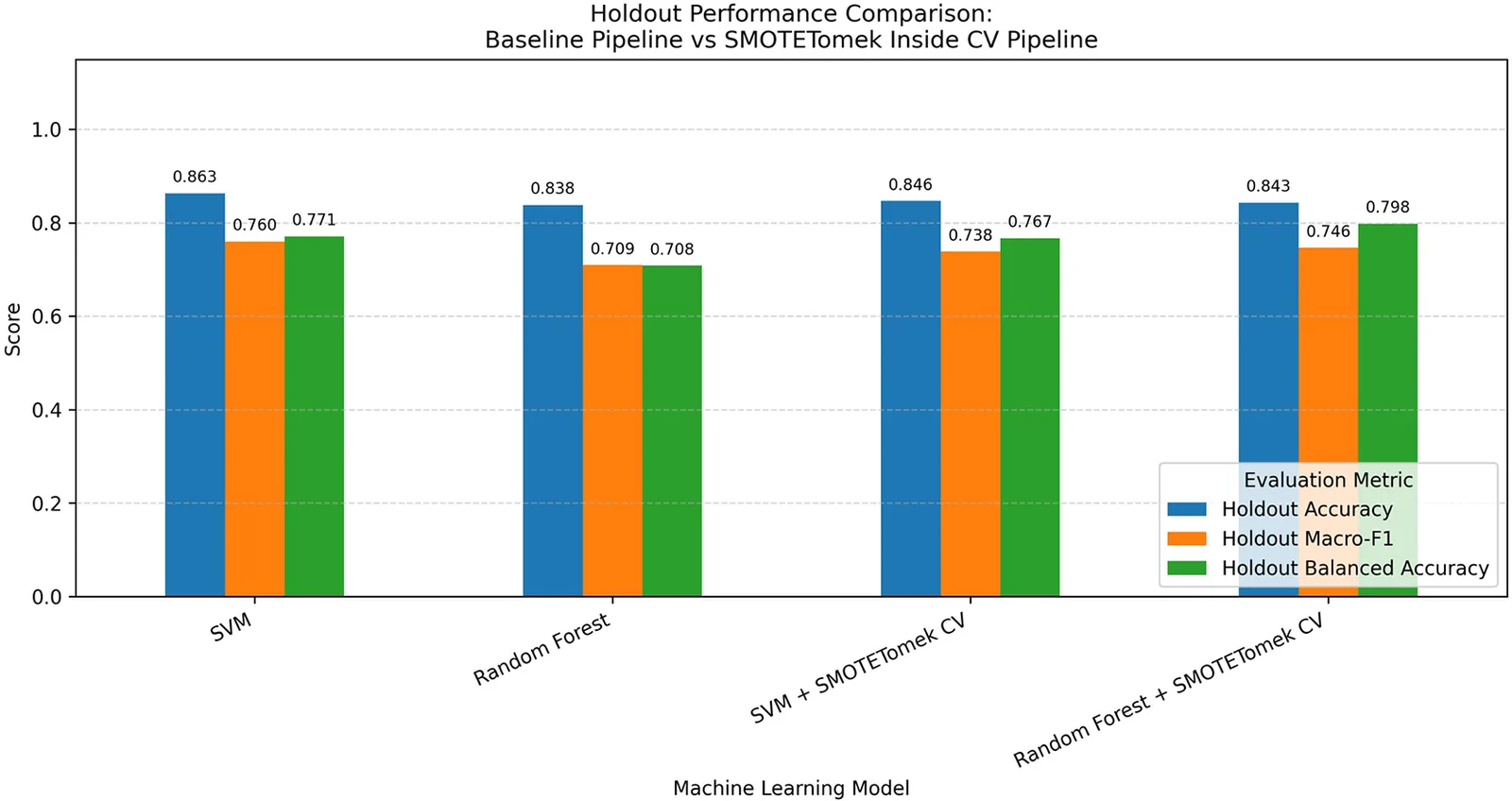
Comparison of the performance of the baseline model versus SMOTETomek on the test data.

Table 7 and Figure 22 compare the performance of SVM and Random Forest before and after applying SMOTETomek in the cross-validation pipeline. For SVM, the application of SMOTETomek slightly reduced accuracy (from 0.8631 to 0.8464) and macro-F1 (from 0.7596 to 0.7383), but did not improve balanced accuracy (from 0.7707 to 0.7669). Conversely, for Random Forest, SMOTETomek improved macro-F1 (from 0.7095 to 0.7462) and balanced accuracy (from 0.7082 to 0.7981), although this was accompanied by a decrease in macro-precision (from 0.7195 to 0.7180). These findings indicate that the benefits of SMOTETomek resampling are model-specific (model-dependent): resampling is more beneficial for the Random Forest model, which is naturally more sensitive to class distribution in the training data, compared to the SVM with a linear kernel, which is already relatively robust to moderate class imbalances.

**Table 7.** Performance comparison: Baseline vs. SMOTETomek (Test/Holdout data).

Model	Accuracy	Macro-averaged F1-Score	Balanced accuracy	Macro-averaged precision	Macro-averaged recall
SVM (baseline)	0.8631	0.7596	0.7707	0.7508	0.7707
SVM + SMOTETomek (CV)	0.8464	0.7383	0.7669	0.7250	0.7669
Random Forest (baseline)	0.8381	0.7095	0.7082	0.7195	0.7082
Random Forest + SMOTETomek (CV)	0.8431	0.7462	0.7981	0.7180	0.7981

## 4. Discussion

### 4.1 Characteristics of user acceptance of skill academy

The predominance of positive sentiment (75.31%) in reviews of the Skill Academy app indicates that, in general, users have a favorable view of this online TVET service, consistent with findings from studies on the acceptance of other online learning apps, which show that flexibility and ease of access are the main drivers of user acceptance. ^73^ ^,^ ^74^ However, the proportion of negative reviews which reached 15.57% cannot be ignored, especially given the finding in Section 3.5 that negative reviews tend to be longer and more detailed than positive reviews. This pattern suggests that dissatisfied users are more motivated to explain their issues in detail, a phenomenon that has also been reported in other studies of mobile app reviews, ^75^ and should be a priority for developers, as longer, more detailed reviews have the potential to contain richer diagnostic information about the root cause of the problem. The results of the keyword analysis show that Skill Academy users’ complaints consistently center on the app’s technical and functional aspects (slow performance, heavy app, system failures/errors), rather than on content or the quality of instruction. Conversely, users’ positive feedback is more closely related to the ease of learning and the benefits of certification. These findings have clear practical implications: efforts to improve user acceptance of Skill Academy are likely to be more effective if focused on optimizing the app’s technical performance (stability, speed, error handling) rather than on adding new learning content, given that content-related issues are relatively rarely complained about.

### 4.2 Sentiment level validity

The moderate level of agreement between VADER and the two independent validation methods (TextBlob: κ = 0.500; star rating: κ = 0.533) provides evidence that the generated sentiment labels are reasonably reliable, though not perfect. This level of agreement, which falls short of the “substantial” or “almost perfect” categories, is consistent with the literature, which indicates that rule-based lexicon methods such as VADER and TextBlob have fundamental differences in their word weighting schemes and handling of negations and intensifiers; therefore, differences in classification results for reviews with mixed or ambiguous sentiment are to be expected. ^27^ Interestingly, VADER’s agreement rate with star ratings (independent of the lexicon-based approach) is actually higher than that of TextBlob, and the monotonically increasing trend in the average compound score relative to star ratings provides convincing evidence of construct validity, suggesting that VADER labels generally reflect users’ actual perceptions. Nevertheless, it is important to emphasize that the performance of the machine learning model reported in this study should be interpreted as the model’s ability to replicate VADER labels, not as a definitive validation of actual user sentiment. This limitation is consistent with common challenges in large-scale sentiment analysis research that relies on automatic labeling. ^76^

### 4.3 Comparison of machine learning model performance

The superiority of logistic regression over other models, both in cross-validation and on the test data, is consistent with various TF-IDF-based text classification studies that show that simple linear models are often competitive with or even outperform more complex models on high-dimensional and sparse text data, particularly when the training dataset is relatively small. ^77^ The use of the ‘class_weight = balanced’ parameter in Logistic Regression also contributes to a higher balanced accuracy compared to SVM and XGBoost, because this parameter explicitly assigns greater weight to classification errors involving the minority class during optimization.

The significantly lower performance of KNN compared to other models (accuracy = 0.5509; macro-F1 = 0.3599) is a finding consistent with the characteristics of the high-dimensional TF-IDF feature space, where Euclidean or cosine distance calculations between documents become less meaningful due to the curse of dimensionality, causing distance-based algorithms such as KNN to tend to perform poorly compared to linear or ensemble models on text data. ^78^ The very low recall for the Negative class in KNN (0.0215) indicates that this model almost always classifies reviews as the majority class or the class whose nearest neighbors are dominated by Positive data a classic weakness of KNN when dealing with imbalanced data.

Boosting models (XGBoost and LightGBM) demonstrated competitive performance but did not outperform Logistic Regression or SVM on the macro-F1 and balanced accuracy metrics, although XGBoost recorded the highest recall for the Positive class. This is likely due to the characteristics of tree-based boosting models, which are naturally better suited for numerical/tabular features than for high-dimensional, sparse text features generated by TF-IDF features that align more closely with the linearity assumptions of models such as Logistic Regression and linear SVM. ^78^

### 4.4 The effectiveness of addressing class imbalances

The results of the SMOTETomek experiment show that the effectiveness of resampling techniques is model-specific. The improvements in balanced accuracy and macro-F1 for Random Forest following the application of SMOTETomek (from 0.7082 to 0.7981 and from 0.7095 to 0.7462) indicate that this tree-based ensemble model benefits significantly from balancing the class distribution in the training data, consistent with the literature, which states that Random Forest tends to be biased toward the majority class when trained on imbalanced data without weight adjustments or resampling. ^69^ ^,^ ^71^ Conversely, the lack of performance improvement in the SVM after applying SMOTETomek indicates that the SVM’s linear kernel with an optimal margin is already relatively robust against moderate class imbalance in this study (a majority-to-minority class ratio of approximately 8:1 for Positive versus Neutral), so adding synthetic data via SMOTE actually has the potential to introduce noise that disrupts the already optimal decision boundary. These findings have important methodological implications for other researchers: the decision to apply resampling techniques should not be applied uniformly across all models, but rather evaluated on a case-by-case basis based on the characteristics of the algorithm used, and ideally always incorporated into the cross-validation pipeline to avoid test data leakage, as was done in this study.

## 5. Conclusions

This study analyzes the sentiment of 2,993 user reviews of the Skill Academy app on Google Play using a text mining approach and a comparison of eight machine learning models with a pipeline designed to be free of data leakage. Three main findings can be summarized. First, user acceptance of Skill Academy is generally positive (75.31% positive reviews), with complaints consistently centered on the app’s technical performance (speed, stability, error handling) rather than its learning content, as confirmed by word cloud analysis, unigram/bigram frequency, and review length patterns—which indicate that negative reviews are written in greater detail than positive ones. Second, the VADER-based automatic sentiment labeling demonstrated reasonably reliable validity, as evidenced by moderate agreement with TextBlob (κ = 0.500) and with the original star rating categories (κ = 0.533), as well as a consistent pattern of increasing average compound scores as the star rating rises. Third, among the eight models compared, Logistic Regression delivered the best overall sentiment classification performance on the test data (accuracy = 85.48%; macro-F1 = 76.08%; balanced accuracy = 80.86%; macro-AUC = 0.9486), outperforming more complex models such as SVM, Random Forest, XGBoost, and LightGBM, while KNN showed the lowest performance due to the high-dimensional nature of the TF-IDF feature space, which is incompatible with the assumption of proximity-based distance. Additional experiments show that the benefits of the SMOTETomek resampling technique are model-specific, providing significant improvements for Random Forest but not for SVM.

Methodologically, this study contributes by applying a TF-IDF pipeline and resampling that are fully integrated into the cross-validation scheme, thereby yielding more valid and reproducible estimates of model performance compared to conventional approaches that perform feature extraction prior to cross-validation. In practical terms, these findings recommend that Skill Academy developers and online TVET stakeholders prioritize improving the technical stability of the application and consider implementing an automated sentiment monitoring system based on the Logistic Regression model as an early-warning mechanism for declining user satisfaction. Future research is recommended to incorporate human annotation, explore transformer-based models for Indonesian text, and expand the scope of the study to include several online TVET platforms to improve the generalizability of the findings. This study provides a comprehensive understanding of Skill Academy’s position in the digital TVET space in Indonesia. By continuing to focus on user feedback and strategic improvements, Skill Academy can strengthen its position as a leading platform for vocational education in Indonesia, contributing to broader digital transformation in the sector. Future research should build on these findings by conducting longitudinal studies to establish causal relationships and test the generalizability of these results across other platforms and education sectors.

## Ethical considerations

This study did not involve direct interaction with human participants. The research exclusively analyzed publicly available user reviews obtained from the Google Play Store for the Skill Academy application. All data were collected in accordance with the platform’s publicly accessible interfaces, and no private, sensitive, or personally identifiable information was collected, stored, or analyzed. Usernames and any identifiable information were excluded during data processing, and all analyses were conducted in aggregate form. As the study relied solely on secondary, publicly available data, formal ethical approval and informed consent were not required, in line with institutional research ethics guidelines and established standards for social science research using public online data.

## Author contribution

D.D = Conceptualization, Formal Analysis, Funding Acquisition, Methodology, Resources, Supervision, Validation, Writing – Review & Editing; Y.A.F = Formal Analysis, Investigation, Methodology, Resources, Writing – Original Draft Preparation, Validation, Writing – Review & Editing; K.M = Formal Analysis, Investigation, Methodology, Resources, Software, Validation, Writing – Review & Editing; M.R.M = Data Curation, Formal Analysis, Writing – Original Draft Preparation, Resources; A.A.S = Data Curation, Formal Analysis, Funding Acquisition, Project Administration, Resources; RBS = Funding Acquisition, Project Administration, Resources, Visualization; B.B.B = Funding Acquisition, Methodology, Validation, Visualization. R.J.S = Data Curation, Formal Analysis, Investigation, Methodology, Software, Visualization, Writing – Original Draft Preparation, Writing – Review & Editing; S. S = Funding Acquisition, Project Administration, Validation, Writing – Review & Editing. All authors have read and agreed to the published version of the manuscript.

## Data Availability

The datasets supporting the findings of this study are available in the Zenodo repository under an open license. The shared data include anonymized and curated outputs derived from publicly available user reviews of the Skill Academy application on the Google Play Store, along with sentiment labels, processed text features, and data used to generate figures and tables reported in this article. Raw user reviews cannot be redistributed due to the Google Play Store terms of service. However, all preprocessing steps and data transformation procedures are fully documented to ensure reproducibility. The datasets supporting the findings of this study are available in the Zenodo repository ^81^ at: https://doi.org/10.5281/zenodo.19916911 (Version 1); http://doi.org/10.5281/zenodo.21307408 (Version 2). Source code available from: https://github.com/yanuaragung2025/Yanuar-Agung-Fadlullah/tree/1. Archived software available from: https://doi.org/10.5281/zenodo.19916911. ^81^ License: MIT License. This study reports findings from a quantitative text mining and machine learning analysis of user reviews obtained from a mobile application platform. As the study does not involve clinical trials, systematic reviews, experimental interventions, or in vivo research, no specific reporting guideline checklist such as CONSORT, PRISMA, or ARRIVE applies. The manuscript has been prepared to ensure transparency and reproducibility by clearly reporting data sources, preprocessing steps, analytical methods, model evaluation procedures, and study limitations, in accordance with best practices for computational social science and machine learning research.
