# Supplementary material for: Sentiment Analysis of Acceptance TVET Online Courses on the Skill Academy App from Google Play: Leveraging Text Mining with Comparison Machine Learning Model
Source: F1000Res. 2026 Sep 24;15:899. Originally published 2026 Jun 9. [Version 2] doi: 10.12688/f1000research.181728.2 (PMC13310357; doi:10.12688/f1000research.181728.2)
Supplement: Peer review history

# Reviewer report: Reviewer response for version 2

**DOI:** 10.5256/f1000research.206313.r524223

**Published:** 2026-09-25

**Authors:** Febblina Daryanes

**Affiliations:** 1 Riau University, Pekanbaru, Riau, Indonesia

I have no further comments. The authors have adequately addressed my previous concerns and recommendations.

Is the work clearly and accurately presented and does it cite the current literature?

Partly

If applicable, is the statistical analysis and its interpretation appropriate?

I cannot comment. A qualified statistician is required.

Are all the source data underlying the results available to ensure full reproducibility?

Yes

Is the study design appropriate and is the work technically sound?

Partly

Are the conclusions drawn adequately supported by the results?

Yes

Are sufficient details of methods and analysis provided to allow replication by others?

Partly

Reviewer Expertise:

NA

I confirm that I have read this submission and believe that I have an appropriate level of expertise to confirm that it is of an acceptable scientific standard.

# Reviewer report: Reviewer response for version 1

**DOI:** 10.5256/f1000research.200603.r493709

**Published:** 2026-06-27

**Authors:** Febblina Daryanes

**Affiliations:** 1 Riau University, Pekanbaru, Riau, Indonesia

1. The manuscript is generally well organized and easy to follow. The introduction, methodology, results, discussion, and conclusion are presented in a logical sequence, and the authors cite several recent references related to sentiment analysis, machine learning, and digital TVET. However, the manuscript still relies heavily on descriptive reporting of results and lacks sufficient critical engagement with the most recent advances in sentiment analysis, particularly transformer-based language models (e.g., BERT, RoBERTa, IndoBERT) and explainable AI techniques. The research gap is also not sufficiently established beyond the application domain. 

 The authors state that no previous studies have investigated the Skill Academy platform. However, this does not constitute a sufficient scientific research gap. A research gap should be established by identifying limitations in previous studies, such as methodological weaknesses, shortcomings of existing algorithms, unresolved research questions, or inconsistencies in the literature, rather than merely emphasizing a different application domain or study object. The authors are encouraged to strengthen the Introduction by clearly explaining how the present study advances existing knowledge beyond applying established methods to a new platform.

 2. The overall study design is appropriate for conducting sentiment analysis using publicly available Google Play Store reviews. The preprocessing pipeline and the comparative evaluation of six machine learning algorithms are generally well implemented.

 However, several methodological limitations reduce the scientific rigor of the study. First, the sentiment labels were automatically generated using VADER after machine translation rather than being manually annotated by human reviewers. Consequently, the machine learning models were trained to learn VADER-generated labels instead of validated ground-truth annotations, which may introduce labeling bias and limit the validity of the classification results. Second, the manuscript does not report any hyperparameter tuning procedure for the evaluated classifiers. Third, model evaluation relies solely on a single train-test split without cross-validation, which may produce performance estimates that are sensitive to the particular data partition.

 Addressing these methodological issues would substantially strengthen the scientific rigor, reliability, and reproducibility of the study.

 3. The manuscript provides a general description of the methodology. However, several details necessary for full reproducibility are missing, including preprocessing settings, TF-IDF configuration, model hyperparameters, random seed, software versions, and the exact train-test split procedure. Providing these details would improve the reproducibility of the study.

Is the work clearly and accurately presented and does it cite the current literature?

Partly

If applicable, is the statistical analysis and its interpretation appropriate?

I cannot comment. A qualified statistician is required.

Are all the source data underlying the results available to ensure full reproducibility?

Yes

Is the study design appropriate and is the work technically sound?

Partly

Are the conclusions drawn adequately supported by the results?

Yes

Are sufficient details of methods and analysis provided to allow replication by others?

Partly

Reviewer Expertise:

NA

I confirm that I have read this submission and believe that I have an appropriate level of expertise to confirm that it is of an acceptable scientific standard, however I have significant reservations, as outlined above.

# Response

**Published:** 2026-07-13

**Authors:** 196408051991011001 darmono

**Reviewer 3: Dr. Febblina Daryanes, M.Pd**

 Reviewer 3 Comment 1:

 The manuscript remains largely descriptive and lacks critical engagement with recent developments in sentiment analysis, particularly transformer-based models (e.g., BERT, RoBERTa, IndoBERT) and Explainable Artificial Intelligence (XAI) techniques.

**Response from Author to Reviewer 3 Comment 1:**

 The Discussion and Limitations sections were expanded to critically compare TF-IDF and classical machine learning approaches with transformer-based models such as BERT, RoBERTa, and IndoBERT. The potential application of Explainable Artificial Intelligence (XAI) was also discussed as a promising direction for future research.

 Reviewer 3 Comment 2:

 The novelty of the study is primarily based on the application domain (Skill Academy) rather than a clearly articulated methodological or theoretical research gap.

**Response from Author to Reviewer 3 Comment 2:**

 The Introduction was strengthened by explicitly identifying methodological gaps in prior studies, including limited validation of automatically generated sentiment labels, insufficient use of robust evaluation procedures such as cross-validation and hyperparameter optimization, and inadequate handling of imbalanced datasets. Consequently, the study’s contribution extends beyond the application domain itself.

 Reviewer 3 Comment 3:

 Sentiment labels were automatically generated by VADER following machine translation without human annotation, meaning that the machine learning models learned from VADER-generated labels rather than validated ground-truth labels.

**Response from Author to Reviewer 3 Comment 3:**

 Two independent validation procedures were incorporated (VADER versus TextBlob and VADER versus user star ratings) using Cohen’s Kappa statistics. The absence of human annotation is explicitly acknowledged as a limitation, and future studies involving human-labeled datasets are recommended. Two independent validation procedures were added: (a) VADER versus TextBlob agreement analysis using Cohen’s Kappa, and (b) comparison between VADER labels and users’ original star ratings through cross-tabulation, heatmaps, and Cohen’s Kappa. The absence of human annotation remains explicitly acknowledged as a limitation, and future research employing human-labeled samples is recommended.

 Reviewer 3 Comment 4:

 Hyperparameter tuning procedures for the evaluated classification models were not reported.

**Response from Author to Reviewer 3 Comment 4:**

 An explicit GridSearchCV procedure was implemented for all models. The optimal hyperparameter combinations are now reported in the notebook output and stored in CSV tables for reproducibility. GridSearchCV was applied to all models, and the resulting optimal hyperparameter settings are now reported and archived in CSV format.

 Reviewer 3 Comment 5:

 Model evaluation relied solely on a single train-test split, making performance estimates sensitive to a specific data partition.

**Response from Author to Reviewer 3 Comment 5:**

 Stratified K-Fold cross-validation (k = 5) was implemented for all models, and performance metrics are reported as mean ± standard deviation.

 Reviewer 3 Comment 6:

 Important details required for reproducibility, including preprocessing settings, TF-IDF configuration, hyperparameters, random seeds, software versions, and train-test splitting procedures, were insufficiently documented.

**Response from Author to Reviewer 3 Comment 6:**

 A dedicated reproducibility section was added, documenting RANDOM_STATE = 42, software versions, preprocessing configurations, TF-IDF settings (fitted exclusively on training data), GridSearchCV-derived hyperparameters, and the complete train-test splitting procedure to ensure full reproducibility of the study.

# Reviewer report: Reviewer response for version 1

**DOI:** 10.5256/f1000research.200603.r493713

**Published:** 2026-06-24

**Authors:** Sriyanti Mustafa

**Affiliations:** 1 Universitas Muhammadiyah Parepare, South Sulawesi, Indonesia

This manuscript analyzes 3,000 Google Play reviews of the Skill Academy online TVET application by applying VADER sentiment analysis and comparing six machine-learning classifiers. The topic is relevant, the comparison of multiple models is useful, and the availability of data and code is a positive contribution. The results show that most reviews were positive and that SVM achieved the highest accuracy. However, several methodological issues must be addressed before the article can be accepted.

 The main concern is that the sentiment labels generated by VADER were also used as the target labels for training and testing the machine-learning models. Therefore, the reported performance mainly reflects agreement with VADER rather than independently validated user sentiment. The authors should validate a representative sample using human annotators, reconsider the preprocessing and translation procedure, and ensure that TF-IDF vectorization is performed only after splitting the training and test data to avoid data leakage. The evaluation should also be strengthened through stratified cross-validation, complete reporting of hyperparameters, and greater emphasis on macro-F1, balanced accuracy, and class-specific recall because the dataset is imbalanced.

 The authors should also correct the interpretation of multiclass confusion matrices and ROC-AUC, provide clearer details on data collection and reproducibility, remove identifiable usernames from Figure 3, and carefully edit the English language. In addition, positive sentiment should not be directly interpreted as technology acceptance because established acceptance constructs were not measured. The manuscript has indexing potential, but substantial methodological clarification, validation, and reanalysis are required. I therefore recommend Approved with Reservations, subject to major revision.

Is the work clearly and accurately presented and does it cite the current literature?

Partly

If applicable, is the statistical analysis and its interpretation appropriate?

No

Are all the source data underlying the results available to ensure full reproducibility?

Partly

Is the study design appropriate and is the work technically sound?

No

Are the conclusions drawn adequately supported by the results?

Partly

Are sufficient details of methods and analysis provided to allow replication by others?

Partly

Reviewer Expertise:

Educational Technology, Digital Learning, Learning Analytics, and Educational Research Methodology.

I confirm that I have read this submission and believe that I have an appropriate level of expertise to confirm that it is of an acceptable scientific standard, however I have significant reservations, as outlined above.

# Response

**Published:** 2026-07-13

**Authors:** 196408051991011001 darmono

**Reviewer 2: Prof. Dr. Sriyanti Mustafa, M.Pd**

**Reviewer 2 Comment 1:**

 VADER-generated sentiment labels were used as both training and testing targets for the machine learning models, meaning that the reported accuracy reflected agreement with VADER rather than independent validation of actual user sentiment.

**Response from Author to Reviewer 2 Comment 1:**

 Two independent validation procedures were added: (a) VADER versus TextBlob agreement analysis using Cohen’s Kappa, and (b) comparison between VADER labels and users’ original star ratings through cross-tabulation, heatmaps, and Cohen’s Kappa. The absence of human annotation remains explicitly acknowledged as a limitation, and future research employing human-labeled samples is recommended.

**Reviewer 2 Comment 2:**

 TF-IDF vectorization should be performed only after splitting the data into training and testing sets to avoid data leakage.

**Response from Author to Reviewer 2 Comment 2:**

 The entire analytical pipeline, including classical machine learning models, XGBoost, and LightGBM, was revised so that TF-IDF is fitted exclusively on the training data and subsequently applied to the testing data, eliminating the possibility of data leakage.

**Reviewer 2 Comment 3:**

 Model evaluation should be strengthened using stratified cross-validation rather than a single train-test split.

**Response from Author to Reviewer 2 Comment 3:**

 Stratified K-Fold cross-validation (k = 5) was implemented for all models, with results reported as mean ± standard deviation across folds.

**Reviewer 2 Comment 4:**

 Hyperparameter configurations should be fully reported to enhance reproducibility.

**Response from Author to Reviewer 2 Comment 4:**

 An explicit GridSearchCV procedure was implemented for all models. The optimal hyperparameter combinations are now reported in the notebook output and stored in CSV tables for reproducibility.

**Reviewer 2 Comment 5:**

 Greater emphasis should be placed on macro-F1, balanced accuracy, and class-wise recall due to the imbalanced nature of the dataset.

**Response from Author to Reviewer 2 Comment 5:**

 Macro-F1, balanced accuracy, and class-wise recall are now comprehensively reported for all models. Additional experiments using SMOTETomek resampling and class_weight='balanced' were conducted to assess performance robustness on minority classes.

**Reviewer 2 Comment 6:**

 The interpretation of the multiclass confusion matrix and ROC-AUC metrics in the previous manuscript was not fully appropriate.

**Response from Author to Reviewer 2 Comment 6:**

 ROC-AUC calculations and interpretations were revised using the correct One-vs-Rest (OvR) multiclass framework. Precision–Recall curves for each class (OvR) were also added for the best-performing model.

**Reviewer 2 Comment 7:**

 Additional details regarding data collection procedures and reproducibility, including random seeds and software versions, should be provided.

 Response from Author to Reviewer 2 Comment 7:

 A dedicated reproducibility section was added to the notebook, establishing RANDOM_STATE = 42 throughout the workflow and reporting the versions of all libraries used, including pandas, NumPy, scikit-learn, XGBoost, LightGBM, Matplotlib, and Seaborn.

**Reviewer 2 Comment 8:**

 Usernames displayed in Figure 3 may reveal user identities and should be removed to protect privacy.

**Response from Author to Reviewer 2 Comment 8:**

 The userName field was anonymized through hashing at the beginning of the data-processing pipeline. Consequently, no personally identifiable information appears in any figure or table presented in the revised manuscript and notebook.

**Reviewer 2 Comment 9:**

 The English language throughout the manuscript requires careful editing.

**Response from Author to Reviewer 2 Comment 9:**

 The manuscript was thoroughly reviewed and edited to improve grammar, clarity, readability, and consistency of technical terminology throughout all sections.

**Reviewer 2 Comment 10:**

 Positive sentiment should not be directly interpreted as technology acceptance because formal technology acceptance constructs were not measured.

**Response from Author to Reviewer 2 Comment 10:**

 This limitation has been explicitly acknowledged in the Limitations section. The manuscript now clarifies that the study measures user sentiment rather than formal technology acceptance constructs such as TAM or UTAUT, and recommends future studies that directly assess technology acceptance.

# Reviewer report: Reviewer response for version 1

**DOI:** 10.5256/f1000research.200603.r493706

**Published:** 2026-06-24

**Authors:** Rajiansyah Rajiansyah

**Affiliations:** 1 Universitas Mulawarman, Samarinda, East Kalimantan, Indonesia

Overall, this manuscript presents a valuable contribution to the field of educational technology and sentiment analysis. The study demonstrates clear novelty by focusing on Indonesian TVET applications, an area that has received limited attention in previous research. The authors provide a comprehensive comparison of several machine learning models and offer methodological and practical insights to support future research and the development of applications in emerging markets.

 The research methodology is appropriate and follows established practices for machine-learning-based sentiment analysis. The workflow is clearly described, from data collection and preprocessing to model training and evaluation. The inclusion of multiple classification models and several performance metrics strengthens the reliability of the findings and supports a thorough assessment of model performance.

 The manuscript is well supported by relevant figures, tables, and references. The visual materials help readers understand the research process and results, while the literature review demonstrates a solid understanding of the existing body of knowledge. The references are appropriate and contribute to the transparency and reproducibility of the study.

 One minor limitation is the reliance on translation before sentiment analysis, as translation may introduce subtle changes in meaning that can affect sentiment classification. However, using a widely accepted tool such as VADER helps mitigate this concern and is a reasonable approach when working with non-English datasets. I identified two minor errors in the manuscript that should be corrected before indexing:

1. **Description of Support Vector Machine (SVM):** In the Introduction section, the description of Support Vector Machine (SVM) appears to be a direct repetition of the description provided for Naïve Bayes. As a result, the characteristics of SVM are not accurately presented. While Naïve Bayes is commonly recognized for its simplicity and computational efficiency, SVM is better known for its ability to construct optimal separating hyperplanes and achieve strong performance in high-dimensional feature spaces. I recommend revising this paragraph to reflect the distinctive characteristics and strengths of SVM.
2. **Terminology Used for VADER Sentiment Scoring: **The manuscript uses the term "combined score" when explaining the sentiment classification thresholds generated by VADER. However, the standard term in VADER is "compound score," which refers to the normalized, weighted composite sentiment score used for classification. The manuscript already uses the correct term in the mathematical explanation and in the cited reference. Therefore, I suggest replacing "combined score" with "compound score" throughout the manuscript to maintain consistency and avoid potential confusion for readers.

I recommend acceptance after minor revisions to address the comments provided above, which will help improve the clarity and presentation of the work.

Is the work clearly and accurately presented and does it cite the current literature?

Yes

If applicable, is the statistical analysis and its interpretation appropriate?

Yes

Are all the source data underlying the results available to ensure full reproducibility?

Yes

Is the study design appropriate and is the work technically sound?

Yes

Are the conclusions drawn adequately supported by the results?

Yes

Are sufficient details of methods and analysis provided to allow replication by others?

Yes

Reviewer Expertise:

Decision Theory, Game Theory, Sentiment Analysis, Machine Learning, Programming.

I confirm that I have read this submission and believe that I have an appropriate level of expertise to confirm that it is of an acceptable scientific standard.

# Response

**Published:** 2026-07-13

**Authors:** 196408051991011001 darmono

**Reviewer 1: Rajiansyah, M.Sc., Ph.D**

**Reviewer 1 Comment 1:**

 The description of Support Vector Machine (SVM) in the Introduction duplicated the description of Naïve Bayes, failing to accurately reflect the distinctive characteristics of SVM, such as optimal separating hyperplanes and strong performance in high-dimensional feature spaces.

**Response from Author to Reviewer 1 Comment 1:**

 The SVM description in the Introduction was revised to distinguish it from the Naïve Bayes description. The revised text now highlights SVM’s ability to construct optimal separating hyperplanes and its effectiveness in high-dimensional feature spaces, while the Naïve Bayes section continues to emphasize computational simplicity and efficiency.

**Reviewer 1 Comment 2:**

 The term “combined score” used to explain VADER sentiment classification thresholds was inconsistent with the standard term “compound score” used in the mathematical formulation and cited references.

**Response from Author to Reviewer 2 Comment 2:**

 All occurrences of “combined score” were replaced with “compound score” throughout the manuscript and analytical code to ensure consistency with the mathematical formulation and referenced literature.
